# A Systematic Review of Glioblastoma-Targeted Therapies in Phases II, III, IV Clinical Trials

**DOI:** 10.3390/cancers13081795

**Published:** 2021-04-09

**Authors:** Elisabete Cruz Da Silva, Marie-Cécile Mercier, Nelly Etienne-Selloum, Monique Dontenwill, Laurence Choulier

**Affiliations:** 1 CNRS, UMR 7021, Laboratoire de Bioimagerie et Pathologies, Faculté de Pharmacie, Université de Strasbourg, 67401 Illkirch, France; elisabete.silva@unistra.fr (E.C.D.S.); marie.071@hotmail.fr (M.-C.M.); nelly.etienne-selloum@unistra.fr (N.E.-S.); monique.dontenwill@unistra.fr (M.D.); 2 Service de Pharmacie, Institut de Cancérologie Strasbourg Europe, 67200 Strasbourg, France

**Keywords:** glioblastoma, targeted therapies, biomarkers, clinical trials

## Abstract

**Simple Summary:**

This review describes in a very detailed and exhaustive approach the literature of these last 20 years on glioblastoma targeted therapies in Phases II-IV of 257 clinical trials on adults with newly diagnosed or recurrent GBMs (excluding targeted immunotherapies and therapies targeting tumor cell metabolism, well documented in recent reviews). Divided in four Sections, are provided descriptions and lists (in 12 different tables) of, not only main but all drugs, targets, clinical trials and the results of targeted therapies when they are known.

**Abstract:**

Glioblastoma (GBM), the most frequent and aggressive glial tumor, is currently treated as first line by the Stupp protocol, which combines, after surgery, radiotherapy and chemotherapy. For recurrent GBM, in absence of standard treatment or available clinical trials, various protocols including cytotoxic drugs and/or bevacizumab are currently applied. Despite these heavy treatments, the mean overall survival of patients is under 18 months. Many clinical studies are underway. Based on clinicaltrials.org and conducted up to 1 April 2020, this review lists, not only main, but all targeted therapies in phases II-IV of 257 clinical trials on adults with newly diagnosed or recurrent GBMs for the last twenty years. It does not involve targeted immunotherapies and therapies targeting tumor cell metabolism, that are well documented in other reviews. Without surprise, the most frequently reported drugs are those targeting (i) EGFR (40 clinical trials), and more generally tyrosine kinase receptors (85 clinical trials) and (ii) VEGF/VEGFR (75 clinical trials of which 53 involving bevacizumab). But many other targets and drugs are of interest. They are all listed and thoroughly described, on an one-on-one basis, in four sections related to targeting (i) GBM stem cells and stem cell pathways, (ii) the growth autonomy and migration, (iii) the cell cycle and the escape to cell death, (iv) and angiogenesis.

## 1. Introduction

**Figure 1:**
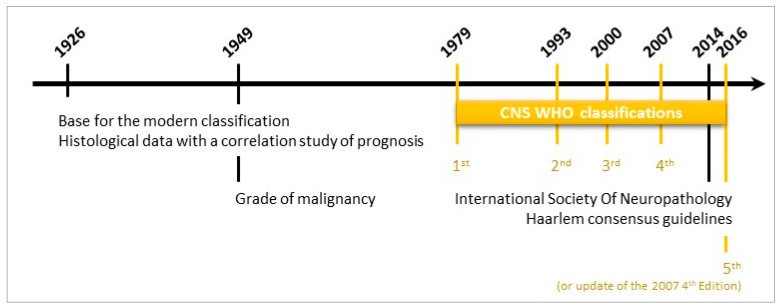
Timeline showing the principal dates of the histological and molecular classifications of gliomas. Classifying brain tumors has been the subject of many studies for several years. The first classification published in 1926 by Bailey and Cushing was based on histogenetics [51]. According to this classification, the presence of embryonic cells would be at the origin of tumor cells. The second classification proposed in 1949 by Kernohan JW, Mabon [52], includes grades of malignancy. The WHO proposed a new classification of gliomas in 1979 [53], which is internationally recognized and was revised in 1993, 2000, 2007 and 2016 [54,55,56,57]. These classifications are based on anatomopathological analysis of a representative glioma fragment (from biopsy or surgical resection) and “grading” elements. The International Society of Neuropathology was held from 1–3 May 2014 in Haarlem, the Netherlands [58]. The meeting reached consensus regarding the incorporation of non-histological data, such as molecular information, into the next WHO classification [55].

Since 1926, different classifications of brain tumors have been proposed, based mainly on histological and malignancy criteria [1]. Increasing knowledge on glioma molecular characteristics enabled the proposition of a new classification in 2016. Figure 1 recapitulates the main steps of the modern classification of gliomas. Glioblastoma (GBM) is a high-grade glioma (grade IV), the most aggressive and the most frequent glioma. In the 2016 classification, GBMs are divided into three groups according to the status of the isocitrate dehydrogenase (IDH) gene: (i) GBMs IDHwt [this group represents 90% of GBMs and corresponds to primary GBMs], (ii) mutated IDH GBMs [this group represents 10% of GBMs, corresponds to secondary GBMs, occurs in young patients and has a better prognostic], (iii) Not otherwise specified (NOS) GBMs [status could not be evaluated]. When histological data suggest GBM and immunohistochemical analysis of IDHmut is negative, sequencing is recommended. Sequencing is no longer recommended after the age of 55 [2]. Inhibitors of the mutated IDH proteins are currently evaluated in GBM in Phase I clinical trials (NCT02073994, NCT02273739). They will thus not be further described in this review.

The standard treatment of GBMs is based on surgical resection followed by radiotherapy (RT) and concomitant chemotherapy for 6 weeks. The area around the tumor is irradiated with 2 Gy per day, five days per week for a total dose of 60 Gy. The chemotherapy used is Temozolomide (TMZ) at 75 mg/m^2^ per day. After this radiochemotherapy, TMZ treatment is pursued alone every four weeks at 150–200 mg/m^2^ per day for 5 consecutive days [3]. TMZ is an alkylating agent that causes DNA damage, cell cycle arrest and cell apoptosis. After oral administration, it is spontaneously hydrolyzed into an highly instable metabolite: 3-methyl-(triazen-1-yl)imidazole-4-carboxamide (MTIC) which reacts with water and releases highly reactive 5-aminoimidazole-4-carboxamide and methyldiazonium. The latter induces methylation at the O6 and N7 positions of a guanine and N3 position of an adenine [4]. These mutations cause aberrant repairs.

Despite these aggressive treatments, recurrence generally appears within 6–9 months of diagnosis [5]. In 90% of cases, recurrence is at the edge of the surgical resection. At the appearance of recurrence, patients’ survival is low: 3–6 months [6,7]. No protocol has yet been validated in the management of recurrent GBM. An increase in RT doses does not lead to gain in survival but induces more toxicity, including necrosis of healthy tissue [8]. Long-term side effects of radiation exposure (among which neurocognitive, psychosocial, endocrine …) are present months or years after treatment and cause problems in rare people who survive as the effect of side-effects increases with time [9]. Increasing the doses of TMZ is also not more efficient [10]. In most cases, patients with recurrent GBM are included in clinical trials [11]. If not, several therapeutic molecules are proposed in the second line, mainly alkylating agents (lomustine, carmustine, fotemustine, carboplatin or procarbazine), microtubule destabilizing agent (vincristine) or antiangiogenic drug (bevacizumab). In absence of standard protocols, the therapeutic strategy is discussed for individual patients. In addition, corticosteroids, anticonvulsants (lacosamide, levetiracetam) and anticoagulants are used in the progression of tumors in the event of intracranial pressure, stroke and deep venous thrombosis epilepsy which occurs in 30% of patients with primary brain tumors [12].

Different improvements of the current protocol (surgery, radio and chemo therapies) or new strategies based on the particular microenvironment of GBM are increasingly proposed for the effective care of GBM [13,14,15,16,17]. They are briefly mentioned below, but are not the focus of this review. But regardless of strategies, if new treatments allowed for significantly longer survival, they would require more than improving patients’ survival and would minimize long-term side effects to preserve or even improve patients’ quality of life. Late adverse events induced by administered treatments should be addressed [18].

New strategies are proposed to improve the drug passage through the blood brain barrier (BBB) to achieve a higher therapeutic concentration at the tumor site. Delivering chemotherapy directly into the surgical resection cavity has been proposed. Convection-enhanced delivery (CED) allows chemotherapy to be delivered directly via a catheter in the tissue surrounding the GBM resection cavity. This method increases the volume of distribution but results in unpredictable brain diffusion [19]. It requires the use of several surgical procedures, leading to a high risk of infection or bleeding. Another strategy consists of administrating the therapy directly at the tumor resection bed [20,21,22,23,24,25]. The use of small lipophilic molecules, able to passively cross the endothelial cells of the BBB, has been tested in combination with standard therapies [26]. Encapsulating therapies in nanoparticles (10–200 nm) not only increases their solubility but also their release time and stability, while reducing side effects [27,28].

GBM has long been considered as a non-immunogenic tumor due to immunosuppressive adaptation mechanisms, low levels of T cells, dendritic cells and monocytes, decreased IgG and IgA and increased regulatory T cells [29]. Many different recent reviews focus on novel therapies that harness the immune system, including vaccination, T-cell therapies, immune check-point modulators or adaptive immunotherapy [30,31,32,33].

Targeting tumor cell metabolism is another option. GBM is a hypoxic tumor. Hypoxia plays a role via different hypoxia inducing factors, HIF-1α and HIF-2α [34]. HIF1-α or factors implicated in the HIFs pathways have been proposed as potential therapeutic targets (as for examples profilin-1 or FIH1) [35,36,37]. To date, one Phase II clinical trial has been performed via the inhibitor of HIF2α, PT2385 [38] (NCT03216499).

Approaches aiming to exploit the metabolic deregulation of tumor cells compared to healthy cells are also increasing and characterization of specific metabolic pathways and metabolites are under intense investigations. Tumor cells have an increased need for glucose compared to healthy cells [39]. Thus, unlike healthy cells that use mitochondrial oxidative phosphorylation to generate ATP, tumor cells use aerobic glycolysis (the “Warburg effect”) [40]. Based on this concept, reduction of glucose delivery to tumor cells, for example, might influence their growth without influencing normal cells [41].

Delivery of low-intensity, intermediate-frequency (100–300 kHz) alternating electric fields through the TTFields, Optune^®^, Novocure Inc., Portsmouth, NH USA (tumor treatment fields) device has given an alternative strategy to treat GBM. It was approved by the FDA since 2011 for recurrent GBM. Beside antiproliferative and anti-mitotic effects, this device efficacy might also be related to inhibition of migration, invasion, angiogenesis and DNA repair as well as induction of apoptosis and immune effects [42].

GBMs are characterized by a high molecular and transcriptional inter- and intra-tumoral heterogeneity [43,44,45,46]. Developments in multi-omic analysis have led to identification of specific molecular signatures [47,48,49] discriminating at least 3 different subclasses (mesenchymal, proneural and classical) but also emphasized a high degree of plasticity between cellular states [50]. Nevertheless, proposition of targeted therapies has increased these last years based on promising preclinical data which supported the initiation of clinical trials. The aim of this paper is to make an exhaustive review of the different clinical trials (completed or under way) focusing on drugs considered as targeted therapeutics. We have divided the topic in 4 different sections considering drugs inhibiting (1) stem cells and stem cell pathways (Section 3.1), (2) the growth autonomy and migration (Section 3.2), (3) the cell cycle and escape to cell death (Section 3.3) and (4) angiogenesis (Section 3.4). Clinical trials of phases I/II, II, III or IV have been considered but not those of Phase I.

## 2. Methods

**Figure 2:**
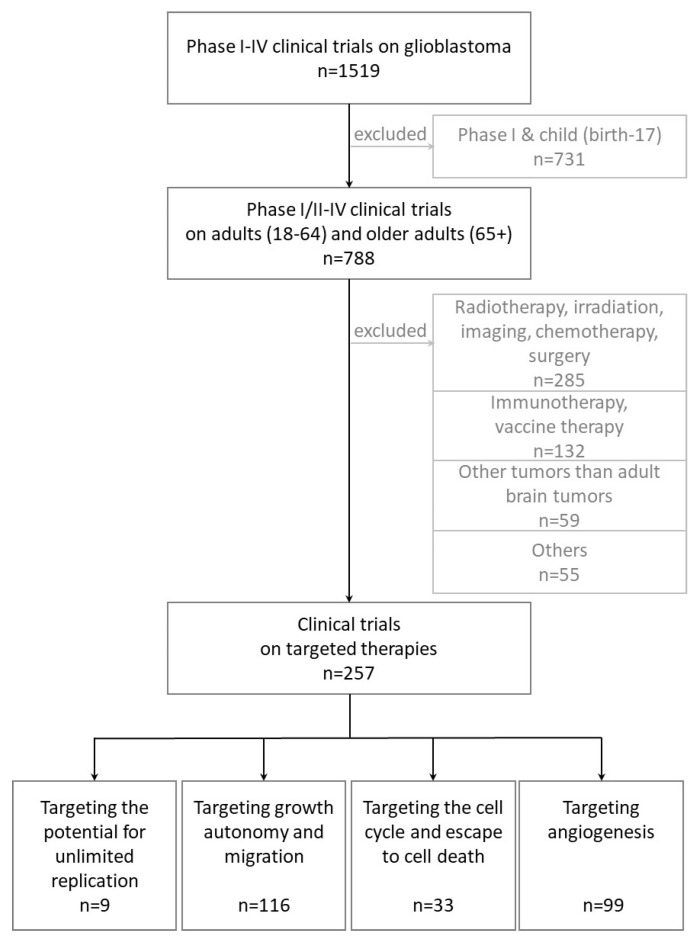
Flowchart.

1 April 2020 has been set as the end date for data collection for this study. The flowchart (Figure 2) lists the clinical trials included and excluded from this manuscript. Briefly, 1519 clinical trials were listed on www.clinicaltrials.com (accessed on 1 April 2021) for GBM. Restrictions were applied to keep only clinical trials on adults and phases I/II to IV. 788 clinical trials remained (212 Phase I/II, 488 Phase II, 14 Phase II/III, 70 Phase III & 4 Phase IV). They have then been sub-classified: 257 clinical trials concerning targeted therapies are described in this review, and 531 clinical trials were excluded from this analysis as they are related to (i) RT, irradiation, imaging, classic cytotoxic chemotherapy, surgery, (ii) immunotherapy and vaccine therapy, (iii) other tumors than adult brain tumors, and (iv) other studies, such as withdrawal trials, trials which did not retain enough patients or did not pass phase II, studies on hypoxia, metabolism, anti-depressants, vitamins, hormones, molecules for sleep disorders, or cognitive decline, or drugs for which molecular targets are not clearly identified.

To recapitulate, the 257 clinical trials described in this review cover 20-years of targeted therapies in clinical phases I/II and over, for adult GBM. In addition to GBM, clinical trials including gliomas, high grade gliomas, gliosarcomas, anaplastic astrocytomas, or other brain tumors were retained. Children and young patient brain tumors were excluded.

Twelve tables detail the different clinical trials underway or completed in phases I/II, II, III or IV. The dates mentioned correspond to the start of the clinical trial and the last date of data update on Clinicaltrials.com. In tables, comparative trials with a significant difference between two treatments are highlighted in green and those with a non-significant difference are highlighted in red.

## 3. Results-Glioblastoma Targeted Therapies

**Figure 3:**
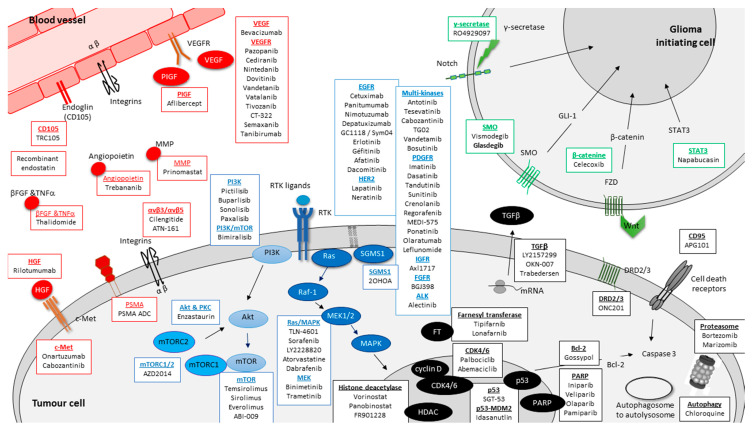
Principal biomarkers and drugs in GBM targeted therapies. The color code corresponds to the four sections of the Results section. The targeting of stem cells and stem cell pathways is represented in green, the targeting of growth autonomy and migration in blue, the targeting of the cell cycle & escape to cell death in black and the targeting of angiogenesis in red. Acronyms are defined in the text.

The different GBM biomarkers targeted in phases I/II, II, III and IV and described in the following paragraphs are presented in Figure 3.

### 3.1. Targeting Stem Cells and Stem Cell Pathways

The discovery of tumor stem-like cells in solid tumors including glioma [59,60] has changed the landscape of the origin of tumors and their recurrence. These cells also named “GBM initiating cells” (GICs) or “GBM stem cells” (GSC) [61,62] exhibit self-renewal capacity and differentiating ability to form the tumoral mass [63]. The presence of GICs can be explained by the malignant transformation of neural (non-tumor) stem cells [64] and/or by the de-differentiation of tumor cells into tumor stem cells following radiotherapy or chemotherapy [65]. 

GICs are reported to be more resistant to current treatments than differentiated tumor cells explaining their role in GBM recurrence. This increased resistance can be explained by (1) a quiescent condition, resulting in the ineffectiveness of currently used chemotherapies targeting the cell cycle [66], (2) High expression of efflux transporters, including MRP1 (Multidrug resistance-associated protein 1) and P-gP (Permeability-GlycoProtein), evicting therapeutic molecules and (3) a defective regulation of apoptosis, with higher expression of survival factors and an ability to adapt to a stressful environment [67]. 

The discovery of GICs has generated hope for new therapeutic targets. Eradicating GICs would prevent the initiation of GBM on the periphery of surgical resection and reduce drug resistance and recurrence [68]. Three strategies are currently being studied to induce apoptosis of GICs: (i) directly targeting the signaling pathways involved in the self-renewal of GICs (Table 1), (ii) inducing their differentiation to sensitize them to therapies, and (iii) inhibiting the pathways that control their resistance.

#### 3.1.1. Targeting the Self-Renewal of GICs

(i) Wnt pathway

The Wnt signaling pathway is involved in the development of neural stem cells [72]. Aberrant activation of this pathway is involved in their malignant transformation and the development of brain tumors [73]. The Wnt pathway is also involved in the invasion of GBMs and in the epithelial-mesenchymal transition. Inhibiting the Wnt pathway in GICs leads to the sensitization to TMZ by decreasing the transcription of the transport proteins ABCC2 (MRP2) and ABCC4 (MRP4) [74]. Two proteins are being investigated in the inhibition of the Wnt pathway: β-catenin and GSK3-β. Diclofenac and Celecoxib, non-steroidal anti-inflammatory drugs, respectively, have been shown to inhibit β-catenin and to induce a decrease in the proliferation and migration of GBMs cells [75]. Tested in Phase II in newly diagnosed GBMs, combined with TMZ, Celecoxib had no survival benefit (NCT00112502) [69]. Two GSK3-β inhibitors were assayed in preclinical assays on GBMs cells: AR-A01441 and LiCl. These two agents increase the apoptosis of GBMs cells, decrease neurosphere formation and clonogenicity [76]. Two new selective inhibitors of the Wnt pathway have been synthesized: SEN461 and XAV939 [77]. In vitro, SEN461 is known to be responsible for the inhibition of GBM cell growth. However no clinical trials have analyzed the efficacy of GSK3-β inhibition [78] in vivo.

(ii) Notch pathway

The Notch pathway is involved in invasion, resistance to anti-VEGF (Vascular endothelial growth factor) therapies and recurrences of GBMs [79,80,81]. Activation of this pathway induced by one of its ligands (Delta and Jagged) results in the cleavage of the Notch receptor, allowing the release of the receptor’s intracellular domain and its translocation to the nucleus. Notch’s cleavage is mediated by α and γ-secretase [82]. It has been suggested that targeting the Notch pathway via inhibition of γ-secretase [83,84] may be useful. Several inhibitors have been tested in vitro, such as MRK003 [85], GSI (RO4929097) [86], and dnMAML [87]. Only the GSI compound (R04929097) is currently being tested in clinics (Table 1). A Phase I study, investigating the toxicity of GSI combined with Bevacizumab, showed encouraging results (NCT01189240). The study is being pursued in a Phase II study [88].

(iii) Hedgehog (SHH) pathway

The SHH signaling pathway is associated with resistance to radiotherapy and chemotherapy. Two main effectors of this pathway exist: SMO (smoothened) and Gli1 (glioma-associated oncogene homolog 1) [89,90,91]. SMO inhibition is achievable via two inhibitors, LDE225/Sonidegib and GDC-0449/Vismodegib [92]. The latter is currently in clinical trials (Phase II) in recurrent GBMs (NCT00980343) and (Phase I/II) in patients with newly diagnosed GBM without O6 methylguanine methyl transferase (MGMT) promoter methylation (NCT03158389, referred below as N^2^M^2^ (NOA-20), NCT Neuro Master Match the umbrella protocol for Phase I/IIa trials of molecularly matched targeted therapies combined with RT) [71]. 

Glasdegib (PF-04449913), another SMO inhibitor that has demonstrated potent and selective inhibition of Hedgehog signaling in vitro, and significant antitumor efficacy in vivo in various solid and hematologic malignancies [93], is a rational therapeutic agent currently in phase I/II for patients with newly diagnosed GBM, since it inhibits SHH pathway interfering with cancer stem cells and endothelial migration.

Gli1 can be inhibited by the cyclopamine. This steroidal alkaloid induces a decrease in the number of GICs and leads to RT sensitization [94]. The optimization of cyclopamine, by addition of a glucuronide group, showed a decrease in the tumor mass without having the toxic effects of Gli1 inhibition in astrocytes. This formulation specifically targets tumor cells expressing the beta-glucuronidase enzyme [95]. Similarly, the formulation of cyclopamine in micelles leads to inhibition of the proliferation and invasion of GBMs cells. This formulation also enhances the cytotoxic effect of TMZ in vivo [96]. No clinical studies have tested Gli1 inhibition.

(iv) STAT3 pathway

The transcription factor STAT3 has an established function in neural stem cell and astrocyte development. It has been found to play dual tumor suppressive and oncogenic roles in glial malignancy depending on the mutational profile of the tumor [97]. Napabucasin (BBI608), a small molecule that blocks stem cell activity in cancer cells by targeting the STAT3 pathway, is currently in clinical Phase I/II in combination with TMZ in adult patients with recurrent or progressed GBM (NCT02315534, Table 1).

#### 3.1.2. Inducing the Differentiation of GICs or Inhibiting Pathways That Control Resistance

Very few clinical trials addressing these points are currently developed although new targets are suggested through preclinical explorations.

As previously mentioned, inducing differentiation of GICs would sensitize them to current therapies. Simulating the BMP (Bone Morphogenetic Proteins) pathway is possible by different mechanisms:

- Activation of an effector of the BMP pathway, such as BMP-7, blocks the tumor progression in vitro [98].
- Using mimic effectors of the BMP pathway: the BMP-2 protein mimicking peptide, GBMP1, has been developed to activate this pathway and is currently being studied [99]. Activation of the BMP pathway is currently tested in clinical trials. A Phase I study is testing the recombinant protein hrBMP4 in recurrent GBMs (NCT02869243).

A new strategy aims to target adenosine, which is involved in GIC chemoresistance [100,101]. Physiologically, adenosine is produced by the degradation of AMP by the factors CD39 and CD73. In GBMs cells, CD73 expression is increased and leads to an increase in adenosine levels [102]. An increase in the A3AR adenosine receptor has also been observed in GBMs cells. Inhibition of A3AR receptor expression induces a decrease in MRP1 activity and increased sensitivity to chemotherapy [102,103]. CD73/A3AR/MRP1 is a potential therapeutic target, not yet tested in a clinical setting.

Two other adenosine receptors, A1B and A2B, are involved in apoptosis and GIC differentiation. The stimulation of these receptors by agonists helps to sensitize GICs to chemotherapy [104].

### 3.2. Targeting Growth Autonomy and Migration

Mutations in RAS/MAPK and PI3K/AKT pathways are reported in 88% of GBMs [105]. Their hyperactivation plays a central role in cell survival, growth, angiogenesis and cellular metabolism. It is mainly caused by ligand-induced stimulation of tyrosine kinase receptors (RTKs), such as epidermal growth factor receptor (EGFR) and platelet-derived growth factor receptors (PDGFR). The different RTKs are activated by the autophosphorylation of their tyrosine kinase domain, which results in the binding and activation of PI3K. The activated PI3K transforms PIP2 into PIP3. The latter binds to AKT and transports it to the plasma membrane where residues are phosphorylated by PDk-1 (on Thr308) and mTORC2 (on Ser473). The activation of AKT leads to a phosphorylation cascade and to the activation of several proteins involved in cell growth, angiogenesis and apoptosis, including mTOR and its partner mTORC1. One of the main inhibitors of this pathway is PTEN, which prevents the transformation of PIP2 into PIP3 [106].

The RAS/MAPK pathway activation results in the transformation of GDP to GTP, recruitment of RAF to the membrane and its activation, and ERK phosphorylation.

Targeting the different effectors of these pathways would reduce growth autonomy and migration of the GBM.

#### 3.2.1. Inhibition of EGFR and HER2

The ErbB family of proteins contains four receptor tyrosine kinases, structurally related to the epidermal growth factor receptor (EGFR or HER1). EGFR and HER2 are promising anti-tumor targets for the therapy of GBM (Table 2).

##### i. Inhibition of EGFR

The EGFR signaling drives cancer development. EGFR aberrant expression and signaling promotes cell growth, survival, invasion and angiogenesis, and regulates tumor metabolism and cell stemness [107]. EGFR is a clinical target in solid tumors. In GBM, EGFR is amplified and/or mutated in more than 50% of cases [108]. EGFR and its mutant EGFRvIII are the subjects of extensive research. Several strategies are proposed to inhibit these receptors, including monoclonal antibodies, tyrosine kinase inhibitors (TKI) and anti-tumor vaccines. The first two classes are described in this review (Table 2).

##### Monoclonal Antibodies

Cetuximab was the first chimeric antibody proposed to target EGFR. Two Phase II studies did not show any therapeutic benefit in patients with recurrent GBM, either as monotherapy [128] or in combination with Bevacizumab and Irinotecan [110].

Panitumumab, the first fully human monoclonal anti-EGFR antibody to enter clinical trials for the treatment of solid tumors, did not prove to be beneficial for GBM patients in a phase II with irinotecan (NCT01017653). 

Nimotuzumab, a humanized anti-EGFR antibody, also did not show a gain in overall survival (OS) or progression-free survival (PFS) in patients newly diagnosed and treated with the Stupp protocol (phase III) (NCT00753246) [111]. These results were disappointing compared to an earlier study that showed that the combination of nimotuzumab with RT resulted in prolonged survival [129]. Nimotuzumab remains a potential interesting therapy. Indeed, an enhancement of the cytotoxic activity of TMZ in vivo has recently been observed [130].

GC1118, an anti-EGFR antibody which seems more potent to inhibit EGF binding to EGFR than cetuximab or panitumumab [131] is currently being tested as monotherapy (NCT03618667).

Sym004 is a synergistic antibody combination containing two recombinant mAbs (futuximab and modotuximab) which binds to different non-overlapping epitopes of EGFR and promotes a rapid EGFR internalization and degradation. Sym004 overcame cetuximab resistance in pre-clinical lung cancer cells [132]. However, it did not improve OS in patients with metastatic colorectal cancer [133]. In GBM, it is evaluated as monotherapy (NCT02540161).

Depatuxizumab-mafodotin (ABT-414) is an antibody-drug conjugate (ADC) composed by an anti-EGFR IgG conjugated to the tubulin inhibitor monomethyl auristatin F [134]. Depatuximab-mafodotin failed to show survival benefit in newly diagnosed GBM but used in combination with TMZ in EGFR amplified recurrent GBM presented a possible efficiency [135].

##### Tyrosine Kinase Activity Inhibitors

Erlotinib is a reversible inhibitor of EGFR tyrosine kinase activity. Two Phase II studies did not show any improvement in OS when combining erlotinib and bevacizumab with TMZ as adjuvant therapy to the Stupp protocol in newly diagnosed patients [114,136]. Similar results were observed in a Phase II study analyzing the efficacy of Erlotinib in combination with sorafenib [113].

Gefitinib is a reversible and specific inhibitor of EGFR tyrosine kinase activity. Combined with RT in newly diagnosed patients, OS is not improved compared to RT alone [124], nor is it improved as adjuvant after RT [137].

Afatinib, an irreversible pan-inhibitor of the ErbB family (including EGFR and EGFRvIII) did not show better results than TMZ in a Phase II study (NCT00727506). Nevertheless, an increase in PFS has been observed in patients with tumors expressing EGFRvIII or with EGFR amplification [125].

Dacomitinib is a pan-HER family inhibitor (EGFR, HER2, and HER4), approved as first-line treatment of EGFR mutant NSCLC. In GBM, dacomitinib was tested as monotherapy in tumors with EGFR amplification or with the presence of the most common EGFR mutation in GBM EGFRvIII, but it provided minimal benefits [126].

##### ii. Inhibition of HER2

HER2 tends to be activated by forming heterodimers with other members of the family or other receptors, since no activating-ligand is known [138]. HER2 overexpression in breast cancer cells promotes tumor aggressiveness and thus became a therapeutic target combined with a companion test [139]. HER2-targeted antibody trastuzumab in breast cancer is a successful example of a targeted therapy.

Even though HER2 expression is low in GBM cells, multitargeted TKI of HER2, EGFR and VEGFR family are being tested in clinical trials. 

Lapatinib and neratinib are two treatments used in HER2-positive breast cancer. In GBM, Lapatinib, a dual EGFR and HER2 kinase inhibitor, did not provide therapeutic gain in patients with recurrent GBMs in a Phase II study [140]. This compound together with TMZ and RT in newly diagnosed patients is in clinical trials (NCT01591577) [141].

#### 3.2.2. Multikinase Inhibitors

Series of multikinase inhibitors have been tested in GBM (Table 3 and Table 4). Usually developed initially against one specific target, they proved able to inhibit different RTKs or non-receptor kinases as their ATP/ADP binding pocket revealed similarities. This characteristic may have advantages as simultaneously inhibiting several kinases may limit drug resistance and compensatory pathways [142]. Most of them are able to target EGFR, PDGFR, vascular endothelial growth factor receptors (VEGFR) known targets of GBM or even HER2, a target in breast cancers.

Anlotinib inhibits VEGFR, FGFR, PDGFR and c-kit [143]. Anlotinib is tested in GBM clinical trials as monotherapy or combined with Stupp protocol.

TG02 is an inhibitor of CDKs, JAK2 and FLT3 able to penetrate the blood-brain barrier and is therefore an interesting therapeutic for brain tumors [144]. TG02 is assayed in GBM in combination with TMZ (NCT02942264).

Tesevatinib is an inhibitor of EGFR, HER2, VEGFR and ephrin B4 [145], used in polycystic kidney disease and tested as monotherapy in GBM (NCT02844439).

Vandetanib, an inhibitor of EGFR, VEGFR2 and RET, has shown encouraging preclinical results. A 94% decrease in xenograft tumor size was observed when combined with TMZ and compared to TMZ alone [146]. However, the addition of vandetanib to the Stupp protocol does not prolong the survival of newly diagnosed patients (NCT00441142) [147].

Other multi-kinase inhibitors, such as cabozantinib, TG02, bosutinib are tested in GBM. All clinical trials, ongoing or completed, are listed in Table 3.

(i) Inhibition of PDGFR

Similar to EGFR, the PDGF receptor is involved in the activation of the PI3K pathway. It is overexpressed or amplified in 75% of GBMs and thus appears as an interesting therapeutic target [150]. PDGFR inhibition has been largely explored in GBM. However, no specific PDGFR inhibitor exists and inhibitors are multikinase inhibitors (Table 4). 

Imatinib was the first inhibitor targeting PDGFRα/β, BCR-Abl, c-kit. Although Imatinib has not shown clinical benefit in combination with hydroxyurea [151], it is currently in clinical trials.

Dasatinib, an inhibitor of PDGFRβ, EPHA2, BCR-Abl, c-kit and SRC, was ineffective in a Phase II study in patients with recurrent GBMs [152].

Tandutinib, an inhibitor of PDGFRβ, FLT3, c-Kit, was tested in a Phase II study with Bevacizumab in patients with recurrent GBMs. The results indicated that this combination does not improve patient survival compared to standard therapy (NCT00667394) [153]. Another Phase II study showed similar results and was stopped [154].

Sunitinib, an inhibitor of PDGFRα/β, c-kit, VEGFR1/2/3, FLT3 and RET, also provided disappointing results. A Phase II study did not show any clinical benefit of sunitinib in patients with recurrent GBMs compared to bevacizumab or conventional chemotherapies [166]. Similar results were observed in newly diagnosed non-operable patients [160].

Regorafenib inhibits a mutant isoform of BRAF (BRAFV600E), KIT, RET, angiopoietin 1 receptor, PDGFRα, VEGFR1/2/3 and FGFR1/2 [167]. In GBM, it is evaluated as monotherapy or together with the Stupp protocol.

Crenolanib, an inhibitor PDGFR and FLT3 is evaluated as monotherapy in recurrent GBM with PDGFRα gene amplification (NCT02626364).

Ponatinib (AP24534), a multi-targeted kinase inhibitor of BCR-Abl, PDGFRα, VEGFR2, FGFR1, and Src [168] but also RET, KIT, and FLT1, is assayed as a monotherapy in recurrent GBM refractory to bevacizumab (NCT02478164).

Leflunomide, an antimetabolite and inhibitor of PDGFR, EGFR and FGFR, is used for the treatment of rheumatoid arthritis. In preclinical trials, the active compound inhibited glioma cell proliferation in vitro and in vivo. Now it is evaluated as monotherapy in GBM (NCT00003293).

Besides these multi-target drugs, specific anti PDGFR antibodies have been designed and tested in GBM. A fully human anti-PDGFR antibody (IMC-3G3) blocks ligand binding and receptor activation and is being tested in different solid tumors [169]. A comparative clinical trial between IMC-3G3 monotherapy and ramucirumab (targeting VEGFR2) monotherapy did not show improved survival (NCT00895180).

Another monoclonal anti-PDGFRα antibody, MEDI-575, was well tolerated but showed limited clinical activity in GBM [163].

(ii) Inhibition of IGFR1 and FGFR

Insulin-like growth factor 1 receptor (IGF1R) activation by its ligand IGF1 promotes GBM cells survival through PI3K/AKT pathway activation. Thus, inhibition of IGF1R may be an interesting strategy to suppress GBM progression [170]. Moreover, IGF1R overexpression in GBM is correlated with a shorter survival and lack of response to TMZ [171]. A phase I/II clinical trial (NCT01721577, Table 4), used AXL1717, an antagonist of IGF1R, as a single agent in the treatment of recurrent malignant astrocytomas. Monotherapy was well tolerated. Further optimizations in dose need to be performed [165].

Mutations of fibroblast growth factor receptor (FGFR) are rare in GBM but signalling through FGFRs impacts GBM progression and patient survival [172]. For example, fusion between FGFR and TACC (transforming acidic coiled-coil containing proteins) enhances tumor-growth and aneuploidy events [173]. FGFR1,2,3 mutations and fusion are targeted by BGJ398 (Table 4) as monotherapy in a phase-II clinical trial in GBM. However, BGJ398 was out licensed and no more studies were performed.

(iii) Inhibition ALK

Anaplastic lymphoma kinase (ALK), a transmembrane receptor *tyrosine* kinase that belongs to the insulin receptor superfamily, is expressed in about 60% of GBMs and conveys tumorigenic functions. Second-generation ALK inhibitors, such as alectinib, might be novel therapeutic agents against GBMs, as they induced cell death in various human GBM cell lines with lower concentrations than other ALK inhibitors. The specific anti-tumor mechanism of alectinib is not yet described [174]. Alectinib is currently tested in the N^2^M^2^ Phase I/IIa clinical trial (NCT 03158389, Table 4) [71].

#### 3.2.3. Inhibition of the PI3K/AKT Pathway 

Table 5 describes the clinical trials concerning the inhibition of the PI3K/AKT pathway.

(i) Inhibition of mTOR.

Another target in the PI3K/AKT pathway is mTOR. Several mTOR inhibitors are available and tested in clinical trials.

Among them, temsirolimus, which has recently been shown to target GICs [187], is the subject of many clinical trials. Two Phase II studies did not show clinical benefits when combined with bevacizumab [175] or sorafenib (NCT00800917) [188]. More recently, a Phase II study comparing the combination of temsirolimus with RT in newly diagnosed patients did not show any difference in survival compared to the Stupp protocol (NCT01019434) [178]. It is actually tested in the N^2^M^2^ (NOA-20) clinical trial (NCT03158389) [71].

Sirolimus (rapamycin) showed promising preclinical results by decreasing 95% tumor mass in vivo [189]. In addition, it also decreased the proliferation of GICs [190] and their differentiation [191]. Despite these results, sirolimus combined with erlotinib is not effective in GBM recurrence (NCT00672243, Table 2) [116].

Similar results were observed with everolimus. A Phase II study showed that the administration of everolimus before the Stupp protocol in newly diagnosed patients does not provide any clinical benefit compared to the standard protocol [181].

ABI-009 is a novel albumin-bound mTOR inhibitor (albumin-bound rapamycin nanoparticles, nab-rapamycin), currently tested as single agent or in combination with standard therapies (NCT03463265) in a Phase II study.

AZD2014, an inhibitor of both mTORC1 and mTORC2, causes radiosensitization of GICs in vitro and in vivo [192]. This compound is currently in a Phase I clinical trial (NCT02619864).

(ii) Inhibition of PI3K

Several PI3K pan-inhibitors have shown promising in vitro and in vivo results, some of which are being tested in clinical trials.

Pictilisib is an isoform inhibitor of PI3K α/δ. Combined with RT and TMZ, it has a pro-apoptotic action, increases autophagy and decreases the migration capacities of GBMs cell lines. In vivo, it increases sensitivity to RT and TMZ [193]. Pictilisib was compared with pembrolizumab in a phase I/II study but data are not published (NCT02430363).

Buparlisib (BKM120) inhibits cell invasive capacities in vitro and reduces tumor invasion in vivo [194,195]. It is currently being tested in two phase I/II and II studies (NCT01349660 NCT01339052). In the Phase II study (NCT01339052), buparlisib achieved significant brain penetration, but had low efficacy in patients with PI3K-activated recurrent GBM, which was explained by incomplete blockade of PI3K pathway in tumor tissue [196].

Sonolisib (PX-866), an isoform inhibitor of PI3K α, δ and γ reduces the invasive and angiogenic capacities of GBM cells in vitro. In vivo, decreased tumor growth and increased survival of xenografted mice [197] were observed. A Phase II study did not show clinical benefit in the case of recurrent GBMs (NCT01259869) [183].

Paxalisib (GDC-0084) is a brain-penetrant small molecule inhibitor of the PI3K/AKT/mTOR pathway. An interim analysis from Kazia Therapeutics reviewed OS of 17.7 months (nine patients) compared to the median OS for patients treated with TMZ (12.7 months). Final data of the phase II trial (NCT03522298) are expected to be presented in the first half of 2021, but FDA has already granted fast track designation to paxalisib.

(iii) Inhibition of AKT

Enzastaurin is an inhibitor of AKT and protein kinase C. This molecule was the first to provide clinical benefit in a subgroup of patients with recurrent GBMs according to their MGMT status [185]. Enzastaurin has been compared to lomustine in a Phase III clinical trial (NCT00295815). Median PFS, 6-month PFS rate and OS did not differ significantly between enzastaurin and lomustine. Enzastaurin was well tolerated, had a better hematologic toxicity profile but did not have superior efficacy compared with lomustine in patients with recurrent GBM [184].

Other AKT inhibitors with promising results are being tested in preclinics or Phase I, such as perifosine [198], nelfinavir [199], MK2206 [200].

#### 3.2.4. Inhibition of RAS/MAPK Pathway

RAS/MAPK pathway is activated by many receptors including tyrosine kinase receptors and involved in cell survival and proliferation. RAS/MAPK has been targeted in GBM (Table 6).

One inhibitor of this pathway, TLN-4601, did not demonstrate therapeutic efficacy in monotherapy in a Phase II study in the event of recurrence [201].

Sorafenib is a Raf-1 and p38 inhibitor, involved in the RAS-MAPK, VEGFR, c-kit and PDGFR pathways [206]. Although sorafenib has been shown to potentiate the pro-apoptotic effect in GBMs cells [207], it does not appear to improve sensitivity to radiotherapy and chemotherapy in vivo [208]. For clinical trials, the combination of sorafenib and TMZ in recurrent GBMs provides a PFS of 3.2 months and an OS of 7.4 months [209]. Combined with bevacizumab [204], erlotinib [114] and temsirolimus [188], it does not provide clinical benefit. Disappointing results were also observed in newly diagnosed patients treated with sorafenib and combined to the Stupp protocol in adjuvant therapy [202].

Two Ras-MAPK inhibitors are in Phase II clinical trials: LY2228820 and atorvastatin. The latter molecule could potentiate the effects of TMZ in vitro and in vivo [210]. In a Phase II study (NCT02029573) in combination with standard therapy (RT/TMZ) in newly diagnosed GBM patients, preliminary results are encouraging and met criteria for continued accrual [205].

Dabrafenib is a BRAF inhibitor that binds and inhibits the active conformation of the receptor. Dabrafenib is evaluated in combination with the MEK inhibitor trametinib in newly GBM (NCT03919071).

A very recent study includes binimetinib (a MEK inhibitor) with encorafenib (a BRAF inhibitor) in adults with recurrent BRAF V600-Mutated HGG (NCT03973918).

The lipid proliferation switch led to the discovery of a novel anticancer drug target, the tumor repressor protein sphingomyelin synthase 1 (SGMS1). The activation of SGMS1 by 2OHOA, a synthetic hydroxylated fatty acid, modulates the lipid content of cancer cell membranes, regulates the localization of key signalling proteins, including Ras and PKC at the plasma membrane, leading to inactivation of Ras/MAPK, PI3K/Akt and PKC/cyclin/CDK signalling pathways [211]. The clinical trial in Phase I/IIa NCT01792310 demonstrated its safety and efficacy in humans. 2OHOA was designed as orphan drug by the European Medicines Agency (EMA) for the treatment of glioma and is now tested in a Phase IIb study (NCT04250922).

### 3.3. Targeting the Cell Cycle and Escape to Cell Death

A major reason for the failure of chemotherapy is the resistance of GBM cells to cell death by apoptosis, necrosis or autophagy [212,213].

#### 3.3.1. Therapies Targeting Apoptosis

Apoptosis can be mediated by the extrinsic and the intrinsic pathways. The extrinsic pathway results from the activation of the TNF-R1, FAS and DR4/DR5 death receptors through their respective ligands TNFα, CD95 and TRAIL [214]. The intrinsic pathway is regulated by proteins of the BCL-2 family and of the inhibitor of aptotosis (IAP) family. Pro and anti-apoptotic members of the BCL2 family regulate mitochondria-dependent cell effects. When apoptosis is triggered mitochondria become permeable and release cytochrome C. The two pathways converge on a series of catalytic cascades involving caspases [105]. The tumor suppressor p53 is implicated in several pro-apoptotic pathways and appears mutated in about 30% of GBM. Restoring apoptosis may be obtained by targeting different apoptosis players (Table 7).

(i) Activating proteins involved in the extrinsic pathway of apoptosis

The CD95 death receptor is overexpressed in GBMs and mesenchymal GICs. It is also associated with epithelial-mesenchymal transition [219]. APO010 and APG101 are two CD95 agonists. APO010 has significant anti-tumor activity in GICs, increasing their sensitivity to TMZ in vitro. Administered locoregionally, APO010 increases mice survival [220]. A phase II study showed that the combination of the agonist APG101 with re-irradiation in recurrent GBM improves PFS but not OS compared to re-irradiation alone. This therapeutic benefit is more pronounced in mutated IDH tumors [215].

TRAIL/DR5 dependent cell death can be induced by ONC201. ONC201 binds and antagonizes dopamine receptors DRD2 and DRD3 causing p53-independent apoptosis in tumor cells. ONC201 inhibits the phosphorylation of AKT and ERK pathways, leading to the dephosphorylation of transcription factor FOXO3A, and thus transcription of pro-apoptotic death receptor ligand TRAIL. Through a stress response activation ONC201 is involved in EIF2α phosphorylation and increases DR5 expression [221,222] Based on the the first results using ONC201 in monotherapy which showed that the treatment was well tolerated and that ONC201 may have single agent activity in GBM [223], a phase II clinical trial was started on GBM with H3 K27M mutation (NCT02525692). It showed that ONC201 can be used regardless of age or location [216].

(ii) Activating proteins involved in the intrinsic pathway of apoptosis

The TSPO protein is involved in the permeabilization of the mitochondrial membrane. Its level of expression being correlated with a poor prognosis, it is considered a potential target for apoptosis restoration [224]. Several ligands of TSPO (Translocator protein), derived from pyrazolo[1,5-a]pyrimidine acetamides, are able to specifically reduce the proliferation of GBMs cells [225]. No clinical trials are underway with these new molecules.

(iii) Targeting proteins involved in the regulation of apoptosis

Due to its role in regulating both pathways of apoptosis, targeting the p53 protein has also been suggested to reactivate its pro-apoptotic functions, by gene therapy or by inhibiting its interaction with MDM2 [226,227].

In a recent study, a tumor-targeting p53 nanodelivery system (SGT53) showed sensitization of resistant GBM cells to TMZ in vitro and increase in the survival of xenografted mice [228]. Gene therapy is currently in a Phase II clinical study (NCT02340156).

Inhibition of MDM2-p53 interaction to trigger apoptosis is an approach that showed encouraging preclinical results. Among these, ISA27 inhibits cell growth in vitro and in vivo [229] while nutlin-3a induces apoptosis and senescence of glioma cells [230]. α5β1 integrin-specific inhibition in association with nutlin-3a also triggered a strong apoptosis in glioma cells expressing a functional p53 [231]. Idasanutlin (RG7388) with more potency, selectivity, and better pharmacokinetic profile than other MDM2 inhibitors appears interesting in preclinical assays, is tested in clinical trials for acute myeloid leukemia and recently in the N^2^M^2^ (NOA-20) clinical trials in GBM (NCT01358389) [71]. Finally, the AMG-232 inhibitor has shown encouraging results including inhibition of tumor growth in several xenografts (lung, osteosarcoma, etc.) and tumor regression in mouse models [232]. This agent is currently in Phase I clinical trials (NCT03107780, NCT01723020).

Farnesyltransferase inhibitors (FTI) can induce apoptosis, as they revert cells to a state in which cell-substratum attachment is necessary for viability [233]. Inhibition of farnesyltransferase (FT) by tipifarnib blocks the prenylation of the farnesyltransferase tail CAAX motif, thereby preventing Ras binding to the membrane and its activation. Tipifarnib is tested in four clinical studies in monotherapy or combined with RT or TMZ or other targeted therapies (NCT00050986, NCT00058097, NCT00005859 and NCT00335764). Lonafarnib (SCH66336) is a FTI that blocks farnesylation of cell proliferation proteins, such as RhoB, RAS, laminins and CCAX phosphatase [234,235]. It inhibits in vitro [236] and in vivo [237] cell growth in combination with chemo and/or radiotherapy. A phase II was performed in combination with TMZ (NCT00038493).

Simultaneous reactivation of p53 and TSPO proteins appears to be more effective in promoting apoptosis in GBMs cells but also in reducing the risk of resistance [238]. Reactivating these proteins using molecules with irreversible action has been suggested in order to reduce the risk of recurrence [239].

Another potential approach is to target anti-apoptotic proteins from the BCL-2 family. The compound gossypol binds to the common part of proteins Bcl-2, Bcl-XL and Mcl-1 [240]. Its combination with TMZ was shown to inhibit the invasive and proliferative abilities of GBMs cells and angiogenesis in vitro, and to cause apoptosis in vivo [241]. Gossypol was tested as monotherapy in a phase II (NCT00540722).

Finally, a new therapy targeting the Bcl-2 protein consists of the administration of spherical nucleic acid (SNA). SNA-NU-0129, a formulation containing gold nanoparticles and a siRNA targeting BCL2L12, is involved in the inhibition of this protein and in the induction of cellular apoptosis in vitro [242]. A Phase I study is ongoing in recurrent GBMs and gliosarcomas (NCT03020017).

#### 3.3.2. Therapies Targeting Autophagy

Autophagy is a degradation mechanism that can also induce cell death independently of caspases. It is based on the encapsulation of proteins, cytoplasm and organelles in vesicles that will be degraded in lysosomes. The pro- or anti-tumor function of autophagy in the GBM is still uncertain [243]. Molecules inducing autophagy, such as curcubitacin [244], itraconazole [245], rutin [246], givinostat [247] can have different consequences, but none of them are yet tested in Phase I/II or more.

In addition, chloroquine, inhibiting autophagy via lysosomal protease blockade and fusion between lysosomes and autophagosome [248], provoked a decrease in cell proliferation and migration, and an induction of apoptosis in vivo and in vitro [249]. Chloroquine is in Phase I and II clinical trials in combination with TMZ and/or RT (NCT02378532, NCT02432417, NCT00224978 & NCT00486603) (Table 8).

#### 3.3.3. Targeting Multifaceted Pathways and DNA Modifications

Table 9 details all the clinical trials of this section.

(i) CDK4/6 inhibitors

Cyclin-dependent kinases 4 and 6 (CDK4/6) signalling regulates cell cycle, cell differentiation, metabolism and apoptosis. In glioma cells, CDK4 is overexpressed which led to glioma cell proliferation and TMZ resistance [260]. CDK4/6 inhibitors (palbociclib/PD 0332991, abemaciclib) specifically blocked the cell cycle at the G1-to-S transition phase, leading to cell cycle arrest and stopped cell proliferation [261]. These inhibitors are approved in combination with anti-oestrogen therapies for the treatment of hormonal breast cancer, and are being studied in GBM upon surgical resection. Palbociclib is one of the drug tested in the GBM phase I/IIa trial NCT03158389 [71].

(ii) Proteasome inhibitors

The proteasome is a central cellular protein-degradation machinery. It regulates cell homeostasis in normal and cancer cells. Bortezomib, the first-generation proteasome inhibitor, was approved for the treatment of multiple myeloma and mantle cell lymphoma [262]. This therapy is able to increase apoptosis levels in preclinical brain tumor assays. Moreover, clinical trials using proteasome inhibitors in combination strategies are being tested to maximize therapeutic efficacy and limit toxicity [263]. Bortezomib is studied in combination with TMZ and/or radiation, or with an inhibitor of histone deacetylase.

Marizomib, is a second-generation, irreversible proteasome inhibitor with a more lipophilic structure, having the ability to cross the blood-brain barrier [264]. It has been tested in patients with newly diagnosed and recurrent GBM in phase I and phase II studies. In patients with recurrent GBM, marizomib was administered in a Phase I/II study as a single agent or in combination with bevacizumab (NCT02330562) and in a Phase II study as a single agent or in combination with bevacizumab or RT/TMZ or ABI-009, or lomustine (NCT03463265). Based on encouraging observations [265], marizomib combined with RT/TMZ is actually in a Phase III study (NCT0334509).

(iii) Histone deacetylase inhibitors

Epigenetic alterations in histones control chromatin structure and transcriptional activation. Besides their potential role in onset and progression of cancer, they are generally reversible and thus interesting therapeutic targets. Histone acetylation relaxes chromatin and allows access to DNA and transcription activation. On the other hand, histone deacetylases (HDAC) compacts chromatin and represses transcription [266]. HDACs can be essential for cancer cell survival and growth, showing an epigenetic vulnerability of tumor cells. HDAC inhibition can induce tumour cell cycle arrest, apoptosis, reduction of angiogenesis and enhancement of tumor-mediated immunity [266,267]. HDAC inhibitors [268] in GBM tends to re-establish the balance of histone acetylation and sensitizes tumor-mediated immunity. It can also sensitize tumor cells when used in combination, for example, with radiation therapy [267]. Several clinical trials are testing HDAC inhibitors as monotherapy or in combination in GBM. Vorinostat as a monotherapy had modest activity in patients and did not improve PFS or median OS in association with bevacuzimab (NCT01738646) or bortezomib (NCT00641706). Another HDAC inhibitor, FR901228 (Romidepsin), was ineffective for patients with recurrent GBM (NCT00085540).

(iv) TGF-β inhibitors

Transforming growth factor-beta (TGF-β) is a cytokine secreted by immune cells, tumor cells, and stromal cells. TGF-β is overexpressed GBM tissues but inexistent in normal brain. TGF-β signalling regulates GBM proliferation, invasion, angiogenesis, immunosuppression, and GSCs stemness [269]. Targeting TGF-β signaling mechanisms is a promising therapeutic strategy [270]. In GBM clinical trials, TGF-β pathway are targeted by antisens oligonucleotide (trabedersen, NCT004331561) and by small molecules, OKN-007 (NCT03649464) [271], and galunisertib (NCT01582269, NCT01220271). Results are available for galunisertib and trabedersen.

Targeting of TGF-β2 signaling through inhibition of TGF-β mRNA translation by using the antisense oligonucleotides trabedersen, injected in the resection cavity, was tested in GBM in a Phase IIb (NCT00431561) but the first results did not show statistically significant differences among the three arms: trabedersen at doses of 10 or 80 mM or standard chemotherapy (TMZ or procarbazine/lomustine/vincristine) [256].

Galunisertib targets the TGF-β1 receptor and selectively inhibits the serine/threonine activity of the receptor, thereby preventing the phosphorylation of downstream proteins, SMAD2 and SMAD3. It demonstrated antitumor effects in preclinical and radiographic responses [272]. But no differences in efficacy, safety or pharmacokinetic variables were observed in a Phase Ib/IIa clinical trial (NCT01220271) between the two treatment arms (TMZ/RT with and without galunisertib) [257].

(v) PARP inhibitors

Defects in DNA repair pathways are a characteristic feature of cancer cells. They participate in tumour development by promoting genomic instability. For more than 50 years, this characteristic has been exploited as a therapeutic opportunity for the treatment of cancer, with the use of conventional cytotoxic chemotherapies. More recently, the discovery of a synthetic lethality interaction between DNA damage induced by PARP (poly[ADP-ribose] polymerase) inhibitors led to the development of new therapeutic approaches. The PARP proteins use NAD^+^ as their substrate to modify acceptor proteins with ADP-ribose modifications. Most PARP inhibitors target the NAD^+^ binding site.

A high expression of PARP-1 mRNA is associated with low survival, particularly in classical GBMs [273]. A few molecules inhibiting PARP-1 are in clinical trials. Among them, iniparib (BSI-201) taken concomitantly with RT and TMZ has shown encouraging results, in human glioma xenografts, resulting in complete tumor regression in 70% of animals [274]. This PARP1 inhibitor plus TMZ was evaluated in a phase I/II in newly-diagnosed GBM (NCT00687765). Other NAD^+^ mimetics, olaparib (AZD2281), veliparib (ABT-888) and pamiparib (BGB-290) inhibit the catalytic activity of PARP-1 and PARP-2 and are currently being studied in phase I or I/II clinical trials. Only results for veliparib combined with TMZ (NCT01026493) are available [259]. The concept of this study was to exploit methylation at positions N3-adenine and N7-guanine, supposedly independent of the MGMT effect and related more to base excision repair with PARP. But the study did not demonstrate any clinical activity.

### 3.4. Targeting Angiogenesis

Angiogenesis is a complex process regulated by multiple signaling pathways. Due to a high tumor proliferation, access to oxygen and nutrients decreases in some areas of a tumor, leading to hypoxia and necrosis. GBM are highly angiogenic tumors and blocking neo-angiogenesis has represented an interesting therapeutic way for twenty years.

#### 3.4.1. Targeting VEGF/VEGFR Pathway

Clinical trials for VEGF and VEGFR targeting are described in Table 10.

(i) Bevacizumab

VEGF is overexpressed in GBMs and plays a major role in angiogenesis by activating its receptor VEGFR [275]. Since 2009, the food and drug administration (FDA) has approved bevacizumab, an anti-VEGF antibody, as a treatment in recurrent GBMs. Indeed, non placebo-controlled Phase II clinical trials highlighted the bevacizumab anti-tumor activity and this molecule is considered effective alone or in combination with Irinotecan, a topoisomerase I DNA inhibitor [276,277]. Based on encouraging results, few clinical trials were conducted to evaluate the efficacy of bevacizumab in comparative studies. However, results of these trials have been estimated insufficient by EMA to approve bevacizumab use in GBM in Europe. This discrepancy between drug authorities lead to huge off-label use of bevacizumab for GBM, mostly at recurrence, since this antibody is also marketed for the treatment of ovarian, lung, breast and colorectal cancer.

For other studies presented in Table 10, bevacizumab is usually the reference treatment of the control arm to be compared to combinations of bevacizumab plus other experimental molecules targeting different pathways.

##### Clinical Trials in Recurrent GBMs

In a Phase II study, the combination of bevacizumab and TMZ did not show a survival benefit compared to bevacizumab alone [294]. Similar results were observed in several other Phase II studies with bevacizumab in combination with temsirolimus [175], Carboplatin and irinotecan [306]. Only the combination of bevacizumab and lomustine appears to provide encouraging results in terms of survival and quality of life in a Phase II study [307,308]. However, these promising results were not demonstrated in a Phase III study, in which the combination therapy resulted in a PFS benefit but no OS improvement (NCT01290939) [292].

The efficacy of bevacizumab was also studied retrospectively in patients exposed to a second irradiation [309]. This study shows that bevacizumab might be a protective agent against a second irradiation. The improvement in irradiation with an anti-angiogenic agent was explained by the normality of vascularization during VEGFR blockade. Indeed, this “normalization window” allows a temporary increase in tumor oxygenation, which improves the damage induced by irradiation [310].

##### Clinical Trials in Newly Diagnosed GBMs

No benefit for bevacizumab with or without conventional treatment was obtained in different clinical trials [284,287,297,311,312]. Only one Phase II study, analyzing the combination of RT and bevacizumab followed by an adjuvant therapy combining bevacizumab and irinotecan, showed an improvement in PFS compared to the Stupp protocol in patients with non-methylated MGMT status [291]. A (non-significant) tendency towards an OS gain was also shown when TMZ was combined with bevacizumab in neo-adjuvant Stupp protocol therapy compared to the same protocol without Bevacizumab in non-operable patients [285]. Finally, it was retrospectively shown that proneural GBMs could benefit on the addition of bevacizumab compared to placebo (OS = 17.1 vs. 12.8 months HR = 0.43; *p* = 0.002) [313].

(ii) Molecules targeting VEGFR

Pazopanib, a VEGFR1/2/3, PDGFR-α/β, and c-Kit inhibitor, administered as monotherapy, did not show therapeutic benefit in recurrent GBMs [298].

Cediranib is an oral, highly potent VEGFR inhibitor with similar activity against all three VEGF receptors and c-Kit and partial activity against PDGF receptors [314]. Cediranib, as monotherapy, has provided encouraging results in recurrent GBMs [302]. However, in combination with lomustine, cediranib did not show any therapeutic benefit, due to an increase in EGFR levels. Recently, a survival benefit has been reported with the combination of cediranib and gefitinib in recurrent GBMs [299].

Nintedanib, alone, did not show any survival benefit in recurrent GBMs [302]. Note that nintedanib is an inhibitor of VEGFR1/2/3, FGFR1/2/3 and PDGFRα/β.

Dovitinib, an FGFR, PDGFRβ, VEGFR and c-kit inhibitor, currently in clinical trials, sensitizes GBMs cells to TMZ in vitro [315,316].

Vatalanib is a VEGFR1/2/3, PDGFRβ and c-kit inhibitor. Its tolerance and safety were evaluated in a Phase I/II study (NCT00128700) in newly diagnosed patients [304] and in combination with imatinib and hyroxyurea in patients with glioma [317].

Most of these molecules have multiple targets. A few other molecules for which only a few clinical trials are ongoing and for which few results have been published, are listed in Table 10, such as tivozanib, axitinib, semaxanib, CT-322 (a molecule based on an engineered variant of the tenth type III domain of human fibronectin), and the monoclonal antibody tanibirumab (a specific binder to VEGFR2, thereby preventing the binding of its ligand VEGF).

#### 3.4.2. The secondary Pathways of Angiogenesis

Table 11 shows the clinical trials concerning the secondary pathways of angiogenesis.

The failure of anti-VEGF therapies might be explained by compensatory mechanisms, through activation of other factors involved in angiogenesis in response to VEGF inhibition.

(i) c-MET pathway

The c-MET pathway is deregulated because of an overexpression of (i) the c-MET receptor via mutation or amplification, or (ii) its HGF ligand [322,323]. Activation of this pathway is particularly important in the transformation of endothelial cells into mesenchymal cells, in the induction of aberrant vascularization and in tumor progression [324]. In addition, its activation is associated with a decrease in VEGFR2 expression, which leads to resistance to anti-VEGF therapies [325,326].

Onartuzumab, a monoclonal antibody targeting c-MET, induced a decrease in the growth of GBMs cells. Combined with bevacizumab in recurrent GBMs, ornatuzumab provides a PFS similar to bevacizumab alone. Nevertheless, this study showed a survival benefit in patients with high HGF expression or non-methylated MGMT status [318].

Other c-MET inhibitors have been developed and are currently being investigated. Among these, crizotinib (a c-MET and ALK inhibitor) causes GBMs cells to become sensitive to TMZ [327]. Crizotinib is currently being tested in combination with TMZ in a Phase I study (NCT02270034). Cabozantinib, a c-MET and VEGFR2 inhibitor, was tested in a Phase I study, combined with TMZ during the Stupp protocol [328] and in two Phase II studies as monotherapy in recurrent GBM (NCT01068782 and NCT00704288).

Targeting the c-MET ligand, HGF, is also being investigated. The anti-HGF antibody, rilotumumumab (AMG 102), did not show therapeutic benefit in monotherapy in a Phase II study in patients with recurrent GBMs [320].

(ii) PIGF pathway

Another factor involved in angiogenesis is PIGF, a member of the VEGF family, binding to VEGFR1 (FLT1) and its neuropilin-1/2 co-receptors (NRP1/2). It is expressed in GBMs and tumor endothelial cells [329]. Aflibercept, also called VEGF-trap, is a recombinant fusion protein mimicking binding domain of VEGFR1 and VEGFR2 and blocking different ligands (VEGF-A, VEGF-B and PlGF). In monotherapy or in combination with bevacizumab in recurrent GBMs, no survival benefit was observed [321,330]. These disappointing results might be explained by a decrease in PIGF expression during tumor progression, in particular after treatment with TMZ. This new therapeutic option seems more relevant in newly diagnosed patients [331].

(iii) Endoglin

Endoglin (CD105) is strongly expressed in endothelial cells with high proliferation rates [332]. TCR105 is a chimeric antibody targeting endoglin, which enhances the effects of bevacizumab in vivo, tested in two clinical trials (NCT01648348, NCT01564914). The combination of TRC105 and bevacizumab was well tolerated [333], but TRC105 with bevacizumab did not prolong median PFS versus bevacizumab alone in recurrent GBM patients [334].

Endoglin is also studied as a diagnostic marker and to estimate the degree of angiogenesis. The endoglin labelling is more typical of neoplastic endothelial cells and is correlated to Ki67, thus making it specific and sensitive to the evolution of angiogenesis in GBM [335].

#### 3.4.3. Other Pathways of Angiogenesis

Other pathways of angiogenesis are described in Table 12.

Thalidomide is a long-established anti-angiogenic agent that inhibits the angiogenic activity of β-FGF and TNF-α [345]. However, when combined with RT in GBM, no benefit was observed in newly diagnosed GBMs [346]. It has shown limited gastrointestinal toxicity and anti-tumor activity in combination with irinotecan [337], and is currently in clinical trials in combination with the Stupp protocol in newly diagnosed GBMs (NCT00047294).

Integrins αvβ3 and αvβ5 have been proposed as targets of new anti-angiogenic therapies. Promising results have been observed when combining an inhibitor of these integrins, cilengitide, with the Stupp protocol in newly diagnosed patients [342,347]. Nevertheless, in two clinical studies (one phase II and one phase III), this combination did not show survival gains in patients with methylated [340] and non-methylated [341] MGMT status. ATN161 (Ac-PHSCN-NH_2_) is a selective antagonist for α5β1 integrin. It is a capped five amino-acid peptide derived from the synergy site of fibronectin, a region which enhances the fibronectin’s RGD-mediated binding to the α5β1 integrin. ATN 161 is antiangiogenic and antimetastatic [348] and was evaluated in a phase I/II trial for recurrent malignant glioma (NCT00352313).

Trebananib (AMG-386) is an angiopoietin neutralizing peptibody comprising a peptide with angiopoietin-binding properties that is fused to the Fc region of an antibody with an antiangiogenic effect in solid tumor. It inhibits the interaction between the ligands angiopoietin-1 and angiopoietin-2 with the Tie-2 receptor [349]. Angiopoietins (Ang1 and Ang2) and their RTK (TIE1 and TIE2) are key mediators of tumor angiogenesis. Angiopoietins are overexpressed in GBM and are involved in GBM tumor growth. Moreover, angiopoietin-2 increased in bevacizumab-treated GBM and thus VEGF and angiopoietin-2 combined therapy may overcome bevacizumab resistance. A phase II study used trebananib as monotherapy on patients with recurrent GBM (NCT01290263). Trebananib was also tested in combination with bevacizumab (NCT01609790). However, combination did not significantly improve outcome over bevacizumab alone. Moreover, angiopoietin recombinant humanized monoclonal antibody, PF-04856884, was enrolled on a phase II as monotherapy in patients with recurrent GBM (NCT01225510). This study, which was withdrawn, was not listed in Tables. Until now no further trials were performed in GBM.

Endostatin is a fragment of type XVIII collagen, and one inhibitor of angiogenesis. Endostatin competitively binds to VEGFR-2 and inhibits MAPK signaling pathway and angiogenesis [350]. Recombinant human endostatin improved chemotherapy efficiency in NSCLC, breast cancer and melanoma [351,352,353]. Endostatin is actually tested in GBM in a phase II study with TMZ and irinotecan (NCT04267978).

Prostate-specific membrane antigen (PSMA) expression has been demonstrated in the tumor neovasculature of GBM, by immunohistochemical staining [354]. Although its significance has not been fully determined, PSMA may play a functional role in angiogenesis [355]. It is anchored to the cell membrane, which makes it an ideal promising therapeutic target, and can be internalized making it an appropriate candidate for pro-drug activity. Strong reactivity to the antibody component of PSMA antibody-drug conjugate (ADC), BrUOG 263, was observed in the endothelial cells of new tumor blood vessels in GBM. Following binding and internalization of PSMA ADC, the cytotoxic component of PSMA ADC will be released and destroy the neovasculature that supports tumor growth.

Matrix metalloproteinases (MMPs), especially MMP2 & 9, are thought to play a central role in invasion, owing to their ability to degrade the majority of brain ECM components [356]. Prinomastat and COL-3 are two drugs targeting MMPs that may stop the growth of GBM by stopping blood flow to the tumor. They have been tested in two clinical trials. Prinomastat/TMZ compared to TMZ alone did neither improve the one-year survival rate nor PFS (NCT00004200). The clinical trial (NCT00004147) with COL-3 in progressive and recurrent high-grade gliomas did not warrant further studies and did not reach phase II [357].

## 4. Discussion-Guidance towards Future GBM Targeted Therapies

Out of 257 Phase I/II to III clinical trials on targeted therapies listed in the tables of this manuscript, almost 70% are phase II studies (62 Phase I/II, 177 Phase II, 4 Phase II/III, 14 Phase III). Of the studies for which results are available, only 37 are comparative studies with statistical data. Comparative trials with a significant difference between two treatments are highlighted in color in the tables, in green and red for those showing a significant and non-significant difference between two treatments, respectively. It is clear that the red color dominates over the green one. Only 12 studies showed improvements mainly of PFS. Most of them (11 out of 12) involve therapies targeting VEGF and VEGFR. Although some specific explanations may be proposed for the high degree of these clinical trial failures (see below), improved clinical trial design is also needed. For exemple, Phase II trials may contain a control arm to assess the efficacy of new therapies and to reduce false positive results which remains difficult to establish in the case of recurrent disease in absence of standard treatment; historical control data became obsolete due to the improvement of patient standard of care in the clinic [358,359]. GBM is a rare disease and enrollment of patients in trials remains too low, promotion of participation must be planned to increase the number of high-quality trials [360]. In addition, the need for stratification of patients at least based on prognostic and predictive biomarkers such as the level of the predictive target is critical. Biomarkers might also help to reduce the development costs through better patient selection. A recent study on the impact of biomarker use in clinical trials shows an overall 5-fold benefit over non-biomarker use by analyzing a collection of 10,000 clinical trials for 745 drugs in four major cancer types (colorectal, lung, melanoma and breast cancer) [361]. The neuro-oncology community must work together to be able to change favorably the guidelines on the treatment of GBM [362].

Many different targeted therapeutic options are investigated. For more recent trials, we identified two main tendencies. First, is underway a clear upward trend towards approaches with multi-kinase inhibitors (i.e., when a kinase inhibitor interacts with multiple members of the protein kinase family). The second trend is towards a multi-targeted therapeutic approach. Drugs able to target multiple critical nodes for GBM development and progression might help to counteract the lack of efficiency and the rapid acquisition of resistance observed with monotherapies [363].

Several factors can explain the therapeutic failure of GBM targeted treatments:

(i) Performing a full surgical resection is impossible. Eliminating tumor cells that have migrated into the healthy parenchyma without causing neurological or cognitive disorders is not feasible. 35% of newly diagnosed patients are estimated to be non-operable due to the location or size of the tumor. In these cases, a biopsy is recommended in order to establish a diagnosis [364]. When surgery is possible, macroscopic resection is described as a good prognostic factor [365]. A recent meta-analysis showed that out of 27,865 patients diagnosed with GBM between 2004 and 2013, a biopsy (non-operable case), partial resection and massive resection accounted for 28.5%, 34.8% and 36.8% of cases [366].
(ii) Crossing the BBB is not a turnkey operation, despite its potential destruction by tumor invasion or RT. New approaches proposed, such as nanoparticles or convection-enhanced delivery (CED), [367,368], show encouraging pre-clinical and clinical results.
(iii) New molecular and genomic data has highlighted the inter- but also intra-tumoral heterogeneity of GBM, with tumors and tumor areas differing in target expression. Intratumoral heterogeneity is described as the root cause of therapy resistance and might explain the failure of targeted therapies specifically targeting tumor biomarkers, including anti-EGFR (cetuximab, gefitinib, erlotinib …), anti-VEGF (bevacizumab) and anti-integrin (cilengitide) therapies. Below, we tried to explain the failure of the therapies targeting these three proteins. These data highlight the need to combine different targeted therapies.

### 4.1. The Failure of Anti-EGFR Therapies

Besides favourable pre-clinical studies, anti-EGFR therapies barely present any clinical benefit for patients with GBM. Several clinical studies are being carried out in newly diagnosed GBM and recurrent GBM with anti-EGFR therapies as monotherapy or in combination with radiochemotherapy or other targeted agents (Table 2).

Besides the tissue differences between colorectal, head and neck, lung cancers and GBM, EGFR is also molecularly heterogeneous among these cancers. First, EGFR mutations in GBMs (as EGFRvIII) occur within receptor extracellular domain while in lung cancers (as L858R) occur in the kinase domain. Interestingly, EGFRvIII mutation seems to appear at later stages of tumor development. This subclonal EGFR mutation is lost in certain recurrent tumors [369]. However, mutational switch can happen where the initial EGFR mutation is replaced by another in recurrent tumor [370]. EGFRvIII heterogeneity adds another layer of complexity by its location in extrachromosomal double minute structures. Extrachromosomal EGFRvIII loss upon treatment promotes therapy resistance. However, the mutant tends to reappear after TKI withdrawal and resensitizes the tumor [371]. The secondary mutation (T790M) upon TKI treatment provides tumor resistance to therapy, in lung cancer [372]. While, in GBM no EGFR secondary mutation is described as cause of therapy resistance [373].

Tumor heterogeneity can be a reasonable case for GBM resistance to EGFR-targeted therapies. Upregulation of redundant receptor tyrosine kinases and deregulation of EGFR downstream molecules can trigger EGFR therapy resistance.

In GBM, PDGFR and c-MET are also upregulated and contribute to tumor progression. In the same or in other subclones than EGFR, these receptors can mediate an EGFR-inhibition bypass. In vivo, inhibition of EGFR (erlotinib) and c-MET (crizotinib) resulted in decreased tumor growth [374]. Also, in a subcutaneous GBM xenografts, combined inhibition of EGFR and PDGFRβ signaling suppresses tumor growth [375]. Further clinical multi-targeting is needed to test this hypothesis and try to overcome EGFR-therapy resistance in GBM.

In GBM, an EGFR downstream molecule, PTEN, is often loss. PTEN is a suppressor of PI3K/AKT pathway. Simultaneous expression of EGFRvIII and PTEN was associated with patient response to TKI [376]. However, another study showed that even though PTEN is frequently deleted in GBM, it cannot predict therapeutic efficiency of TKI [140].

Moreover, EGFR therapeutic targeting promotes a switch to an angiogenic and mesenchymal tumor phenotype. Mesenchymal switch is associated with GBM therapy resistance [377,378]. GBM resistance to EGFR therapy is still unclear and further studies are needed to improve EGFR-targeting in clinical trials. Although multi-targeted RTK and combinatory therapies have been newly proposed (Table 2, Table 3 and Table 4) [379], there is an urgent need to develop genetic and cellular representative GBM models [380].

### 4.2. The Failure of Bevacizumab

The lack of efficacy of bevacizumab, a large-size molecule, can be explained by its intravenous route of administration and poor intracerebral bioavailability. Intra-arterial brain administration, after temporary destruction of the BBB by mannitol and followed by intravenous administration, has shown encouraging results in terms of PFS in patients with recurrent GBMs (PFS = 10 months) [381]. Indeed, this route of administration has the advantage of potentiating the cerebral delivery of chemotherapy (local concentration of more than 48.9-fold compared to intravenous administration) [382]. Recent results have confirmed the benefit of this delivery method and are being studied [383,384].

The standard dose of bevacizumab is 10 mg/kg IV, injected every two weeks. Although this dose is clinically well tolerated, it can have adverse biological effects, particularly via the formation of hypoxic areas [321]. The study by Heiland et al., 2016 [385] suggested that a low dose of bevacizumab may decrease the size of cerebral edema and may result in better vascular permeability. This study showed an improvement in PFS when bevacizumab is injected at 5 mg/kg every two weeks and is combined with lomustine, compared to bevacizumab alone at 10 mg/kg every two weeks (PFS = 5 months vs. 3.2 months). This therapeutic benefit was not observed in first-time recurrent patients. Finally, at a dose of 5 mg/kg/week, no gain in PFS or survival was observed [288].

### 4.3. The Failure of Cilengitide

Although preclinical studies nicely demonstrated that cilengitide may affect both tumoral cells and endothelial cells, failure to improve GBM patient survival of the first antagonist of integrins reaching the clinic was really disappointing. The reasons of this failure can only be guessed, but different factors may be included [386,387,388].

First, the short half-life (a few hours) and pharmacokinetics of cilengitide restricts its properties in patients. Second, the use of cilengitide at low dose has been shown to stimulate angiogenesis in preclinical models [389]. This point has been addressed in patients [390] where no cilengitide-specific pattern of progression has been detected. Third, no reliable biomarker of cilengitide activity has been identified for stratification of patients. For the CENTRIC assay (the phase III clinical trial), patients were stratified according to the MGMT promoter methylation status, i.e., inclusion concerned only patients with a methylated promoter [340]. A phase II clinical trial (CORE) was conducted concomitantly with patients exhibiting a non-methylated MGMT promoter. Interestingly, a retrospective analysis of both cohorts regarding the expression of the cilengitide targets (αvβ3/β5 integrins) expression, concluded that cilengitide was the most effective in the CORE patients with high level of αvβ3 expression in the tumoral cells and not in the endothelial cells [391]. These results highlight the need for stratification of patients at least based on the level of the predictive target. In line with this, it was recently shown in an elegant work from the Cheresh group, that GBM sensitivity to αvβ3 integrin blockade is not simply related to the overexpression of the integrin but rather to an addiction to glucose uptake by the glucose transporteur Glut3 [392,393]. A fourth point could be added concerning the redundancy of integrin targets; in fact, other integrins (such as α5β1 integrin) may remain active after cilengitide relaying pro-tumoral effects. The story of cilengitide highlights some pitfalls in the transfer of preclinical results towards the clinic but also the need to stratify patients according to pertinent biomarkers.

(iv) The plasticity of GBM cells complicates heterogeneity. It has been shown a bidirectional plasticity between glioma stem cell and their more differentiated counterparts either to form the tumor mass or in answer to therapies. These two types of cells will have different sensitivity to radio/chemotherapies but also to targeted therapies. Recent data emphasized that differentiated tumoral cells may contribute to GIC-dependent tumor progression [394,395]. These results indicate that targeting both cell populations will be needed to eradicate GBM. In a given tumor, glioma stem cells may vary from a proneuronal to a mesenchymal phenotype with intermediary states and thus acquiring new targets. Plasticity occurs also at the metabolic level when GBM cells adapt to the microenvironment to survive (for example from hypoxic to normoxic area) leading to new resistances. Treatments by themselves induce phenotypic and genomic modifications of tumor areas provoking secondary resistance. For example, bevacizumab has been shown to become ineffective due to the activation of secondary pathways involved in angiogenesis (c-MET, PIGF …).
(v) It is increasingly recognized that preclinical models have to be improved to reflect the clinical reality. In vitro, from 2D long term established cell lines grown on flat surface, 3D spheroids or cells embedded in several matrices, we now go through investigations on patient-derived primary cell lines either as glioma stem cell culture or as organoids. This last model certainly will recapitulate at best the tumoral and environmental heterogeneity of GBM. The deal for the following years will be to test therapies on such personalized models in a time framework which will allow to return towards the patient as rapidly as possible. Majority of in vivo models still are based on nude mice where immunological networks are absent. Even if syngeneic mice models of glioma can be useful, they lack the human specificities and complexities. Success of targeted therapies may be in part dependent on the development of reliable modeling of GBM.

Although targeting the immune system is not the subject of this review, this strategy is also part of many ongoing clinical investigations. Moreover, targeted therapy also mediates immunostimulatory and immunosuppressive effects [396]. While early results of checkpoint inhibitors or others immune-targeting drugs have been disappointing when used as monotherapy, likely because of the overwhelming immunosuppressive contribution of the immune tumor microenvironment (iTME), new combinatorial approach might overcome this issue. Interestingly, targeting microglia which is believed to be a major regulator of this iTME, has been suggested in combination with targeted or antiangiogenic therapies responsible of iTME modulation [397]. Indeed, VEGF and TGF-β signaling and abnormal vasculature, all belonging to the selected targets presented in this review has been implicated in fostering immunosuppression [398]. Their inhibition have been already shown to improved immunotherapies clinical outcomes in various cancer [399]. Although the impact of targeted therapies on iTME is still unclear, ongoing clinical trials combining bevacizumab or others targeted therapies to check-point inhibitors (for instance: NCT03743662, NCT03661723, NCT04704154) open new perspectives for GBM treatment.

## 5. Conclusions

Within molecular targeted therapies, the most frequently reported are those targeting (i) EGFR, which gene is amplified or over-expressed in more than 50% of GBMs (40 clinical trials), and more generally tyrosine kinase receptors (85 clinical trials) and (ii) VEGF/VEGFR (75 clinical trials of which 53 involving bevacizumab). Besides diagnostic and prognostic relevance, some markers can be of predictive interest (therapeutic decision making) or even constitute a molecular target that can be activated by a specific therapy (theranostic marker). It seems that new approaches aim to counter heterogeneity by targeting, not specifically certain tumor markers expressed irregularly, but the potential cause of the heterogeneity. New and combined approaches (targeted-, chemo-, immuno-, radiotherapies) may result in reduced secondary resistance because they target the whole tumor. Indeed, the discovery of GBM stem cells gave new hope for the treatment of GBM. Their likely significance in tumor initiation, and therefore in the heterogeneity of the GBM, makes them relevant targets but their differentiated counterparts need to be considered as well as their crosstalk only begin to be understood.

The 257 clinical trials described in tables of this manuscript reveal that many different options are explored and raised questions still unanswered about targeted therapies. However, they led to the accumulation of new fundamental knowledge, which will definitely help to understand the mechanisms of resistance and advance research. The results obtained in recent years highlight the need to better stratify patients, by providing more personalized treatment corresponding to the genetic composition and evolution of GBMs. In that way, initiatives such as N^2^M^2^ (NOA-20) phase I/II trial (NCT03158389) of molecularly matched targeted therapies plus radiotherapy in GBM patients, with an unmethylated MGMT promoter, appears of great interest [71]. In this trial, molecular profile characterization of tumors allows allocation of patients to first line targeted therapies according to their mode of action. Indeed, complex molecular diagnostics will translate in clinical decision and may be the future for GBM treatment.

## Figures and Tables

**Table 1:** Clinical studies analyzing therapies targeting the self-renewal of GICs.

Target	Molecule	Date	Protocol	Phase	Patients
Wnt pathway	**Celecoxib**				
	NCT00112502	06/2005–09/2014	Combined with TMZ	II	N
	Results (43 patients): PFS 10.5 months vs. 13.4 months; TMZ vs. TMZ + celecoxib (*p* = 0.97) [69]				
	NCT00047281	01/2003–07/2017	Combined with thalidomide, etoposide and Cyclophosphamide. Unpublished data	II	R
	NCT02770378	05/2016–10/2019	Combined with TMZ and eight repurposed drugs	I/II	R
	Results: ongoing studies (no recruitment)				
	NCT00068770	09/2003–03/2015	Combined with RT and anticonvulsant drugs (p450 inhibitor)	II	N undergoing RT and anticonvulsant treatment
	Results (35 patients): OS 11.5 months vs. 16 months (*p* = 0.11; HR = 2.7); p450 inhibitor vs. no p450 inhibitor [70]				
	*NCT00047294*	10/2002–06/2017	Thalidomide combined with the Stupp protocol and celecoxib	II	N
	*See Thalidomide*				
Notch pathway	**RO4929097**				
	NCT01122901	11/2010–03/2017	Monotherapy	II	R
	Results (47 patients): PFS 1.7 vs. 1.7 months; OS 6.6 months vs. 6.7 months; RO4929097 after vs. before resection (No statistical data)				
Hedgehog pathway	**Vismodegib GDC-0449**				
	NCT00980343	09/2009–08/2017	Monotherapy	II	R resectable
	Results (44 patients): PFS-6 0% vs. 0%; OS 7.8 vs. 7.6 months. Before surgical resection vs. without surgery (No statistical data)				
	NCT03158389	05/2017–02/2020	Molecularly Matched Targeted Therapies (APG101, alectinib, idasanutlin, atezolizumab, vismodegib, temsirolimus, palbociclib) combined with RT [71]	I/II	N without MGMT promoter methylation
	Results (350 patients): ongoing studies (recruitment)				
	**Glasdegib (PF-04449913)**				
	NCT03466450	03/2018–04/2020	Combined with TMZ	I/II	N
	Results: ongoing studies (recruitment)				
STAT3 pathway	**Napabucasin (BBI608)**				
	NCT02315534	12/2014–10/2019	Combined with TMZ	I/II	R
	Unpublished data				

R: recurrent GBM; N: newly diagnosed GBM; PFS: progression-free survival; PFS-6: 6-month survival; OS: overall survival. In red, not significant comparative tests. In italics, clinical trials listed in other tables (as mentioned). Results obtained from Clinicaltrials.com (accessed on 1 April 2020) and/or in cited references. Dates correspond to first posted and last update posted.

**Table 2:** Clinical studies analyzing therapies targeting EGFR and HER2.

Target	Molecule	Date	Protocol	Phase	Patients
EGFR	**Cetuximab**				
	NCT01044225	01/2010–03/2012	Combined with RT/TMZ and cilengitide (non-comparative)	II	N with MGMT-promoter unmethylated
	Unpublished data				
	NCT00311857	04/2006–09/2006	Combined with RT/TMZ	I/II	N
	Results (77 patients): PFS_6_ = 81%; PFS_12_ = 37%; OS_12_ = 87%; [109]				
	NCT00463073	04/2007–12/2008	Combined with bevacizumab and irinotecan	II	R
	Results (43 patients): PFS 16 weeks; OS 30 weeks [110]				
	NCT02800486	05/2016–01/2017	Intracranial monotherapy	II	N
	Results: ongoing studies (recruitment)				
	NCT01884740	06/2013–01/2017	Combined with bevacizumab and intracranial administration	I/II	N aged under 22
	Results: ongoing studies (recruitment)				
	NCT02861898	08/2016–05/2019	Intra-arterial combined with STUPP protocol	I/II	N
	Results: ongoing studies (recruitment)				
	**Panitumumab**				
	NCT01017653	11/2009–07/2016	Combined with irinotecan	II	R
	Results (16 patients): PFS-6 12.5%; OS 4.6 months				
	**Nimotuzumab**				
	NCT00753246	11/2007–11/2012	Combined with RT/TMZ vs. RT/TMZ	III	N
	Results (142 patients): PFS = 7.7 months vs. 5.8 months (*p* = 0.7989); OS = 22.3 months vs. 19.6 months (*p* = 0.485) Nimotuzumab + RT/TMZ vs. RT/TMZ [111]				
	NCT03388372	08/2010–01/2018	Combined with RT/TMZ	II	N
	Unpublished data				
	**Depatuxizumab-mafodotin**				
	NCT03419403	02/2018–04/2020	Combined with RT/TMZ and ophthalmologic prophylactic treatment	III	
	Unpublished data				
	NCT02573324	10/2015–04/2020	Combined with RT/TMZ	II/III	N with EGFR amplification
	Results: ongoing studies (no recruitment)				
	NCT02590263	10/2015–05/2019	Monotherapy or combined with RT/TMZ	I/II	N/R
	Results: ongoing studies (no recruitment)				
	NCT02343406	01/2015–05/2020	Monotherapy or combined with TMZ	II	R
	Results (260 patients): PFS = 2.7 vs. 1.9 vs. 1.9 months; OS = 9.6 vs. 7.9 vs. 8.2 months Depatux-M + TMZ vs. Depatux-M vs. Lomustine or TMZ				
	**GC1118**				
	NCT03618667	08/2018–08/2018	Monotherapy	II	R with high EGFR amplification
	Results: ongoing studies (recruitment)				
	**Sym004**				
	NCT02540161	09/2015–08/2019	Monotherapy	II	R
	Results: ongoing studies (no recruitment)				
	**Erlotinib**				
	NCT00337883	06/2006–03/2014	Monotherapy	II	R first
	Unpublished data				
	NCT00039494	01/2003–08/2013	Combined with TMZ/RT	I/II	N
	Results (100 patients): PFS 7.2 months; OS 15.3 months [112]				
	NCT00445588	03/2007–03/2016	Combined with sorafenib	II	R
	Results (56 patients): PFS 2.5 months; OS 5.7 months [113]				
	NCT00525525	09/2007–05/2014	Combined with bevacizumab. TMZ in adjuvant therapy	II	N
	Results (150 patients): PFS 9.2 months; OS 13.6 months [114]				
	NCT00187486	09/2005–08/2012	Combined with TMZ during the Stupp protocol	II	N
	Results (28 patients): PFS 2.8 months; OS 8.6 months [115]				
	NCT00720356	06/2008–10/2018	Combined with bevacizumab. in adjuvant therapy after RT/TMZ	II	N
	Results (48 patients): PFS-12 32%; OS 13.2 months				
	NCT00672243	01/2008–08/2013	Combined with sirolimus	II	R
	Results (32 patients): PFS 6.9 weeks; OS 33.8 weeks [116]				
	NCT00671970	01/2008–03/2013	Combined with bevacizumab	II	R
	Results (25 patients): PFS-6 28%; OS = 42 weeks [117]				
	NCT00086879	06/2004–09/2017	Monotherapy compared to TMZ or BCNU	II	R
	Results (110 patients): PFS 1.8 months vs. 2.4 months; OS 7.7 months vs. 7.3 months (No statistical data); Erlotinib vs. BCNU/TMZ [118]				
	NCT00301418	03/2006–02/2016	Monotherapy	I/II	R
	Results (11 patients): PFS 1.9 months; OS 6.9 months [119]				
	NCT00274833	01/2006–12/2012	Combined with TMZ/RT	II	N
	Unpublished data				
	NCT00387894	10/2006–06/2013	Monotherapy	II	R
	Results (6 patients): Terminated because ongoing literature at the time confirmed that the selection process was not likely to enrich for a patient population expected to benefit, and rapid disease progression in the first 6 patients.				
	NCT00054496	02/2003–01/2014	Monotherapy	II	R
	Results: ongoing studies (recruitment unknown)				
	NCT00112736	06/2005–06/2015	Combined with temsirolimus	I/II	R
	Results (47 patients): PFS-6 13% [120]				
	NCT01110876	04/2010–11/2014	Combined with vorinostat and TMZ	I/II	R
	Unpublished data				
	NCT00045110	01/2003–08/2017	Monotherapy	I/II	R/N
	Results (96 patients): PFS 2 months GBM R; OS 14 months GBM N Post RT [121]				
	*NCT00335764*	04/2006–07/2018	Sorafenib combined with erlotinib. tipifarnib or temsirolimus	I/II	R
	*See Sorafenib*				
	**Gefitinib**				
	NCT00238797	10/2005–01/2011	Combined with RT	II	-
	Unpublished data				
	NCT00250887	11/2005–10/2007	Pre- and post-surgery (second surgery)	II	R
	Results (22 patients): OS 8.8 months [122]				
	NCT00014170	04/2001–07/2013	Monotherapy	II	N
	Unpublished data				
	NCT00016991	06/2001–06/2013	Monotherapy	II	R first
	Results (53 patients): PFS 8.1 weeks; OS 39.4 weeks [123]				
HER2	NCT00052208	01/2003–06/2013	Combined with RT	I/II	N
	Results (147 patients): PFS 4.9 months; OS 11.0 months [124]				
	NCT00025675	01/2003–06/2018	Monotherapy	II	R
	No results posted				
	*NCT01310855*	03/2011–05/2017	Cediranib combined with gefitinib, compared to cediranib and placebo	II	R
	*See Cediranib*				
	**Afatinib**				
	NCT00727506	06/2008–06/2017	Monotherapy ± TMZ and compared with TMZ	II	R
	Results (119 patients): PFS 0.99 months vs. 1.53 months (*p* = 0.032) vs. 1.87 months (*p* = 0.204); 9.8 months vs. 8 months (*p* = 0.386) vs. 10.6 months (*p* = 0.119); Afatinib vs. Afatinib + TMZ vs. TMZ [125]				
	**Dacomitinib**				
	NCT01520870	01/2012–03/2018	Monotherapy	II	R with EGFR Amplification or EGFRvIII Mutation
	Results (49 patients): PFS-6 s 10.6%; PFS 2.7 months; OS 7.4 months [126]				
	NCT01112527	04/2010–08/2018	Monotherapy	II	R
	Unpublished data				
	**Lapatinib**				
	NCT01591577	05/2012–09/2016	Combined with or non- combined with RT/TMZ. Unpublished data	II	N
	NCT00099060	12/2004–01/2014	Monotherapy. Unpublished data	I/II	R
	NCT00107003	04/2005–07/22018	Pre-operatory monotherapy.	II	R
	Unpublished data				
	NCT00350727	07/2006–04/2013	Combined with pazopanib	II	R
	Results (41 patients): PFS 62 vs. 56 days; PFS-6 0 vs. 15%; Patients positive vs. negative for EGFRvIII and/or PTEN [127]				
	**Neratinib**				
	NCT02977780	11/2016–02/2020	Combined with TMZ vs. TMZ	II	N
	Results: ongoing studies (recruitment)				

R: recurrent GBM; N: newly diagnosed GBM; PFS: progression-free survival; PFS-6: 6-month survival; OS: overall sur-vival. In red, not significant comparative tests. In italics, clinical trials listed in other tables (as mentioned). Results obtained from Clinicaltrials.com (accessed on 1 April 2020) and/or in cited references. Dates correspond to first posted and last update posted.

**Table 3:** Clinical studies analyzing multi-kinase inhibitors.

Molecule	Date	Protocol	Phase	Patients
**Anlotinib**				
NCT04157478	11/2019–11/2019	Combined with Stupp protocol compared to Stupp protocol alone	II	N
Not yet recruiting				
NCT04004975	07/2019–07/2019	Monotherapy	I/II	R
Results: ongoing studies (recruitment)				
NCT04119674	10/2019–10/2019	Combined with Stupp protocol	I/II	N
Results: ongoing studies (recruitment)				
**Tesevatinib**				
NCT02844439	07/2016–02/2020	Monotherapy	II	R
Unpublished data				
**Dacomitinib/Afatinib** (see EGFR)				
**Cabozantinib**				
NCT01068782	02/2010–07/2014	Monotherapy	II	R first or second
Unpublished data				
**TG02**				
NCT02942264	10/2016–01/2020	Combined with TMZ and compared with TMW alone	I/II	R
Results: ongoing studies (recruitment)				
**Vandetamib**				
NCT00441142	02/2007–03/2019	Combined with TMZ during Stupp protocol compared to Stupp protocol (non- comparative)	I/II	N
Results (106 patients): OS 15.9 months vs. 16.6 months (*p* = 0.75); PFS 6.2 vs. 7.7 months; RT/TMZ vs. vandetanib + RT/TMZ (*p* = 0.61) [147]				
NCT00995007	10/2009–03/2016	Combined with carboplatin and then monotherapy compared to carboplatin alone	II	R
Results (64 patients): PFS-6 1.7% vs. 0.9%s; OS 5.6 months vs. 5.2 months carboplatin + vandetanib vs. carboplatin (No statistical data) [148]				
**Bosutinib**				
NCT01331291	04/2011–07/2016	Monotherapy	II	R
Results (9 patients): PFS 7.71 weeks; OS 50 weeks [149]				

R: recurrent GBM; N: newly diagnosed GBM; PFS: progression-free survival; PFS-6: 6-month survival; OS: overall sur-vival. Results obtained from Clinicaltrials.com (accessed on 1 April 2020) and/or in cited references. Dates correspond to first posted and last update posted.

**Table 4:** Clinical studies analyzing therapies targeting, PDGFR, IGFR, FGFR, ALK.

Target	Molecule	Date	Protocol	Phase	Patients
PDGFR	**Imatinib**				
	NCT00290771	04/2006–04/2011	Combined with hydroxyurea	II	R
	Results (231 patients): PFS 5.6 weeks; OS 26 weeks [151]				
	NCT00171938	09/2005–02/2017	Monotherapy in case of impossible re-operation	II	R
					Unresectable with PDGFR positive
	Unpublished data				
	NCT00154375	09/2005–04/2011	Combined with hydroxyurea compared with hydroxyurea alone	III	R
	Results (240 patients): PFS 6 weeks vs. 6 weeks (HR = 0.93); OS 21 weeks vs. 19 weeks (HR = 0.92); imatinib + hydroxyurea vs. hydroxyurea alone [155]				
	NCT00010049	01/2003–06/2018	Monotherapy	I/II	R
	Results (34 patients): PFS-6 3% [156]				
	NCT00039364	01/2003–07/2012	Monotherapy	II	R
	Results (51 patients): PFS-6 16% [157]				
	**Dasatinib**				
	NCT00892177	05/2009–10/2019	Combined with bevacizumab and compared with bevacizumab alone	II	R
	Results (121 patients): PFS 3.3 months vs. 3.5 months (*p* = 0.52; HR = 1.14); OS 7.3 months vs. 7.9 months (*p* = 0.7; HR = 0.92) bevacizumab + dasatinib vs. bevacizumab + placebo [158]				
	NCT00423735	01/2007–04/2017	Monotherapy	II	R
	Results (77 patients): PFS 1.7 vs. 1.8 months; OS = 6.5 vs. 8.9 months; 200 mg/j vs. 400 mg/j (No statistical data) [152]				
	NCT00948389	06/2008–08/2012	Combined with lomustine	I/II	R
	Results (28 patients): PFS 1.35 months; OS 6.4 months [159]				
	NCT00869401	03/2009–02/2020	Combined with RT/TMZ compared to placebo	I/II	N
	Results (196 patients): OS 15.6 vs. 19.3 months; PFS: 6.2 vs. 7.8 months; dasatinib vs. placebo				
	**Tandutinib**				
	NCT00379080	09/2006–04/2017	Monotherapy	I/II	R
	Results (31 patients): PFS-6 16%; OS 8.8 months [154]				
	NCT00667394	04/2008–10/2015	Combined with bevacizumab	II	R
	Results (41 patients): PFS 4.1 months; OS 11 months [153]				
	**Crenolanib**				
	NCT02626364	11/2015–06/2017	Monotherapy	II	R PDGFRA Gene Amplification
	Results: ongoing studies (recruitment)				
	**Sunitinib**				
	NCT01100177	04/2010–03/2013	Monotherapy before and during RT	II	N unresectable
	Results:(12 patients): PFS 7.7 weeks; OS 12.8 weeks [160]				
	NCT00923117	07/2009–09/2015	Monotherapy with or without bevacizumab	II	R
	Results (87 patients): PFS-6 0.92 vs. 1.08 months Bevacizumab resistant vs. naïve patients				
	NCT00535379	09/2007–08/2010	Monotherapy	II	R
	Results (40 patients): PFS 2.2 months; OS 9.2 months [161]				
	NCT02928575	01/2016–10/2016	Combined with TMZ/RT	II	N
	Results: ongoing studies (recruitment unknown)				
	NCT00606008	01/2008–11/2012	Monotherapy	II	R
	Results (16 patients): PFS 1.4 months; OS 12.6 months [162]				
	NCT03025893	01/2017–06/2017	Monotherapy (high dose)	II/III	R
	Results: ongoing studies (recruitment)				
	NCT00499473	07/2007–02/2016	Monotherapy	II	R
	Results (25 patients): OS 5.7 vs. 12.3 months; Patients non-EIAC (enzyme-inducing anticonvulsants) vs. EIAC				
	**Regorafenib**				
	NCT03970447	05/2019–03/2020	Combined with RT/TMZ	II/III	N/R
	Results: ongoing studies (recruitment)				
	NCT04051606	08/2019–02/2020	Monotherapy	II	R
	Results: ongoing studies (recruitment)				
	NCT02926222	10/2016–09/2018	Monotherapy	II	R
	Results: ongoing studies (recruitment)				
	**MEDI-575**				
	NCT01268566	12/2010–04/2017	Monotherapy	II	R
	Results (56 patients): PFS-6 15.4%; PFS 1.4 months; OS 9.7 months [163]				
	**Olaratumab (IMC-3G3)**				
	NCT00895180	05/2009–12/2017	Monotherapy compared to ramucirumab	II	R
	Results (80 patients): PFS-6 12.5% vs. 7.5%; OS 49.5 vs. 34.3 weeks; ramucirumab vs. olaratumab				
	**Ponatinib**				
	NCT02478164	06/2015–07/2018	Monotherapy	II	R Bevacizumab-Refractory
	Results (15 patients): PFS 28 days; OS 98 days [164]				
	**Leflunomide**				
	NCT00003293	06/2004–09/2012	Monotherapy compared to procarbazine	III	R
	Unpublished data				
IGFR	**Axl1717**				
	NCT01721577	11/2012–01/2015	Monotherapy	I/II	R
	Results (8 patients): PFS 8 weeks; OS 15 weeks [165]				
FGFR	**BGJ398**				
	NCT01975701	11/2013–12/2019	Monotherapy	II	R
	Results (26 patients): PFS 1.7 months; OS 6.74 months				
ALK	**Alectinib**				
	*NCT03158389*	05/2017–02/2020	Molecularly Matched Targeted Therapies (APG101, alectinib, idasanutlin, atezolizumab, vismodegib, temsirolimus, palbociclib) combined with RT [71]	I/II	N without MGMT promoter methylation
	*See Vismodegib*				

R: recurrent GBM; N: newly diagnosed GBM; PFS: progression-free survival; PFS-6: 6-month survival; OS: overall sur-vival. In italics, clinical trials listed in other tables (as mentioned). Results obtained from Clinicaltrials.com (accessed on 1 April 2020) and/or in cited references. Dates correspond to first posted and last update posted.

**Table 5:** Clinical studies analyzing therapies targeting mTOR, PI3K/mTOR, Akt & protein kinase c.

Target	Molecule	Date	Protocol	Phase	Patients
mTOR	**Temsirolimus**				
	NCT00800917	12/2008–01/2010	Combined with bevacizumab	II	R
	Results (13 patients): PFS 8 weeks; OS 15 weeks [175]				
	NCT00016328	05/2001–07/2013	Monotherapy	II	R
	Results (65 patients): PFS 2.3 weeks; OS 4.4 months [176]				
	NCT00329719	05/2006–10/2018	Combined with sorafenib ± surgery	I/II	R
	Results (102 patients): PFS 2.71 vs. 4.34 vs. 1.87 months; OS 6.55 vs. 6.74 vs. 3.93 months. Temsirolimus + sorafenib vs. temsirolimus + sorafenib + surgery vs. temsirolimus + sorafenib in patients treated with anti-VEGF (No statistical data) [177]				
	NCT01019434	11/2009–10/2016	Combined with RT, compared with RT/TMZ	II	N. unmethylated MGMT
	Results (111 patients): PFS 5.4 months vs. 6.0 months (*p* = 0.24; HR = 1.26); OS 14.8 months vs. 16.0 months (*p* = 0.47; HR = 1.2) temsirolimus/RT vs. TMZ/RT [178]				
	NCT00022724	01/2003–06/2018	Monotherapy	I/II	R
	Results (43 patients): 9 weeks [179]				
	*NCT00112736*	06/2005–06/2015	Combined with erlotinib	I/II	R
	*See Erlotinib*				
	*NCT00335764*	04/2006–07/2018	Sorafenib combined with erlotinib, tipifarnib or temsirolimus	I/II	R
	*See Sorafenib*				
	*NCT03158389*	05/2017–02/2020	Molecularly Matched Targeted Therapies (APG101, alectinib, idasanutlin, atezolizumab, vismodegib, temsirolimus, palbociclib) combined with RT [71]	I/II	N without MGMT promoter methylation
	*See Vismodegib*				
	**Sirolimus**				
	*NCT00672243*	01/2008–02/2013	Combined with erlotinib	II	R
	*See Erlotinib*				
	**Everolimus**				
	NCT00515086	08/2007–09/2011	Monotherapy	II	R
	Unpublished data				
	NCT00107237	04/2005–06/2013	Combined with AEE788 (inhibitor of the EGFR, HER-2, VEGFR family)	II	R
	Unpublished data				
	NCT01434602	09/2011–07/2017	Combined with sorafenib	II	R
	Results: ongoing studies				
	NCT00805961	12/2008–08/2013	Combined with Bevacizumab in adjuvant therapy after RT/TMZ	II	N
	Results (68 patients): PFS 11.3 months; OS 13.9 months [180]				
	NCT00553150	11/2007–02/2020	Combination of RT/TMZ then TMZ/everolimus	II	N
	Results (100 patients): PFS-12 6.4 months; OS-12 15.8 months [181]				
	NCT01062399	02/2010–05/2019	Combined with RT/TMZ	I/II	N
	Results (171 patients): PFS: 8.2 vs. 10.2 months (*p* = 0.79); OS: 16.5 vs. 21.2 months (*p* = 0.008); Patients with or without everolimus [182]				
	**ABI-009 (nab-Rapamycin)**				
	NCT03463265	08/2018–12/2020	Monotherapy or in combination with bevacuzimab or RT/TMZ or marizomib, or lomustine	II	R/N
	Results: ongoing studies (recruitment)				
PI3K	**Pictilisib**				
	NCT02430363	03/2013–01/2016	Monotherapy compared with pembrolizumab	I/II	R
	Unpublished data				
	**Buparlisib (BKM120)**				
	NCT01349660	04/2011–01/2017	Combined with bevacizumab	I/II	R
	Preliminary data (76 patients): PFS 2.8 vs. 5.3 months; OS 6.5 vs. 10.8 months; buparlisib + bevacizumab vs. bevacizumab alone (No statistical data)				
	NCT01339052	04/2011–03/2019	Monotherapy combined or not combined with surgery	II	R
	Results (65 patients): PFS 1.7 months; OS 9.8 months; Patients not submitted to surgery [165]				
	**Sonolisib (PX-866)**				
	NCT01259869	04/2015–02/2015	Monotherapy	II	R first
	Results (17 patients): PFS6 = 17% [183]				
	**Paxalisib (GDC-0084)**				
	NCT03522298	05/2018–03/2020	Monotherapy	II	N
	Results: ongoing studies (no recruitment)				
PI3K/mTOR	**Bimiralisib (PQR309)**				
	NCT02850744	08/2016–10/2018	Monotherapy	II	N
	Unpublished data				
Akt & protein kinase c	**Enzastaurin**				
	NCT00295815	02/2006–11/2016	Compared with lomustine	III	R
	Results (293 patients): PFS 1.51 months vs. 1.64 months (*p* = 0.08; HR = 1.28); OS 6.60 months vs. 7.13 months (*p* = 0.25; HR = 1.20) enzastaurin vs. lomustine [184]				
	NCT00509821	06/2007–04/2016	Combined with RT (before, during, after)	II	N
	Results (60 patients): PFS 6.6 months; OS 15.0 months [185]				
	NCT00402116	11/2006–10/2010	Combined with the Stupp protocol	I/II	N
	Unpublished Phase II results				
	NCT00586508	12/2007–10/2013	Combined with bevacizumab	II	N
	Results (40 patients): PFS 2.0 months; OS = 7.5 months [186]				
	NCT03776071	12/2018–05/2019	Combined with RT/TMZ	II	N
	Results: ongoing studies (recruitment)				

R: recurrent GBM; N: newly diagnosed GBM; PFS: progression-free survival; PFS-6: 6-month survival; OS: overall sur-vival. In red, not significant comparative tests. In italics, clinical trials listed in other tables (as mentioned). Results obtained from Clinicaltrials.com (accessed on 1 April 2020) and/or in cited references. Dates correspond to first posted and last update posted.

**Table 6:** Clinical studies analyzing therapies targeting Ras/MAPK/MEK.

Target	Molecule	Date	Protocol	Phase	Patients
Ras/MAPK	**TLN-4601**				
	NCT00730262	08/2008–12/2017	Monotherapy	II	R
	Results (20 patients): PFS-6 0%; OS 130 days [201]				
	**Sorafenib**				
	NCT00544817	10/2007–06/2016	Combined with the Stupp protocol in adjuvant therapy	II	N
	Results (47 patients): PFS 6 months; OS 12 months [202]				
	NCT00597493	01/2008–03/2013	Combined with TMZ	II	R
	Results (32 patients): PFS 6.4 weeks; OS 41.5 weeks [203]				
	*NCT00329719*	05/2006–11/2016	Combined with temsirolimus	II	R
	*See Temsirolimus*				
	NCT00335764	04/2006–07/2018	Combined with erlotinib. tipifarnib or temsirolimus	I/II	R
	Results not fully available				
	*NCT00445588*	03/2007–03/2016	Combined with erlotinib	II	R
	*See Erlotinib*				
	NCT00621686	02/2008–01/2017	Combined with bevacizumab	II	R
	Results (54 patients): PFS 2.9 months; OS 5.6 months [204]				
	*NCT01434602*	09/2011–06/2017	Combined with everolimus	II	R
	*See Everolimus*				
	NCT01817751	03/2013–05/2017	Combined with valproic acid and sildenafil	II	R
	Results: ongoing studies (recruitment)				
	**LY2228820**				
	NCT02364206	02/2015–08/2019	Combined with the Stupp protocol	II	N
	Unpublished data				
	**Atorvastatin**				
	NCT02029573	01/2014–08/2017	Combined with RT/TMZ	II	/
	Results (20 patients): PFS 9.1 months [205]				
	**Dabrafenib**				
	NCT03919071	04/2019–03/2020	Combined with trametinib (MEK inhibitor) post-RT	II	N
	Results: ongoing studies (recruitment)				
	**2-OHOA**				
	NCT04250922	01/2020–01/2020	Combined with RT/TMZ	II	R
	Results: ongoing studies (recruitment)				
MEK	**Binimetinib**				
	NCT03973918	06/2019–03/2020	Combined with encorafenib	II	R BRAF V600-Mutated HGG
	Results: ongoing studies (recruitment)				
	**Trametinib**				
	*NCT03919071*	04/2019–03/2020	Combined with dabrafenib post-RT	II	N
	*See Dabrafenib*				

R: recurrent GBM; N: newly diagnosed GBM; PFS: progression-free survival; PFS-6: 6-month survival; OS: overall sur-vival. In italics, clinical trials listed in other tables (as mentioned). Results obtained from Clinicaltrials.com (accessed on 1 April 2020) and/or in cited references. Dates correspond to first posted and last update posted.

**Table 7:** Clinical studies analyzing therapies targeting apoptosis.

Target	Molecule	Date	Protocol	Phase	Patients
CD95	**APG101**				
	NCT01071837	02/2010–06/2015	Combined with re-irradiation compared to re-irradiation alone	II	R
	Results (91 patients): PFS 2.5 months vs. 4.5 months (*p* = 0.0162; HR = 0.49); OS 11.5 months vs. 11.5 months; reirradation vs. reirradiation + APG101 [215]				
	*NCT03158389*	05/2017–02/2020	Molecularly Matched Targeted Therapies (APG101, alectinib, idasanutlin, atezolizumab, vismodegib, temsirolimus, palbociclib) combined with RT [71]	I/II	N without MGMT promoter methylation
	*See Vismodegib*				
DRD2/3	**ONC201**				
	NCT02525692	08/2015–01/2020	Monotherapy	II	R H3 K27M positive
	Results: (14 patients): OS 17 weeks; PFS 14 weeks [216]				
p53	**Gene therapy (SGT-53)**				
	NCT02340156	12/2014–03/2020	Combined with TMZ	II	R
	Unpublished data				
p53-MDM2	**Idasanutlin (RG7388)**				
	*NCT03158389*	05/2017–02/2020	Molecularly Matched Targeted Therapies (APG101, alectinib, idasanutlin, atezolizumab, vismodegib, temsirolimus, palbociclib) combined with RT [71]	I/II	N without MGMT promoter methylation
	*See Vismodegib*				
Bcl-2	**Gossypol**				
	NCT00540722	10/2007–03/2017	Monotherapy	II	R
	Results (56 patients): PFS 1.87 months; OS = 5.9 months				
Farnesyl transferase	**Tipifarnib**				
	NCT00050986	01/2003–08/2012	Combined with TMZ	I/II	R
	No published results				
	NCT00058097	04/2003–04/2013	Combined with RT	II	N
	Results (28 patients): PFS 42 days; OS 234.5 days [217]				
	NCT00005859	01/2003–06/2018	Monotherapy	I/II	R
	Results (67 patients): PFS 8 vs. 6 weeks (*p* = 0.01) patients non- EIAED vs. patients EIAED [218]				
	*NCT00335764*	04/2006–07/2018	Sorafenib combined with erlotinib. tipifarnib or temsirolimus	I/II	R
	*See Sorafenib*				
	**Lonafarnib**				
	NCT00038493	06/2002–10/2018	Combined with TMZ	II	R
	Unpublished data				

R: recurrent GBM; N: newly diagnosed GBM; PFS: progression-free survival; PFS-6: 6-month survival; OS: overall sur-vival. In red, not significant comparative tests. In green, significant comparative tests. In italics, clinical trials listed in other tables (as mentioned). Results obtained from Clinicaltrials.com (accessed on 1 April 2020) and/or in cited references. Dates correspond to first posted and last update posted.

**Table 8:** Clinical studies analyzing therapies targeting autophagy.

Target	Molecule	Date	Protocol	Phase	Patients
Autophagy	**Chloroquine**				
	NCT02432417	04/2015–06/2019	Combined with the Stupp protocol	II	N
	Results: ongoing studies				
	NCT00224978	09/2005–11/2009	Monotherapy	III	N
	Results (30 patients): OS 24 vs. 11 months; chloroquine-treated patients vs. controls [250]				
	NCT00486603	06/2007–07/2019	Combined with RT/TMZ	I/II	N
	Results (76 patients): OS 15.6 months [251]				

N: newly diagnosed GBM; OS: overall sur-vival. Results obtained from Clinicaltrials.com (accessed on 1 April 2020) and/or in cited references. Dates correspond to first posted and last update posted.

**Table 9:** Clinical studies analyzing therapies targeting the cell cycle (CDK4/6), multifaceted pathways (proteasome, histone deacetylase, TGFβ) and DNA repair (PARP).

Target	Molecule	Date	Protocol	Phase	Patients
CDK4/6	**Palbociclib (PD 0332991)**				
	NCT01227434	10/2010–07/2015	Monotherapy combined or not combined to surgery	II	R
					Rb positif
	Results (22 patients): PFS 5.14 weeks; OS 15.4 weeks [252]				
	*NCT03158389*	05/2017–02/2020	Molecularly Matched Targeted Therapies (APG101, alectinib, idasanutlin, atezolizumab, vismodegib, temsirolimus, palbociclib) combined with RT [71]	I/II	N without MGMT promoter methylation
	*See Vismodegib*				
	**Abemaciclib**				
	NCT02981940	12/2016–03/2020	Monotherapy combined or not combined to surgery	II	R
	Results: ongoing studies (no recruitment)				
Proteasome	**Bortezomib**				
	NCT03643549	08/2018–02/2020	Combined with TMZ	I/II	R
					MGMT unmethylated
	Results: ongoing studies (recruitment)				
	NCT00641706	03/2008–05/2014	Combined with vorinostat	II	R
	Results (37 patients): PFS 1.5 mois; OS 3.2 mois [253]				
	NCT00998010	10/2009–05/2019	Combined with TMZ/RT	II	N
	Unpublished data				
	*NCT00611325*	02/2008–03/2014	Combined with bevacizumab	II	R
	*See Bevacizumab*				
	**Marizomib**				
	NCT03345095	11/2017–06/2019	Combined with TMZ/RT	III	N
	Results (749 patients): ongoing studies (recruitment)				
	*NCT03463265*	08/2018–12/2020	Monotherapy (ABI-009) or in combination with bevacuzimab or RT/TMZ or ABI-009, or lomustine	II	R/N
	*See ABI-009*				
	*NCT02330562*	01/2015–03/2020	Combined with bevacuzimab	I/II	R
	*See Bevacizumab*				
Histone desacetylase	**Vorinostat**				
	NCT00555399	11/2007–12/2019	Combined with Isotretinoin and temozolomide	I/II	R
	Results: ongoing studies (no recruitment)				
	NCT00731731	08/2008–03/2020	Combined with TMZ/RT	II	N
	Preliminary results (107 patients): OS-15 months 54.6%; PFS 8.05 months				
	NCT00238303	10/2005–05/2014	Combined with surgery	II	R
	Results (68 patients): PFS 1.9 months; OS 5.7 months [254]				
	*NCT01110876*	04/2010–11/2014	Combined with erlotinib and TMZ	I/II	R
	*See Erlotinib*				
	*NCT00641706*	03/2008–05/2014	Combined with bortezomib	II	R
	*See Bortezomib*				
	*NCT01266031*	12/2010–07/2018	Bevacizumab in monotherapy vs. combined with vorinostat	I/II	R
	*See Bevacizumab*				
	*NCT01738646*	11/2012–02/2017	Combined with bevacizumab	II	R
	*See Bevacizumab*				
	*NCT00939991*	07/2009–06/2013	Combined with bevacizumab and TMZ	I/II	R
	*See Bevacizumab*				
	**Panobinostat (LBH589)**				
	NCT00848523	02/2009–07/2010	Monotherapy	II	R
	Unpublished data				
	**FR901228**				
	NCT00085540	06/2004–01/2017	Monotherapy	I/II	R
	Results (35 patients): PFS 8 weeks [255]				
TGFβ & TGFβR	**Trabedersen (AP12009)**				
	NCT00431561	02/2007–12/2013	Monotherapy vs. TMZ or PVC (procarbazine/lomustine/vincristine)	IIb	R
	Results (145 patients): In GBM patients, response and survival results were comparable among the 3 arms [256]				
	**Galunisertib (LY2157299)**				
	NCT01582269	04/2012–12/2019	Monotherapy or combined with lomustine	II	R
	Results: ongoing studies (no recruitment)				
	NCT01220271	10/2010–02/2017	Combined with TMZ/RT vs. TMZ/RT	I/II	N
	Results (56 patients): OS 18.2 vs. 17.9 months (HR = 1.2), PFS 7.6 vs. 11.5 months (HR = 1.8), patients treated with galunisertib combined with TMZ/RT vs. TMZ/RT [257]				
	**OKN-007**				
	NCT03649464	08/2018–03/2020	Monotherapy	I/II	R
	Not yet recruiting				
PARP	**Iniparib (BSI-201)**				
	NCT00687765	06/2008–07/2015	Combined with TMZ	I/II	N
	Results (81 patients): OS 22 months [258]				
	**Veliparib**				
	NCT02152982	06/2014–03/2020	Combined with TMZ	II/III	N
	Results: ongoing studies (no recruitment)				
	NCT03581292	07/2018–03/2020	Combined with RT/TMZ	II	N
					Negative H3 K27M or BRAFV600
	Results: ongoing studies (recruitment)				
	NCT01026493	12/2009-/07/2017	Combined with TMZ	I/II	R
	Results (215 patients): OS 10.3 vs. 10.7 months (*p* = 0.95; HR = 0.99) patients BEV-naïve low vs. high TMZ dose; OS 4.7 vs. 4.7 months (*p* = 0.93; HR = 0.93) patients BEV-failure low vs. high TMZ dose; PFS-6 17 vs. 4.4% patients BEV-naïve vs. BEV-failure [259]				
	**Olaparib**				
	NCT03212274	07/2017–03/2020	Monotherapy	II	IDH1/2 mutations
	Results: ongoing studies (recruitment)				
	*NCT02974621*	11/2016–03/2020	Cediranib combined with olaparib and compared to bevacizumab	II	R
	*See Cediranib*				
	**Pamiparib**				
	NCT03150862	05/2017–11/2019	Combined with RT/TMZ	I/II	R/N
	Results: ongoing studies (no recruitment)				
	NCT03914742	04/2019-/2020	Combined with TMZ	I/II	R IDH1/2 mutations
	Results: ongoing studies (recruitment)				

R: recurrent GBM; N: newly diagnosed GBM; PFS: progression-free survival; PFS-6: 6-month survival; OS: overall sur-vival. In red, not significant comparative tests. In italics, clinical trials listed in other tables (as mentioned). Results obtained from Clinicaltrials.com (accessed on 1 April 2020) and/or in cited references. Dates correspond to first posted and last update posted.

**Table 10:** Clinical studies analyzing therapies targeting VEGF and VEGFR.

Target	Molecule	Date	Protocol	Phase	Patients
VEGF	**Bevacizumab**				
	NCT01609790	06/2012–03/2020	Combined with trebananib	II	R
	Preliminary results (116 patients): OS 11.5 vs. 7.5 months (*p* = 0.09; HR = 1.46); PFS 4.8 vs. 4.2% (*p* = 0.04; HR = 1.51)				
	NCT00817284	01/2009–11/2011	Combined with RT/TMZ or RT/irinotecan	II	N
	Unpublished data				
	NCT01860638	05/2013–04/2018	Continuous treatment with Stupp, followed with Lomustine in first disease progression (PD1) and with chemotherapy in second progression (PD2)	II	R
	Results (296 patients): OS 6.4 vs. 5.5 months (HR = 1.04); PFS 2.3 vs. 1.8 months (HR = 0.70) PD1 lomustine bevacizumab vs. lomustine alone; PFS 2 vs. 2.2 months (HR = 0.70) PD2 bevacizumab chemotherapy vs. chemotherapy alone. No *p* values were reported [278]				
	NCT01115491	05/2010–12/2014	Combined with TMZ	II	R
	Results (32 patients): PFS 18.29 weeks; OS 31.43 weeks				
	NCT00590681	01/2008–09/2015	Combined with TMZ	II	N
	Unpublished data				
	NCT00979017	09/2009–03/2014	Combined with TMZ and irinotecan	II	N unresectable and multifocal
	Results (41 patients): OS 12 months; PFS 8.6 months [279]				
	NCT01186406	08/2010–02/2019	Combined with gliadel, TMZ and RT	II	N
	Results (41 patients): OS 19.4 months; PFS 11.3 months				
	NCT01903330	07/2013–11/2019	Combined with ERC1671 (vaccine) and granulocyte-macrophage colony-stimulating factor (GM-CSF) compared to combination with placebo	II	R
	Results: ongoing studies (recruitment)				
	NCT01443676	09/2011–11/2016	Combined with RT compared to RT alone	II	N in elderly
	Results (75 patients): PFS 7.6 vs. 4.8 months (*p* = 0.003); OS 12.1 vs. 12.2 months (*p* = 0.77); bevacizumab + RT vs. RT [280]				
	NCT02898012	09/2016–09/2016	Combined with TMZ	II	N age over 70
	Results (66 patients): OS 23.9 weeks; PFS 15.3 weeks [281]				
	NCT01149850	06/2010–02/2020	Combined with TMZ	II	N in elderly
	Results: ongoing studies (no recruitment)				
	NCT01004874	10/2009–02/2020	Combined with RT/TMZ followed by combination with TMZ/popotecan	II	/
	Preliminary results (80 patients): OS 17.2 months; PFS 11.1 months				
	NCT00735436	08/2008–02/2013	Combined with gliadel and irinotecan	II	N
	Results (18 patients): PFS 8 months; OS 13.5 months				
	NCT02698280	03/2016–07/2018	Combined with nimustine	II	R
	Unpublished data				
	NCT01266031	12/2010–07/2018	Monotherapy vs. combined with vorinostat	I/II	R
	Results (patients): OS 9.24 vs. 7.8 months; bevacizumab vs. bevacizumab + vorinostat				
	NCT01013285	11/2009–01/2016	Combined with TMZ and RT	II	N
	Results: ongoing studies (recruitment unknown)				
	NCT01738646	11/2012–02/2017	Combined with vorinostat	II	R
	Results (38 patients): PFS 3.7 months; OS 10.4 months; PFS-6 30% [282]				
	NCT00939991	07/2009–06/2013	Combined with vorinostat and TMZ	I/II	R
	Results (39 patients): PFS 6.7 months; OS 12.5 months; PFS-6 53.8%				
	NCT00337207	06/2006–02/2020	Monotherapy	II	R
	Results (54 patients): PFS-6 24%				
	NCT00268359	12/2005–07/2014	Combined with irinotecan	II	R
	Results (32 patients): PFS 23 weeks; PFS-6 38% OS-6 72% [283]				
	NCT00795665	11/2008–03/2020	Combined with carmustine	II	R
	Unpublished data				
	NCT02330562	01/2015–03/2020	Combined with marizomib	I/II	R
	Results: ongoing studies (no recruitment)				
	NCT00921167	06/2009–12/2013	Combined with irinotecan	II	R
	Results: completed, no results posted				
	NCT02157103	06/2014–05/2018	Subcutaneous monotherapy	II	R
	Results (3 patients): 66.7% decrease in radiation-related edema				
	NCT01209442	09/2010–04/2019	Combined with hypofractionated RT and TMZ	II	N
	Results (30 patients): PFS 14.3 months; OS 16.3 months [284]				
	NCT02120287	04/2014–05/2019	Combined with radiosurgery	II	R
	Results (16 patients): OS 11.73 months				
	NCT01102595	04/2010–08/2015	Combined with TMZ in neoadjuvant therapy of the Stupp protocol compared to the Stupp protocol	II	N, unresectable
	Results (102 patients): PFS 2.2 vs. 4.8 months (*p* = 0.10; HR = 0.70); OS 7.7 vs. 10.6 months (*p* = 0.07; HR = 0.68); TMZ vs. TMZ + bevacizumab [285]				
	NCT01022918	12/2009–09/2012	Combined with irinotecan in neoadjuvant and adjuvant therapy with TMZ, compared to neoadjuvant TMZ and Stupp	II	N, unresectable
	Results: (120 patients): PFS = 7.1 vs. 5.2 months (HR = 0.82); OS = 11.1 vs. 11.1 months; bevacizumab/Irinotecan vs. ctrl [286]				
	NCT00943826	07/2009–09/2017	Combined with TMZ during the Stupp protocol, compared to the Stupp protocol	III	N
	Results (921 patients): PFS 10.6 vs. 6.2 months (*p* < 0.001; HR = 0.64); OS 16.8 vs. 16.7 months (*p* = 0.1; HR = 0.88); bevacizumab + Stupp vs. Stupp [287]				
	NCT01067469	02/2010–03/2020	Low dose and combined with lomustine, compared to high dose bevacizumab alone	II	R
	Results (69 patients): PFS 4.34 vs. 4.11 months (*p* = 0.19); OS 9.6 vs. 8.3 months (*p* = 0.75); bevacizumab + lomustine vs. bevacizumab [288]				
	NCT00883298	04/2009–03/2017	Combined with TMZ twice a week	II	R
	Results (30 patients): PFS 5.5 months; OS 51 weeks [289]				
	NCT00345163	06/2006–05/2017	Combined with or not combined with irinotecan	II	R
	Results (167 patients): PFS-6 42.6% vs. 50.3% (*p* < 0.0001); PFS 4.2 vs. 5.6 months; OS 9.2 months vs. 8.7 months; bevacizumab alone vs. bevacizumab + irinotecan [276]				
	NCT01474239	11/2011–03/2016	Compared with fotemustine	II	R
	Results (91 patients): PFS 3.38 vs. 3.45 months; OS 7.3 vs. 8.7 months; bevacizumab vs. fotemustine (no statistical data) [290]				
	NCT02761070	05/2016–02/2019	Combined with high-dose TMZ compared to bevacizumab alone	III	R
	Results: ongoing studies (recruitment)				
	NCT02743078	04/2016–11/2019	Combined with Optune^®^	II	R Beva refractory or resistant to Beva
	Unpublished data				
	NCT01894061	07/2013–03/2020	Combined with NovoTTF	II	R
	Unpublished data				
	NCT01814813	03/2013–06/2019	Combined with vaccination (HSPPC-96) compared to bevacizumab alone	II	R
	Preliminary results (90 patients): PFS 3.7 vs. 2.5 vs. 5.3 months (*p* < 0.01); OS 6.6 vs. 9.2 vs. 10.7 months (*p* = 0.16); HSPPC-96 + Bevacizumab concomitant vs. HSPPC-96 + bevacizumab on progression vs. bevacizumab alone				
	NCT01730950	11/2012–03/2020	Combined with re-irradiation, compared to bevacizumab alone	II	R
	Preliminary results (170 patients): PFS 8.9 vs. 7.9% (*p* = 0.05; HR = 0.73); OS 25.1 vs. 21.6% (*p* = 0.46; HR = 0.98); bevacizumab alone vs. bevacizumab + RT				
	NCT00967330	08/2009–11/2015	Combined with RT, then in adjuvant therapy combined with Irinotecan compared to the Stupp protocol	II	N. MGMT non methylated
	Results (182 patients): PFS 5.99 vs. 9.7 months (HR = 0.57; *p* < 0.001); OS 16.6 vs. 17.5 months (HR = 1.02; *p* = 0.55); TMZ vs. bevacizumab + irinotecan [291]				
	NCT02343549	01/2015–07/2019	Combined with Optune^®^ and TMZ	II	N
	Results: ongoing studies (recruitment)				
	NCT01290939	02/2011–02/2018	Combined with lomustine	III	R
	Results (437 patients): PFS 4.2 vs. 1.5 months (HR = 0.49; *p* < 0.001); OS 9.1 vs. 8.6 months (HR = 0.95; *p* = 0.65); bevacizumab + lomustine vs. lomustine alone [292]				
	NCT00611325	02/2008–03/2014	Combined with bortezomib	II	R
	Results (56 patients): PFS 2 vs. 2.5 months; OS 8 vs. 6 moonths; PFS-6 25 vs. 28.6%; EIAED vs. non-EIAED				
	NCT01269853	01/2011–05/2019	Intracerebral administration	I/II	R
	Results: ongoing studies (recruitment)				
	NCT01811498	03/2013–05/2019	Intracerebral administration	I/II	N
	Results: ongoing studies (recruitment)				
	NCT02511405	07/2015–10/2018	Combined with VB-111 (antiangiogenic), compared to bevacizumab alone	III	R
	Results (256 patients): OS 6.8 vs. 7.9 months (*p* = 0.19; HR = 1.20) combined vs. bevacizumab alone [293]				
	NCT00612339	02/2008–05/2013	Combined with TMZ	II	Non resectable
	Results (41 patients): RR 24.4%				
	NCT03149003	05/2017–01/2020	Combined with DSP-7888 (peptide vaccine) compared to bevacizumab alone	II	R
	Results: ongoing studies (no recruitment)				
	NCT00501891	07/2007–05/2013	Combined with TMZ	II	R
	Results (32 patients): PFS 15.8 weeks; OS 37.1 weeks [294]				
	NCT00597402	01/2008–05/2014	Combined with RT/TMZ, then combined with irinotecan	II	N
	Results (75 patients): PFS 14.2 months; OS 21.2 months [295]				
	NCT00433381	02/2007–09/2018	Combined with irinotecan or combined with TMZ	II	R
	Unpublished data				
	NCT00613028	02/2008–06/2013	Combined with etoposide or TMZ	II	R Resistant to Beva/Irinotecan
	Results (22 patients): PFS 4.1 vs. 8.1 weeks; OS 12.6 vs. 19 weeks; PFS-6 0 vs. 7.7%; bevacizumab + TMZ vs. bevacizumab + etoposide				
	NCT00612430	02/2008–08/2013	Combined with etoposide	II	R
	Results (27 GBM et 32 grade III glioma patients): PFS6 40.6% & 44,4%; OS 63.1 & 44.4 weeks [296]				
	NCT00884741	04/2009–07/2019	Combined with adjuvant TMZ compared to the Stupp protocol	III	N
	Results (621 patients): PFS 10.7 months vs. 7.3 months (HR 0.79; *p* 0.007); OS 15.7 months vs. 16.1 months (HR 1.13; *p* 0.21) (bevacizumab + Stupp vs. Stupp + placebo) [297]				
	*NCT00463073*	04/2007–12/2008	Combined with cetuximab and irinotecan	II	R
	*See Cetuximab*				
	*NCT01884740*	06/2013–01/2017	Combined with cetuximab and intracranial administration	I/II	N aged under 22
	*See Cetuximab*				
	*NCT00525525*	09/2007–05/2014	Combined with erlotinib, TMZ in adjuvant therapy	II	N
	*See Erlotinib*				
	*NCT00720356*	06/2008–10/2018	Combined with erlotinib, in adjuvant therapy after RT/TMZ	II	N
	*See Erlotinib*				
	*NCT00671970*	01/2008–03/2013	Combined with erlotinib	II	R
	*See Erlotinib*				
	*NCT00892177*	05/2009–10/2019	Combined with dasatinib and compared with bevacizumab alone	II	R
	*See Dasatinib*				
	*NCT00667394*	04/2008–10/2015	Combined with tandutinib	II	R
	*See Tandutinib*				
	*NCT00923117*	07/2009–09/2015	Sunitinib in monotherapy with or without bevacizumab	II	R
	*See Sunitinib*				
	*NCT00800917*	12/2008–01/2010	Combined with temsirolimus	II	R
	*See Temsirolimus*				
	*NCT00805961*	12/2008–08/2013	Combined with everolimus in adjuvant therapy after RT/TMZ	II	N
	*See Everolimus*				
	*NCT03463265*	08/2018–12/2020	Monotherapy (ABI-009) or in combination with bevacuzimab or RT/TMZ or marizomib, or lomustine	II	R/N
	*See ABI-009*				
	*NCT01349660*	04/2011–01/2017	Combined with buparlisib	I/II	R
	*See Buparlisib*				
	*NCT00586508*	12/2007–10/2013	Combined with enzastaurin	II	N
	*See Enzastaurin*				
	*NCT00621686*	02/2008–01/2017	Combined with sorafenib	II	R
	*See Sorafenib*				
	*NCT01632228*	06/2012–02/2018	Onartuzumab combined or not with bevacizumab, compared to bevacizumab alone	II	R
	*See Onartuzumab*				
	*NCT01113398*	04/2010–12/2015	Rilotumumab combined with bevacizumab	II	R
	*See Rilotumumab*				
	*NCT01648348*	06/2012–05/2018	TRC105 combined with bevacizumab, compared to bevacizumab alone	II	R
	*See TRC105*				
	*NCT01564914*	03/2012–06/2019	TRC105 combined with bevacizumab	II	R treated with Bevacizumab
	*See TRC105*				
	*NCT01290263*	02/2011–07/2017	Trebananib combined or not with bevacizumab	I/II	R
	*See Trebananib*				
VEGFR	**Pazopanib**				
	NCT02331498	11/2014–07/2019	Combined with the Stupp protocol	I/II	N
	Results: ongoing studies (recruitment)				
	NCT00459381	04/2007–03/2017	Monotherapy	II	R
	Results (35 patients): PFS 12 weeks; OS 35 weeks; PFS-6 3% [298]				
	NCT01931098	08/2013–03/2020	Combined with topotecan	II	R
	Results (35 patients): OS 42 weeks; PFS 24 weeks; PFS-6 46%; OS-6 77% [277]				
	*NCT00350727*	07/2006–04/2013	Combined with lapatinib	II	R
	*See Lapatinib*				
	**Cediranib**				
	NCT01310855	03/2011–05/2017	Combined with Gefitinib, compared to cediranib and placebo	II	R
	Results (97 patients): PFS 3.6 vs. 2.8 months (*p* = 0.17; HR = 0.72); OS 7.2 months vs. 5.5 months (HR = 0.68); cediranib + gefetinib vs. cediranib + placebo [299]				
	NCT00777153	10/2008–12/2016	Monotherapy or combination with lomustine, compared with lomustine alone	III	R
	Results (325 patients): PFS 92 vs. 125 vs. 44 days (*p* = 0.90; 0.16; HR = 1.05; 0.76); OS 8 vs. 9.4 vs. 9.8 months (*p* = 0.10; 0.50; HR = 1.43; 1.15); cediranib vs. cediranib + lomustine vs. lomustine + placebo [300]				
	NCT02974621	11/2016–03/2020	Combined with olaparib and compared to bevacizumab	II	R
	Results: ongoing studies (no recruitment)				
	NCT01062425	02/2010–03/2020	Combined with TMZ in the Stupp protocol, compared to the Stupp protocol	II	N
	Preliminary data (149 patients): PFS 2.7 vs. 6.2 months (*p* = 0.03; HR = 0.67); OS 13.8 vs. 14.5 months (*p* = 0.44; HR = 0.87); Stupp vs. cediranib + Stupp				
	NCT00662506	04/2008–09/2017	Combined with TMZ/RT	II	N
	Unpublished data				
	NCT00305656	03/2006–08/2013	Monotherapy	II	R
	Results (31 patients): PFS 117 days; OS 227 days [301]				
	**Nintedanib**				
	NCT01251484	12/2010–10/2012	Monotherapy (after treatment with the Stupp protocol or with bevacizumab)	II	R
	Results (25 patients): PFS 1 vs. 1 month; OS 10 vs. 2 months (*p* < 0.02); previous treatment with Stupp vs. bevacizumab [302]				
	NCT01666600	06/2012–11/2017	Combined with RT, compared to RT alone	I/II	R
	Unpublished data				
	NCT01380782	06/2011–08/2014	Monotherapy	II	R whether or not treated with Bevacizumab
	Results (36 patients): PFS 28 vs. 28 days; OS 6.9 vs. 2.6 months; not treated with bevacizumab vs. 1st line with bevacizumab (No statistical data) [303]				
	Dovitinib				
	NCT01753713	12/2012–12/2017	Monotherapy	II	R whether or not treated with Bevacizumab
	Results (33 patients): PFS 2 vs. 1.8 months; OS 8 vs. 4.3 months; bevacizumab-naive vs. 1st line with bevacizumab				
	**Vandetanib (see Multikinase inhibitors)**				
	*NCT00441142*	02/2007–04/2017	Combined with the TMZ of the Stupp protocol	I/II	N
	*See Multikinase inhibitors*				
	*NCT00995007*	10/2009–02/2016	Combined with carpoblatin and compared to carboplatin alone	II	R
	*See Multikinase inhibitors*				
	**Vatalanib**				
	NCT00128700	08/2005–09/2012	Combined with TMZ/RT	I/II	N
	Results (20 patients): PFS 7.2 months; OS 16.2 months [304]				
	**Tivozanib**				
	NCT01846871	03/2013–01/2019	Monotherapy	II	R
	Results (10 patients): PFS-6 10%; PFS 2.3 months; OS 8.1 months [305]				
	**Axitinib**				
	NCT01562197	03/2012–01/2019	Monotherapy or combined with lomustine	II	R
	Unpublished data				
	NCT01508117	01/2012–09/2017	Combined with RT	II	N elderly
	Results (1 patient): OS 0.2 years				
	NCT03660761	09/2018–04/2019	Combined with TMZ	II	R
	Unpublished data				
	**CT-322**				
	NCT00562419	11/2007–10/2010	Combined with irinotecan	II	R
	Results: ongoing studies (recruitment unknown)				
	**Semaxanib (SU5416)**				
	NCT00004868	03/2003–06/2018	Monotherapy	I/II	R RT non-responder
	Unpublished data				
	**Tanibirumab**				
	NCT03856099	02/2019–03/2020	Monotherapy	II	R
	Results: ongoing studies (recruitment)				
	NCT03033524	01/2017–01/2017	Monotherapy	II	R
	Results: ongoing studies (recruitment unknown)				

R: recurrent GBM; N: newly diagnosed GBM; PFS: progression-free survival; PFS-6: 6-month survival; OS: overall sur-vival. In red, not significant comparative tests. In green, significant comparative tests. In italics, clinical trials listed in other tables (as mentioned). Results obtained from Clinicaltrials.com (accessed on 1 April 2020) and/or in cited references. Dates correspond to first posted and last update posted.

**Table 11:** Phase I/II clinical studies analyzing therapies targeting c-MET and its ligand HGF, PIGF and Endoglin (CD105).

Target	Molecule	Date	Protocol	Phase	Patients
c-MET	**Onartuzumab**				
	NCT01632228	06/2012–02/2018	Combined or not with bevacizumab, compared to bevacizumab alone	II	R
	Results (129 patients): PFS 3.9 months vs. 2.9 months (*p* = 0.7444; HR = 1.06); OS 8.8 months vs. 12.9 months (*p* = 0.1389; HR = 1.45); ornatuzumab + bevacizumab vs. placebo + bevacizumab [318]				
	**Cabozantinib**				
	NCT00704288	06/2008–06/2014	Monotherapy	II	R
	Results (152 patients): PFS 3.7 vs. 3.7 months; OS 7.7 months vs. 10.4 months; 140 mg/j vs. 100 mg/j (No statistical data) [319]				
HGF	**Rilotumumab**				
	NCT01113398	04/2010–12/2015	Combined with bevacizumab	II	R
	Results (60 patients):				
	PFS 4 weeks vs. 4.1 weeks (10 mg/kg vs. 20 mg/kg); OS = 3.6 months vs. 3.4 months in patients previously treated with bevacizumab				
	PFS 4.1 weeks vs. 4.7 weeks; OS 10.9 months vs. 11.4 months in patients previously untreated with bevacizumab [320]				
PIGF	**Aflibercept**				
	NCT00369590	08/2006–08/2015	Monotherapy	II	R
	Results (42 patients): PFS 12 weeks; OS 39 weeks [321]				
CD105	**TRC105**				
	NCT01648348	06/2012–05/2018	Combined with bevacizumab, compared to bevacizumab alone	II	R
	Results (101 patients): OS 9.7 vs. 7.4 months (HR = 1.06; *p* = 0.82); PFS-6 25 vs. 30.2%				
	NCT01564914	03/2012–06/2019	Combined with bevacizumab	II	R treated with Bevacizumab
	Results (22 patients): OS 5.75 months; PFS 1.81 vs. 1.30 patients receiving or not simultaneously bevacizumab				

R: recurrent GBM; N: newly diagnosed GBM; PFS: progression-free survival; PFS-6: 6-month survival; OS: overall sur-vival. In red, not significant comparative tests. In italics, clinical trials listed in other tables (as mentioned). Results obtained from Clinicaltrials.com (accessed on 1 April 2020) and/or in cited references. Dates correspond to first posted and last update posted.

**Table 12:** Clinical studies analyzing therapies targeting secondary pathways of angiogenesis.

Target	Molecule	Date	Protocol	Phase	Patients
β-FGF & TN	**Thalidomide**				
	NCT00412542	12/2006–02/2012	Combined with irinotecan	II	R
	Results (33 patients): PFS-6 25%; PFS 13 weeks; OS 36 weeks [336]				
	NCT00039468	06/2002–10/2011	Combined with irinotecan and RT	II	-
	Results (26 patients): PFS6 19% vs. 40%; recurrent vs. new (No statistical data) [337]				
	NCT00047294	10/2002–06/2017	Combined with the Stupp protocol and celecoxib	II	N
	Results (50 patients): PFS 5.9 months; OS 12.6 months [338]				
	NCT00521482	08/2007–08/2007	Combined with TMZ and compared TMZ alone	II	R
	Results: ongoing studies (recruitment unknown)				
	NCT00079092	03/2004–04/2017	Combined with procarbazine	II	R
	Unpublished data				
	NCT00006358	05/2004–06/2018	Combined with TMZ	II	R
	Results (44 patients): PFS 15 weeks [339]				
	*NCT00047281*	01/2003–07/2017	combined with celecoxib, etoposide and cyclophosphamide	II	R
	*See Celecoxib*				
Integrins	**Cilengitide**				
	NCT00689221	06/2008–11/2014	Combined with the Stupp protocol	III	N methylated MGMT status
	Results (926 patients): PFS 13.5 months vs. 10.7 months; Investigator (*p* = 0.46; HR = 0.93); PFS 10.6 months vs. 7.9 months (*p* = 0.41; HR = 0.92); Independent; OS 26.3 months vs. 26.3 months; cilengitide + Stupp vs. Stupp (*p* = 0.86; HR = 1.02) [340]				
	NCT00813943	12/2008–01/2017	Combined with the Stupp protocol	II	N non-methylated MGMT status
	Results (265 patients): PFS 5.6 vs. 5.9 (HR = 0.822) vs. 4.1 months (HR = 0.794); Independent PFS 6.4 vs. 7.5 (HR = 0.772) vs. 6.0 months (HR = 0.720) Investigator				
	OS 16.3 vs. 14.5 (*p* = 0.32; HR = 0.686) vs. 13.4 months (*p* = 0.3771; HR = 0.822);				
	cilengitide 2x/week vs. cilengitide 5x/week vs. Stupp [341]				
	*NCT01044225*	01/2010–03/2012	Combined with the Stupp protocol	II	N non-methylated MGMT status
	*See Cetuximab*				
	NCT00085254	06/2004–02/2016	Combined with RT/TMZ	II	N
	Results (112 patients): OS 19.7 months; OS 17.4 months (cilengitide 500 mg); OS 20.7 months (cilengitide 2000 mg);				
	OS 30 months (methylated MGMT); OS 17.4 months (non-methylated MGMT) [342]				
	NCT00112866	10/2004–10/2017	Monotherapy	II	R
	Results (26 patients): PFS-6 12%; PFS 8 weeks				
	NCT01124240	05/2010–07/2011	Combined with TMZ, RT and procarbazine	II	N Non Methylated
	Results: ongoing studies (recruitment unknown)				
	NCT00093964	10/2004–04/2019	Monotherapy	II	R
	Results (81 patients): PFS-6 7.5 vs. 15%; PFS 1.81 vs. 1.91 months; OS 6.54 vs. 9.91 months; Patients receiving 500 mg vs. 2000 mg [343]				
	NCT00006093	01/2003–06/2013	Monotherapy	I/II	R
	Unpublished data				
	**ATN-161**				
	NCT00352313	07/2006–05/2012	Combined with carboplatin	I/II	R
	Unpublished data				
Angiopoietin	**Trebananib (AMG-386)**				
	NCT01290263	02/2011–07/2017	Combined or not with bevacizumab	I/II	R
	Results (48 patients): OS 285 vs. 341 days; PFS 108 vs. 21 days; AMG-386 + bevacizumab vs. AMG-386 alone				
	*NCT01609790*	06/2012–03/2020	Combined with bevacizumab	II	R
	*See Bevacizumab*				
Target not clearly identified	**Recombinant Human Endostatin**				
	NCT04267978	02/2020–03/2020	Combined with TMZ and irinotecan	II	R
	Results: ongoing studies (recruitment)				
PSMA	**Prostate Specific Membrane Antigen (PSMA) ADC**				
	NCT01856933	05/2013–04/2019	Monotherapy	II	R
	Results (6 patients): No objective responses noted [344]				
MMP	**Prinomastat**				
	NCT00004200	05/2004–08/2012	Combined with TMZ/RT	II	N
	Unpublished data				

R: recurrent GBM; N: newly diagnosed GBM; PFS: progression-free survival; PFS-6: 6-month survival; OS: overall sur-vival. In red, not significant comparative tests. In italics, clinical trials listed in other tables (as mentioned). Results obtained from Clinicaltrials.com (accessed on 1 April 2020) and/or in cited references. Dates correspond to first posted and last update posted.

## Abbreviations

CNS	central nervous system
GBM	glioblastoma
HR	hazard ratio
OS	overall survival
PFS	progression-free survival

## References

[1] Stoyanov G.S., Dzhenkov D.L. (2018). On the Concepts and History of Glioblastoma Multiforme—Morphology, Genetics and Epigenetics. Folia Med..

[2] Lee S.C. (2018). Diffuse Gliomas for Nonneuropathologists: The New Integrated Molecular Diagnostics. Arch. Pathol. Lab. Med..

[3] Stupp R., Hegi M.E., Mason W.P., van den Bent M.J., Taphoorn M.J.B., Janzer R.C., Ludwin S.K., Allgeier A., Fisher B., Belanger K. (2009). Effects of Radiotherapy with Concomitant and Adjuvant Temozolomide versus Radiotherapy Alone on Survival in Glioblastoma in a Randomised Phase III Stud y: 5-Year Analysis of the EORTC-NCIC Trial. Lancet Oncol..

[4] Zhang J., Stevens M.F.G., Bradshaw T.D. (2012). Temozolomide: Mechanisms of Action, Repair and Resistance. Curr. Mol. Pharmacol..

[5] Prados M.D., Byron S.A., Tran N.L., Phillips J.J., Molinaro A.M., Ligon K.L., Wen P.Y., Kuhn J.G., Mellinghoff I.K., de Groot J.F. (2015). Toward Precision Medicine in Glioblastoma: The Promise and the Challenges. Neuro-Oncology.

[6] Franceschi E., Tosoni A., Bartolini S., Mazzocchi V., Fioravanti A., Brandes A.A. (2009). Treatment Options for Recurrent Glioblastoma: Pitfalls and Future Trends. Expert Rev. Anticancer Ther..

[7] Gallego O. (2015). Nonsurgical Treatment of Recurrent Glioblastoma. Curr. Oncol..

[8] Barani I.J., Larson D.A. (2015). Radiation Therapy of Glioblastoma. Cancer Treat. Res..

[9] Lawrie T.A., Evans J., Gillespie D., Erridge S., Vale L., Kernohan A., Grant R. (2019). Long-term Side Effects of Radiotherapy, with or without Chemotherapy, for Glioma. Cochrane Database Syst. Rev..

[10] Sun H., Du S., Liao G., Xie X., Ren C., Yuan Y.W. (2015). Do Glioma Patients Derive Any Therapeutic Benefit from Taking a Higher Cumulative Dose of Temozolomide Regimens?: A Meta-Analysis. Medicine.

[11] Stupp R., Brada M., van den Bent M.J., Tonn J.-C., Pentheroudakis G. (2014). High-Grade Glioma: ESMO Clinical Practice Guidelines for Diagnosis, Treatment and Follow-Up. Ann. Oncol. Off. J. Eur. Soc. Med. Oncol..

[12] Maschio M. (2012). Brain Tumor-Related Epilepsy. Curr. Neuropharmacol..

[13] Geraldo L.H.M., Garcia C., da Fonseca A.C.C., Dubois L.G.F., de Sampaio e Spohr T.C.L., Matias D., de Camargo Magalhães E.S., do Amaral R.F., da Rosa B.G., Grimaldi I. (2019). Glioblastoma Therapy in the Age of Molecular Medicine. Trends Cancer.

[14] Jain K.K. (2018). A Critical Overview of Targeted Therapies for Glioblastoma. Front. Oncol..

[15] Mooney J., Bernstock J.D., Ilyas A., Ibrahim A., Yamashita D., Markert J.M., Nakano I. (2019). Current Approaches and Challenges in the Molecular Therapeutic Targeting of Glioblastoma. World Neurosurg..

[16] Rajaratnam V., Islam M.M., Yang M., Slaby R., Ramirez H.M., Mirza S.P. (2020). Glioblastoma: Pathogenesis and Current Status of Chemotherapy and Other Novel Treatments. Cancers.

[17] Zanders E.D., Svensson F., Bailey D.S. (2019). Therapy for Glioblastoma: Is It Working?. Drug Discov. Today.

[18] Alemany M., Velasco R., Simó M., Bruna J. (2020). Late Effects of Cancer Treatment: Consequences for Long-Term Brain Cancer Survivors. Neuro-Oncol. Pract..

[19] Bobo R.H., Laske D.W., Akbasak A., Morrison P.F., Dedrick R.L., Oldfield E.H. (1994). Convection-Enhanced Delivery of Macromolecules in the Brain. Proc. Natl. Acad. Sci. USA.

[20] Akiyama Y., Kimura Y., Enatsu R., Mikami T., Wanibuchi M., Mikuni N. (2018). Advantages and Disadvantages of Combined Chemotherapy with Carmustine Wafer and Bevacizumab in Patients with Newly Diagnosed Glioblastoma: A Single-Institutional Experience. World Neurosurg..

[21] Vellimana A.K., Recinos V.R., Hwang L., Fowers K.D., Li K.W., Zhang Y., Okonma S., Eberhart C.G., Brem H., Tyler B.M. (2013). Combination of Paclitaxel Thermal Gel Depot with Temozolomide and Radiotherapy Significantly Prolongs Survival in an Experimental Rodent Glioma Model. J. Neurooncol..

[22] Westphal M., Hilt D.C., Bortey E., Delavault P., Olivares R., Warnke P.C., Whittle I.R., Jääskeläinen J., Ram Z. (2003). A Phase 3 Trial of Local Chemotherapy with Biodegradable Carmustine (BCNU) Wafers (Gliadel Wafers) in Patients with Primary Malignant Glioma. Neuro-Oncology.

[23] Wolinsky J.B., Colson Y.L., Grinstaff M.W. (2012). Local Drug Delivery Strategies for Cancer Treatment: Gels, Nanoparticles, Polymeric Films, Rods, and Wafers. J. Control. Release Off. J. Control. Release Soc..

[24] Xing W., Shao C., Qi Z., Yang C., Wang Z. (2015). The Role of Gliadel Wafers in the Treatment of Newly Diagnosed GBM: A Meta-Analysis. Drug Des. Devel. Ther..

[25] Zhang Y.-D., Dai R.-Y., Chen Z., Zhang Y.-H., He X.-Z., Zhou J. (2014). Efficacy and Safety of Carmustine Wafers in the Treatment of Glioblastoma Multiforme: A Systematic Review. Turk. Neurosurg..

[26] Karim R., Palazzo C., Evrard B., Piel G. (2016). Nanocarriers for the Treatment of Glioblastoma Multiforme: Current State-of-the-Art. J. Control. Release Off. J. Control. Release Soc..

[27] Gutkin A., Cohen Z.R., Peer D. (2016). Harnessing Nanomedicine for Therapeutic Intervention in Glioblastoma. Expert Opin. Drug Deliv..

[28] Kim S.-S., Harford J.B., Pirollo K.F., Chang E.H. (2015). Effective Treatment of Glioblastoma Requires Crossing the Blood-Brain Barrier and Targeting Tumors Including Cancer Stem Cells: The Promise of Nanomedicine. Biochem. Biophys. Res. Commun..

[29] Avril T., Vauleon E., Tanguy-Royer S., Mosser J., Quillien V. (2011). Mechanisms of Immunomodulation in Human Glioblastoma. Immunotherapy.

[30] Brown N.F., Carter T.J., Ottaviani D., Mulholland P. (2018). Harnessing the Immune System in Glioblastoma. Br. J. Cancer.

[31] McGranahan T., Therkelsen K.E., Ahmad S., Nagpal S. (2019). Current State of Immunotherapy for Treatment of Glioblastoma. Curr. Treat. Options Oncol..

[32] Miyazaki T., Ishikawa E., Sugii N., Matsuda M. (2020). Therapeutic Strategies for Overcoming Immunotherapy Resistance Mediated by Immunosuppressive Factors of the Glioblastoma Microenvironment. Cancers.

[33] Romani M., Pistillo M.P., Carosio R., Morabito A., Banelli B. (2018). Immune Checkpoints and Innovative Therapies in Glioblastoma. Front. Oncol..

[34] Fuchs Q., Pierrevelcin M., Messe M., Lhermitte B., Blandin A.-F., Papin C., Coca A., Dontenwill M., Entz-Werlé N. (2020). Hypoxia Inducible Factors’ Signaling in Pediatric High-Grade Gliomas: Role, Modelization and Innovative Targeted Approaches. Cancers.

[35] Fan Y., Potdar A.A., Gong Y., Eswarappa S.M., Donnola S., Lathia J.D., Hambardzumyan D., Rich J.N., Fox P.L. (2014). Profilin-1 Phosphorylation Directs Angiocrine Expression and Glioblastoma Progression through HIF-1α Accumulation. Nat. Cell Biol..

[36] Wang E., Zhang C., Polavaram N., Liu F., Wu G., Schroeder M.A., Lau J.S., Mukhopadhyay D., Jiang S.-W., O’Neill B.P. (2014). The Role of Factor Inhibiting HIF (FIH-1) in Inhibiting HIF-1 Transcriptional Activity in Glioblastoma Multiforme. PLoS ONE.

[37] Covello K.L., Kehler J., Yu H., Gordan J.D., Arsham A.M., Hu C.-J., Labosky P.A., Simon M.C., Keith B. (2006). HIF-2alpha Regulates Oct-4: Effects of Hypoxia on Stem Cell Function, Embryonic Development, and Tumor Growth. Genes Dev..

[38] Strowd R.E., Ellingson B.M., Wen P.Y., Ahluwalia M.S., Piotrowski A.F., Desai A.S., Clarke J.L., Lieberman F.S., Desideri S., Nabors L.B. (2019). Safety and Activity of a First-in-Class Oral HIF2-Alpha Inhibitor, PT2385, in Patients with First Recurrent Glioblastoma (GBM). J. Clin. Oncol..

[39] Clark P.M., Mai W.X., Cloughesy T.F., Nathanson D.A. (2016). Emerging Approaches for Targeting Metabolic Vulnerabilities in Malignant Glioma. Curr. Neurol. Neurosci. Rep..

[40] Woolf E.C., Scheck A.C. (2015). The Ketogenic Diet for the Treatment of Malignant Glioma. J. Lipid Res..

[41] Hay N. (2016). Reprogramming Glucose Metabolism in Cancer: Can It Be Exploited for Cancer Therapy?. Nat. Rev. Cancer.

[42] Ghiaseddin A.P., Shin D., Melnick K., Tran D.D. (2020). Tumor Treating Fields in the Management of Patients with Malignant Gliomas. Curr. Treat. Options Oncol..

[43] Ceccarelli M., Barthel F.P., Malta T.M., Sabedot T.S., Salama S.R., Murray B.A., Morozova O., Newton Y., Radenbaugh A., Pagnotta S.M. (2016). Molecular Profiling Reveals Biologically Discrete Subsets and Pathways of Progression in Diffuse Glioma. Cell.

[44] Perrin S.L., Samuel M.S., Koszyca B., Brown M.P., Ebert L.M., Oksdath M., Gomez G.A. (2019). Glioblastoma Heterogeneity and the Tumour Microenvironment: Implications for Preclinical Research and Development of New Treatments. Biochem. Soc. Trans..

[45] Verhaak R.G.W., Hoadley K.A., Purdom E., Wang V., Qi Y., Wilkerson M.D., Miller C.R., Ding L., Golub T., Mesirov J.P. (2010). Integrated Genomic Analysis Identifies Clinically Relevant Subtypes of Glioblastoma Characterized by Abnormalities in PDGFRA, IDH1, EGFR, and NF1. Cancer Cell.

[46] White K., Connor K., Clerkin J., Murphy B.M., Salvucci M., O’Farrell A.C., Rehm M., O’Brien D., Prehn J.H.M., Niclou S.P. (2020). New Hints towards a Precision Medicine Strategy for IDH Wild-Type Glioblastoma. Ann. Oncol..

[47] Aldape K., Zadeh G., Mansouri S., Reifenberger G., von Deimling A. (2015). Glioblastoma: Pathology, Molecular Mechanisms and Markers. Acta Neuropathol..

[48] Behnan J., Finocchiaro G., Hanna G. (2019). The Landscape of the Mesenchymal Signature in Brain Tumours. Brain J. Neurol..

[49] Tirosh I., Suvà M.L. (2020). Tackling the Many Facets of Glioblastoma Heterogeneity. Cell Stem Cell.

[50] Neftel C., Laffy J., Filbin M.G., Hara T., Shore M.E., Rahme G.J., Richman A.R., Silverbush D., Shaw M.L., Hebert C.M. (2019). An Integrative Model of Cellular States, Plasticity and Genetics for Glioblastoma. Cell.

[51] Bailey P., Cushing H. (1926). A Classification of Tumors of the Glioma Groupo on a Histogenetic Basis with a Correlation Study of Prognosis.

[52] Kernohan J.W., Mabon R.F. (1949). A Simplified Classification of the Gliomas. Proc. Staff Meet. Mayo Clin..

[53] Zülch K.J. (1979). Types Histologiques des Tumeurs du Système Nerveux Central.

[54] Kleihues P., Burger P.C., Scheithauer B.W. (1993). The New WHO Classification of Brain Tumours. Brain Pathol..

[55] Louis D.N., Perry A., Reifenberger G., von Deimling A., Figarella-Branger D., Cavenee W.K., Ohgaki H., Wiestler O.D., Kleihues P., Ellison D.W. (2016). The 2016 World Health Organization Classification of Tumors of the Central Nervous System: A Summary. Acta Neuropathol..

[56] Louis D.N., Ohgaki H., Wiestler O.D., Cavenee W.K., Burger P.C., Jouvet A., Scheithauer B.W., Kleihues P. (2007). The 2007 WHO Classification of Tumours of the Central Nervous System. Acta Neuropathol..

[57] Louis D.N., Holland E.C., Cairncross J.G. (2001). Glioma Classification: A Molecular Reappraisal. Am. J. Pathol..

[58] Louis D.N., Perry A., Burger P., Ellison D.W., Reifenberger G., von Deimling A., Aldape K., Brat D., Collins V.P., Eberhart C. (2014). International Society of Neuropathology—Haarlem Consensus Guidelines for Nervous System Tumor Classification and Grading. Brain Pathol..

[59] Singh S.K., Clarke I.D., Terasaki M., Bonn V.E., Hawkins C., Squire J., Dirks P.B. (2003). Identification of a Cancer Stem Cell in Human Brain Tumors. Cancer Res..

[60] Singh S.K., Clarke I.D., Hide T., Dirks P.B. (2004). Cancer Stem Cells in Nervous System Tumors. Oncogene.

[61] Cruceru M.L., Neagu M., Demoulin J.-B., Constantinescu S.N. (2013). Therapy Targets in Glioblastoma and Cancer Stem Cells: Lessons from Haematopoietic Neoplasms. J. Cell. Mol. Med..

[62] Ignatova T.N., Kukekov V.G., Laywell E.D., Suslov O.N., Vrionis F.D., Steindler D.A. (2002). Human Cortical Glial Tumors Contain Neural Stem-like Cells Expressing Astroglial and Neuronal Markers in Vitro. Glia.

[63] Yi Y., Hsieh I.-Y., Huang X., Li J., Zhao W. (2016). Glioblastoma Stem-Like Cells: Characteristics, Microenvironment, and Therapy. Front. Pharmacol..

[64] Koso H., Takeda H., Yew C.C.K., Ward J.M., Nariai N., Ueno K., Nagasaki M., Watanabe S., Rust A.G., Adams D.J. (2012). Transposon Mutagenesis Identifies Genes That Transform Neural Stem Cells into Glioma-Initiating Cells. Proc. Natl. Acad. Sci. USA.

[65] Iwadate Y. (2018). Plasticity in Glioma Stem Cell Phenotype and Its Therapeutic Implication. Neurol. Med. Chir..

[66] Chen W., Dong J., Haiech J., Kilhoffer M.-C., Zeniou M. (2016). Cancer Stem Cell Quiescence and Plasticity as Major Challenges in Cancer Therapy. Stem Cells Int..

[67] Jackson M., Hassiotou F., Nowak A. (2015). Glioblastoma Stem-like Cells: At the Root of Tumor Recurrence and a Therapeutic Target. Carcinogenesis.

[68] Sørensen M.D., Fosmark S., Hellwege S., Beier D., Kristensen B.W., Beier C.P. (2015). Chemoresistance and Chemotherapy Targeting Stem-like Cells in Malignant Glioma. Adv. Exp. Med. Biol..

[69] Penas-Prado M., Hess K.R., Fisch M.J., Lagrone L.W., Groves M.D., Levin V.A., De Groot J.F., Puduvalli V.K., Colman H., Volas-Redd G. (2015). Randomized Phase II Adjuvant Factorial Study of Dose-Dense Temozolomide Alone and in Combination with Isotretinoin, Celecoxib, and/or Thalidomide for Glioblastoma. Neuro-Oncology.

[70] Grossman S.A., Olson J., Batchelor T., Peereboom D., Lesser G., Desideri S., Ye X., Hammour T., Supko J.G. (2008). New Approaches to Brain Tumor Therapy CNS Consortium Effect of Phenytoin on Celecoxib Pharmacokinetics in Patients with Glioblastoma. Neuro-Oncology.

[71] Wick W., Dettmer S., Berberich A., Kessler T., Karapanagiotou-Schenkel I., Wick A., Winkler F., Pfaff E., Brors B., Debus J. (2019). N2M2 (NOA-20) Phase I/II Trial of Molecularly Matched Targeted Therapies plus Radiotherapy in Patients with Newly Diagnosed Non-MGMT Hypermethylated Glioblastoma. Neuro-Oncology.

[72] Nusse R. (2008). Wnt Signaling and Stem Cell Control. Cell Res..

[73] Yu C.-Y., Liang G.-B., Du P., Liu Y.-H. (2013). Lgr4 Promotes Glioma Cell Proliferation through Activation of Wnt Signaling. Asian Pac. J. Cancer Prev. APJCP.

[74] Bhuvanalakshmi G., Arfuso F., Millward M., Dharmarajan A., Warrier S. (2015). Secreted Frizzled-Related Protein 4 Inhibits Glioma Stem-like Cells by Reversing Epithelial to Mesenchymal Transition, Inducing Apoptosis and Decreasing Cancer Stem Cell Properties. PLoS ONE.

[75] Sareddy G.R., Kesanakurti D., Kirti P.B., Babu P.P. (2013). Nonsteroidal Anti-Inflammatory Drugs Diclofenac and Celecoxib Attenuates Wnt/β-Catenin/Tcf Signaling Pathway in Human Glioblastoma Cells. Neurochem. Res..

[76] Korur S., Huber R.M., Sivasankaran B., Petrich M., Morin P., Hemmings B.A., Merlo A., Lino M.M. (2009). GSK3beta Regulates Differentiation and Growth Arrest in Glioblastoma. PLoS ONE.

[77] Lee Y., Lee J.-K., Ahn S.H., Lee J., Nam D.-H. (2016). WNT Signaling in Glioblastoma and Therapeutic Opportunities. Lab. Investig. J. Tech. Methods Pathol..

[78] Ramachandran I., Ganapathy V., Gillies E., Fonseca I., Sureban S.M., Houchen C.W., Reis A., Queimado L. (2014). Wnt Inhibitory Factor 1 Suppresses Cancer Stemness and Induces Cellular Senescence. Cell Death Dis..

[79] Kanabur P., Guo S., Simonds G.R., Kelly D.F., Gourdie R.G., Verbridge S.S., Sheng Z. (2016). Patient-Derived Glioblastoma Stem Cells Respond Differentially to Targeted Therapies. Oncotarget.

[80] Li J.-L., Sainson R.C.A., Oon C.E., Turley H., Leek R., Sheldon H., Bridges E., Shi W., Snell C., Bowden E.T. (2011). DLL4-Notch Signaling Mediates Tumor Resistance to Anti-VEGF Therapy in Vivo. Cancer Res..

[81] Yu J.-B., Jiang H., Zhan R.-Y. (2016). Aberrant Notch Signaling in Glioblastoma Stem Cells Contributes to Tumor Recurrence and Invasion. Mol. Med. Rep..

[82] Shih I.-M., Wang T.-L. (2007). Notch Signaling, Gamma-Secretase Inhibitors, and Cancer Therapy. Cancer Res..

[83] Hovinga K.E., Shimizu F., Wang R., Panagiotakos G., Van Der Heijden M., Moayedpardazi H., Correia A.S., Soulet D., Major T., Menon J. (2010). Inhibition of Notch Signaling in Glioblastoma Targets Cancer Stem Cells via an Endothelial Cell Intermediate. Stem Cells Dayt. Ohio.

[84] Saito N., Fu J., Zheng S., Yao J., Wang S., Liu D.D., Yuan Y., Sulman E.P., Lang F.F., Colman H. (2014). A High Notch Pathway Activation Predicts Response to γ Secretase Inhibitors in Proneural Subtype of Glioma Tumor-Initiating Cells: Targeting Proneural GBM with Notch Inhibition. Stem Cells.

[85] Tanaka S., Nakada M., Yamada D., Nakano I., Todo T., Ino Y., Hoshii T., Tadokoro Y., Ohta K., Ali M.A.E. (2015). Strong Therapeutic Potential of γ-Secretase Inhibitor MRK003 for CD44-High and CD133-Low Glioblastoma Initiating Cells. J. Neurooncol..

[86] Yahyanejad S., King H., Iglesias V.S., Granton P.V., Barbeau L.M.O., van Hoof S.J., Groot A.J., Habets R., Prickaerts J., Chalmers A.J. (2016). NOTCH Blockade Combined with Radiation Therapy and Temozolomide Prolongs Survival of Orthotopic Glioblastoma. Oncotarget.

[87] Opačak-Bernardi T., Ryu J.S., Raucher D. (2017). Effects of Cell Penetrating Notch Inhibitory Peptide Conjugated to Elastin-like Polypeptide on Glioblastoma Cells. J. Drug Target..

[88] Pan E., Supko J.G., Kaley T.J., Butowski N.A., Cloughesy T., Jung J., Desideri S., Grossman S., Ye X., Park D.M. (2016). Phase I Study of RO4929097 with Bevacizumab in Patients with Recurrent Malignant Glioma. J. Neurooncol..

[89] Auffinger B., Spencer D., Pytel P., Ahmed A.U., Lesniak M.S. (2015). The Role of Glioma Stem Cells in Chemotherapy Resistance and Glioblastoma Multiforme Recurrence. Expert Rev. Neurother..

[90] Lee Y., Kim K.H., Kim D.G., Cho H.J., Kim Y., Rheey J., Shin K., Seo Y.J., Choi Y.-S., Lee J.-I. (2015). FoxM1 Promotes Stemness and Radio-Resistance of Glioblastoma by Regulating the Master Stem Cell Regulator Sox2. PLoS ONE.

[91] Tu Y., Niu M., Xie P., Yue C., Liu N., Qi Z., Gao S., Liu H., Shi Q., Yu R. (2017). Smoothened Is a Poor Prognosis Factor and a Potential Therapeutic Target in Glioma. Sci. Rep..

[92] Rimkus T.K., Carpenter R.L., Qasem S., Chan M., Lo H.-W. (2016). Targeting the Sonic Hedgehog Signaling Pathway: Review of Smoothened and GLI Inhibitors. Cancers.

[93] Lin T.L., Matsui W. (2012). Hedgehog Pathway as a Drug Target: Smoothened Inhibitors in Development. OncoTargets Ther..

[94] Bar E.E., Chaudhry A., Lin A., Fan X., Schreck K., Matsui W., Piccirillo S., Vescovi A.L., DiMeco F., Olivi A. (2007). Cyclopamine-Mediated Hedgehog Pathway Inhibition Depletes Stem-like Cancer Cells in Glioblastoma. Stem Cells Dayt. Ohio.

[95] Bensalma S., Chadeneau C., Legigan T., Renoux B., Gaillard A., de Boisvilliers M., Pinet-Charvet C., Papot S., Muller J.M. (2015). Evaluation of Cytotoxic Properties of a Cyclopamine Glucuronide Prodrug in Rat Glioblastoma Cells and Tumors. J. Mol. Neurosci..

[96] Liu Y.-J., Ma Y.-C., Zhang W.-J., Yang Z.-Z., Liang D.-S., Wu Z.-F., Qi X.-R. (2017). Combination Therapy with Micellarized Cyclopamine and Temozolomide Attenuate Glioblastoma Growth through Gli1 Down-Regulation. Oncotarget.

[97] de la Iglesia N., Puram S.V., Bonni A. (2009). STAT3 Regulation of Glioblastoma Pathogenesis. Curr. Mol. Med..

[98] Reguera-Nuñez E., Roca C., Hardy E., de la Fuente M., Csaba N., Garcia-Fuentes M. (2014). Implantable Controlled Release Devices for BMP-7 Delivery and Suppression of Glioblastoma Initiating Cells. Biomaterials.

[99] Rampazzo E., Dettin M., Maule F., Scabello A., Calvanese L., D’Auria G., Falcigno L., Porcù E., Zamuner A., Della Puppa A. (2017). A Synthetic BMP-2 Mimicking Peptide Induces Glioblastoma Stem Cell Differentiation. Biochim. Biophys. Acta.

[100] Garrido W., Rocha J.D., Jaramillo C., Fernandez K., Oyarzun C., San Martin R., Quezada C. (2014). Chemoresistance in High-Grade Gliomas: Relevance of Adenosine Signalling in Stem-like Cells of Glioblastoma Multiforme. Curr. Drug Targets.

[101] Uribe D., Torres Á., Rocha J.D., Niechi I., Oyarzún C., Sobrevia L., San Martín R., Quezada C. (2017). Multidrug Resistance in Glioblastoma Stem-like Cells: Role of the Hypoxic Microenvironment and Adenosine Signaling. Mol. Asp. Med..

[102] Quezada C., Garrido W., Oyarzún C., Fernández K., Segura R., Melo R., Casanello P., Sobrevia L., San Martín R. (2013). 5′-Ectonucleotidase Mediates Multiple-Drug Resistance in Glioblastoma Multiforme Cells. J. Cell. Physiol..

[103] Torres A., Vargas Y., Uribe D., Jaramillo C., Gleisner A., Salazar-Onfray F., López M.N., Melo R., Oyarzún C., San Martín R. (2016). Adenosine A3 Receptor Elicits Chemoresistance Mediated by Multiple Resistance-Associated Protein-1 in Human Glioblastoma Stem-like Cells. Oncotarget.

[104] Daniele S., Zappelli E., Natali L., Martini C., Trincavelli M.L. (2014). Modulation of A1 and A2B Adenosine Receptor Activity: A New Strategy to Sensitise Glioblastoma Stem Cells to Chemotherapy. Cell Death Dis..

[105] Nørøxe D.S., Poulsen H.S., Lassen U. (2016). Hallmarks of Glioblastoma: A Systematic Review. ESMO Open.

[106] Majewska E., Szeliga M. (2017). AKT/GSK3β Signaling in Glioblastoma. Neurochem. Res..

[107] Normanno N., De Luca A., Bianco C., Strizzi L., Mancino M., Maiello M.R., Carotenuto A., De Feo G., Caponigro F., Salomon D.S. (2006). Epidermal Growth Factor Receptor (EGFR) Signaling in Cancer. Gene.

[108] Frederick L., Wang X.Y., Eley G., James C.D. (2000). Diversity and Frequency of Epidermal Growth Factor Receptor Mutations in Human Glioblastomas. Cancer Res..

[109] Combs S.E., Schulz-Ertner D., Hartmann C., Welzel T., Timke C., Herfarth K., von Deimling A., Edler L., Platten M., Wick W. (2008). Phase I/II Study of Cetuximab plus Temozolomide as Radiochemotherapy for Primary Glioblastoma (GERT)—Eudract Number 2005–003911–63; NCT00311857. J. Clin. Oncol..

[110] Hasselbalch B., Lassen U., Hansen S., Holmberg M., Sørensen M., Kosteljanetz M., Broholm H., Stockhausen M.-T., Poulsen H.S. (2010). Cetuximab, Bevacizumab, and Irinotecan for Patients with Primary Glioblastoma and Progression after Radiation Therapy and Temozolomide: A Phase II Trial. Neuro-Oncology.

[111] Westphal M., Heese O., Steinbach J.P., Schnell O., Schackert G., Mehdorn M., Schulz D., Simon M., Schlegel U., Senft C. (2015). A Randomised, Open Label Phase III Trial with Nimotuzumab, an Anti-Epidermal Growth Factor Receptor Monoclonal Antibody in the Treatment of Newly Diagnosed Adult Glioblastoma. Eur. J. Cancer.

[112] Brown P.D., Krishnan S., Sarkaria J.N., Wu W., Jaeckle K.A., Uhm J.H., Geoffroy F.J., Arusell R., Kitange G., Jenkins R.B. (2008). Phase I/II Trial of Erlotinib and Temozolomide with Radiation Therapy in the Treatment of Newly Diagnosed Glioblastoma Multiforme: North Central Cancer Treatment Group Study N0177. J. Clin. Oncol..

[113] Peereboom D.M., Ahluwalia M.S., Ye X., Supko J.G., Hilderbrand S.L., Phuphanich S., Nabors L.B., Rosenfeld M.R., Mikkelsen T., Grossman S.A. (2013). NABTT 0502: A Phase II and Pharmacokinetic Study of Erlotinib and Sorafenib for Patients with Progressive or Recurrent Glioblastoma Multiforme. Neuro-Oncology.

[114] Raizer J.J., Giglio P., Hu J., Groves M., Merrell R., Conrad C., Phuphanich S., Puduvalli V.K., Loghin M., Paleologos N. (2016). A Phase II Study of Bevacizumab and Erlotinib after Radiation and Temozolomide in MGMT Unmethylated GBM Patients. J. Neurooncol..

[115] Peereboom D.M., Shepard D.R., Ahluwalia M.S., Brewer C.J., Agarwal N., Stevens G.H.J., Suh J.H., Toms S.A., Vogelbaum M.A., Weil R.J. (2010). Phase II Trial of Erlotinib with Temozolomide and Radiation in Patients with Newly Diagnosed Glioblastoma Multiforme. J. Neurooncol..

[116] Reardon D.A., Desjardins A., Vredenburgh J.J., Gururangan S., Friedman A.H., Herndon J.E., Marcello J., Norfleet J.A., McLendon R.E., Sampson J.H. (2010). Phase 2 Trial of Erlotinib plus Sirolimus in Adults with Recurrent Glioblastoma. J. Neurooncol..

[117] Sathornsumetee S., Desjardins A., Vredenburgh J.J., McLendon R.E., Marcello J., Herndon J.E., Mathe A., Hamilton M., Rich J.N., Norfleet J.A. (2010). Phase II Trial of Bevacizumab and Erlotinib in Patients with Recurrent Malignant Glioma. Neuro-Oncology.

[118] van den Bent M.J., Brandes A.A., Rampling R., Kouwenhoven M.C.M., Kros J.M., Carpentier A.F., Clement P.M., Frenay M., Campone M., Baurain J.-F. (2009). Randomized Phase II Trial of Erlotinib versus Temozolomide or Carmustine in Recurrent Glioblastoma: EORTC Brain Tumor Group Study 26034. J. Clin. Oncol..

[119] Kesavabhotla K., Schlaff C.D., Shin B., Mubita L., Kaplan R., Tsiouris A.J., Pannullo S.C., Christos P., Lavi E., Scheff R. (2012). Phase I/II Study of Oral Erlotinib for Treatment of Relapsed/Refractory Glioblastoma Multiforme and Anaplastic Astrocytoma. J. Exp. Ther. Oncol..

[120] Wen P.Y., Chang S.M., Lamborn K.R., Kuhn J.G., Norden A.D., Cloughesy T.F., Robins H.I., Lieberman F.S., Gilbert M.R., Mehta M.P. (2014). Phase I/II Study of Erlotinib and Temsirolimus for Patients with Recurrent Malignant Gliomas: North American Brain Tumor Consortium Trial 04-02. Neuro-Oncology.

[121] Raizer J.J., Abrey L.E., Lassman A.B., Chang S.M., Lamborn K.R., Kuhn J.G., Yung W.K.A., Gilbert M.R., Aldape K.A., Wen P.Y. (2010). A Phase II Trial of Erlotinib in Patients with Recurrent Malignant Gliomas and Nonprogressive Glioblastoma Multiforme Postradiation Therapy. Neuro-Oncology.

[122] Hegi M.E., Diserens A.-C., Bady P., Kamoshima Y., Kouwenhoven M.C.M., Delorenzi M., Lambiv W.L., Hamou M.-F., Matter M.S., Koch A. (2011). Pathway Analysis of Glioblastoma Tissue after Preoperative Treatment with the EGFR Tyrosine Kinase Inhibitor Gefitinib--a Phase II Trial. Mol. Cancer Ther..

[123] Rich J.N., Reardon D.A., Peery T., Dowell J.M., Quinn J.A., Penne K.L., Wikstrand C.J., Van Duyn L.B., Dancey J.E., McLendon R.E. (2004). Phase II Trial of Gefitinib in Recurrent Glioblastoma. J. Clin. Oncol..

[124] Chakravarti A., Wang M., Robins H.I., Lautenschlaeger T., Curran W.J., Brachman D.G., Schultz C.J., Choucair A., Dolled-Filhart M., Christiansen J. (2013). RTOG 0211: A Phase 1/2 Study of Radiation Therapy with Concurrent Gefitinib for Newly Diagnosed Glioblastoma Patients. Int. J. Radiat. Oncol. Biol. Phys..

[125] Reardon D.A., Nabors L.B., Mason W.P., Perry J.R., Shapiro W., Kavan P., Mathieu D., Phuphanich S., Cseh A., Fu Y. (2015). Phase I/Randomized Phase II Study of Afatinib, an Irreversible ErbB Family Blocker, with or without Protracted Temozolomide in Adults with Recurrent Glioblastoma. Neuro-Oncology.

[126] Sepúlveda-Sánchez J.M., Vaz M.Á., Balañá C., Gil-Gil M., Reynés G., Gallego Ó., Martínez-García M., Vicente E., Quindós M., Luque R. (2017). Phase II Trial of Dacomitinib, a Pan–Human EGFR Tyrosine Kinase Inhibitor, in Recurrent Glioblastoma Patients with EGFR Amplification. Neuro-Oncology.

[127] Reardon D.A., Groves M.D., Wen P.Y., Nabors L., Mikkelsen T., Rosenfeld S., Raizer J., Barriuso J., McLendon R.E., Suttle A.B. (2013). A Phase I/II Trial of Pazopanib in Combination with Lapatinib in Adult Patients with Relapsed Malignant Glioma. Clin. Cancer Res..

[128] Neyns B., Sadones J., Joosens E., Bouttens F., Verbeke L., Baurain J.-F., D’Hondt L., Strauven T., Chaskis C., In’t Veld P. (2009). Stratified Phase II Trial of Cetuximab in Patients with Recurrent High-Grade Glioma. Ann. Oncol..

[129] Solomon M.T., Miranda N., Jorrín E., Chon I., Marinello J.J., Alert J., Lorenzo-Luaces P., Crombet T. (2014). Nimotuzumab in Combination with Radiotherapy in High Grade Glioma Patients: A Single Institution Experience. Cancer Biol. Ther..

[130] Nitta Y., Shimizu S., Shishido-Hara Y., Suzuki K., Shiokawa Y., Nagane M. (2016). Nimotuzumab Enhances Temozolomide-Induced Growth Suppression of Glioma Cells Expressing Mutant EGFR in Vivo. Cancer Med..

[131] Lim Y., Yoo J., Kim M.-S., Hur M., Lee E.H., Hur H.-S., Lee J.-C., Lee S.-N., Park T.W., Lee K. (2016). GC1118, an Anti-EGFR Antibody with a Distinct Binding Epitope and Superior Inhibitory Activity against High-Affinity EGFR Ligands. Mol. Cancer Ther..

[132] Iida M., Brand T.M., Starr M.M., Li C., Huppert E.J., Luthar N., Pedersen M.W., Horak I.D., Kragh M., Wheeler D.L. (2013). Sym004, a Novel EGFR Antibody Mixture, Can Overcome Acquired Resistance to Cetuximab. Neoplasia.

[133] Montagut C., Argilés G., Ciardiello F., Poulsen T.T., Dienstmann R., Kragh M., Kopetz S., Lindsted T., Ding C., Vidal J. (2018). Efficacy of Sym004 in Patients with Metastatic Colorectal Cancer with Acquired Resistance to Anti-EGFR Therapy and Molecularly Selected by Circulating Tumor DNA Analyses: A Phase 2 Randomized Clinical Trial. JAMA Oncol..

[134] Phillips A.C., Boghaert E.R., Vaidya K.S., Mitten M.J., Norvell S., Falls H.D., DeVries P.J., Cheng D., Meulbroek J.A., Buchanan F.G. (2016). ABT-414, an Antibody-Drug Conjugate Targeting a Tumor-Selective EGFR Epitope. Mol. Cancer Ther..

[135] Van Den Bent M., Eoli M., Sepulveda J.M., Smits M., Walenkamp A., Frenel J.-S., Franceschi E., Clement P.M., Chinot O., De Vos F. (2020). INTELLANCE 2/EORTC 1410 Randomized Phase II Study of Depatux-M Alone and with Temozolomide vs Temozolomide or Lomustine in Recurrent EGFR Amplified Glioblastoma. Neuro-Oncology.

[136] Clarke J.L., Molinaro A.M., Phillips J.J., Butowski N.A., Chang S.M., Perry A., Costello J.F., DeSilva A.A., Rabbitt J.E., Prados M.D. (2014). A Single-Institution Phase II Trial of Radiation, Temozolomide, Erlotinib, and Bevacizumab for Initial Treatment of Glioblastoma. Neuro-Oncology.

[137] Uhm J.H., Ballman K.V., Wu W., Giannini C., Krauss J.C., Buckner J.C., James C.D., Scheithauer B.W., Behrens R.J., Flynn P.J. (2011). Phase II Evaluation of Gefitinib in Patients with Newly Diagnosed Grade 4 Astrocytoma: Mayo/North Central Cancer Treatment Group Study N0074. Int. J. Radiat. Oncol. Biol. Phys..

[138] Arkhipov A., Shan Y., Kim E.T., Dror R.O., Shaw D.E. (2013). Her2 Activation Mechanism Reflects Evolutionary Preservation of Asymmetric Ectodomain Dimers in the Human EGFR Family. eLife.

[139] Iqbal N., Iqbal N. (2014). Human Epidermal Growth Factor Receptor 2 (HER2) in Cancers: Overexpression and Therapeutic Implications. Mol. Biol. Int..

[140] Thiessen B., Stewart C., Tsao M., Kamel-Reid S., Schaiquevich P., Mason W., Easaw J., Belanger K., Forsyth P., McIntosh L. (2010). A Phase I/II Trial of GW572016 (Lapatinib) in Recurrent Glioblastoma Multiforme: Clinical Outcomes, Pharmacokinetics and Molecular Correlation. Cancer Chemother. Pharmacol..

[141] Yu A., Faiq N., Green S., Lai A., Green R., Hu J., Cloughesy T.F., Mellinghoff I., Nghiemphu P.L. (2017). Report of Safety of Pulse Dosing of Lapatinib with Temozolomide and Radiation Therapy for Newly-Diagnosed Glioblastoma in a Pilot Phase II Study. J. Neurooncol..

[142] Cicenas J., Cicenas E. (2016). Multi-Kinase Inhibitors, AURKs and Cancer. Med. Oncol..

[143] Shen G., Zheng F., Ren D., Du F., Dong Q., Wang Z., Zhao F., Ahmad R., Zhao J. (2018). Anlotinib: A Novel Multi-Targeting Tyrosine Kinase Inhibitor in Clinical Development. J. Hematol. Oncol..

[144] Goh K.C., Novotny-Diermayr V., Hart S., Ong L.C., Loh Y.K., Cheong A., Tan Y.C., Hu C., Jayaraman R., William A.D. (2012). TG02, a Novel Oral Multi-Kinase Inhibitor of CDKs, JAK2 and FLT3 with Potent Anti-Leukemic Properties. Leukemia.

[145] Du E., Li X., He S., Li X., He S. (2020). The Critical Role of the Interplays of EphrinB2/EphB4 and VEGF in the Induction of Angiogenesis. Mol. Biol. Rep..

[146] Jo M.-Y., Kim Y.G., Kim Y., Lee S.J., Kim M.H., Joo K.M., Kim H.H., Nam D.-H. (2012). Combined Therapy of Temozolomide and ZD6474 (Vandetanib) Effectively Reduces Glioblastoma Tumor Volume through Anti-Angiogenic and Anti-Proliferative Mechanisms. Mol. Med. Rep..

[147] Lee E.Q., Kaley T.J., Duda D.G., Schiff D., Lassman A.B., Wong E.T., Mikkelsen T., Purow B.W., Muzikansky A., Ancukiewicz M. (2015). A Multicenter, Phase II, Randomized, Noncomparative Clinical Trial of Radiation and Temozolomide with or without Vandetanib in Newly Diagnosed Glioblastoma Patients. Clin. Cancer Res..

[148] McNeill K., Iwamoto F., Kreisl T., Sul J., Shih J., Fine H. (2014). AT-39A randomized phase II trial of vandetanib (ZD6474) in combination with carboplatin versus carboplatin alone in adults with recurrent glioblastoma. Neuro-Oncology.

[149] Taylor J.W., Dietrich J., Gerstner E.R., Norden A.D., Rinne M.L., Cahill D.P., Stemmer-Rachamimov A., Wen P.Y., Betensky R.A., Giorgio D.H. (2015). Phase 2 Study of Bosutinib, a Src Inhibitor, in Adults with Recurrent Glioblastoma. J. Neurooncol..

[150] Joensuu H., Puputti M., Sihto H., Tynninen O., Nupponen N.N. (2005). Amplification of Genes Encoding KIT, PDGFRalpha and VEGFR2 Receptor Tyrosine Kinases Is Frequent in Glioblastoma Multiforme. J. Pathol..

[151] Reardon D.A., Dresemann G., Taillibert S., Campone M., van den Bent M., Clement P., Blomquist E., Gordower L., Schultz H., Raizer J. (2009). Multicentre Phase II Studies Evaluating Imatinib plus Hydroxyurea in Patients with Progressive Glioblastoma. Br. J. Cancer.

[152] Lassman A.B., Pugh S.L., Gilbert M.R., Aldape K.D., Geinoz S., Beumer J.H., Christner S.M., Komaki R., DeAngelis L.M., Gaur R. (2015). Phase 2 Trial of Dasatinib in Target-Selected Patients with Recurrent Glioblastoma (RTOG 0627). Neuro-Oncology.

[153] Odia Y., Sul J., Shih J.H., Kreisl T.N., Butman J.A., Iwamoto F.M., Fine H.A. (2016). A Phase II Trial of Tandutinib (MLN 518) in Combination with Bevacizumab for Patients with Recurrent Glioblastoma. CNS Oncol..

[154] Batchelor T.T., Gerstner E.R., Ye X., Desideri S., Duda D.G., Peereboom D., Lesser G.J., Chowdhary S., Wen P.Y., Grossman S. (2017). Feasibility, Phase I, and Phase II Studies of Tandutinib, an Oral Platelet-Derived Growth Factor Receptor-β Tyrosine Kinase Inhibitor, in Patients with Recurrent Glioblastoma. Neuro-Oncology.

[155] Dresemann G., Weller M., Rosenthal M.A., Wedding U., Wagner W., Engel E., Heinrich B., Mayer-Steinacker R., Karup-Hansen A., Fluge O. (2010). Imatinib in Combination with Hydroxyurea versus Hydroxyurea Alone as Oral Therapy in Patients with Progressive Pretreated Glioblastoma Resistant to Standard Dose Temozolomide. J. Neurooncol..

[156] Wen P.Y., Yung W.K.A., Lamborn K.R., Dahia P.L., Wang Y., Peng B., Abrey L.E., Raizer J., Cloughesy T.F., Fink K. (2006). Phase I/II Study of Imatinib Mesylate for Recurrent Malignant Gliomas: North American Brain Tumor Consortium Study 99-08. Clin. Cancer Res..

[157] Raymond E., Brandes A.A., Dittrich C., Fumoleau P., Coudert B., Clement P.M.J., Frenay M., Rampling R., Stupp R., Kros J.M. (2008). Phase II Study of Imatinib in Patients with Recurrent Gliomas of Various Histologies: A European Organisation for Research and Treatment of Cancer Brain Tumor Group Study. J. Clin. Oncol..

[158] Galanis E., Anderson S.K., Twohy E.L., Carrero X.W., Dixon J.G., Tran D.D., Jeyapalan S.A., Anderson D.M., Kaufmann T.J., Feathers R.W. (2019). A Phase 1 and Randomized, Placebo-Controlled Phase 2 Trial of Bevacizumab plus Dasatinib in Patients with Recurrent Glioblastoma: Alliance/North Central Cancer Treatment Group N0872. Cancer.

[159] Franceschi E., Stupp R., van den Bent M.J., van Herpen C., Laigle Donadey F., Gorlia T., Hegi M., Lhermitte B., Strauss L.C., Allgeier A. (2012). EORTC 26083 Phase I/II Trial of Dasatinib in Combination with CCNU in Patients with Recurrent Glioblastoma. Neuro-Oncology.

[160] Balaña C., Gil M.J., Perez P., Reynes G., Gallego O., Ribalta T., Capellades J., Gonzalez S., Verger E. (2014). Sunitinib Administered Prior to Radiotherapy in Patients with Non-Resectable Glioblastoma: Results of a Phase II Study. Target. Oncol..

[161] Hutterer M., Nowosielski M., Haybaeck J., Embacher S., Stockhammer F., Gotwald T., Holzner B., Capper D., Preusser M., Marosi C. (2014). A Single-Arm Phase II Austrian/German Multicenter Trial on Continuous Daily Sunitinib in Primary Glioblastoma at First Recurrence (SURGE 01-07). Neuro-Oncology.

[162] Pan E., Yu D., Yue B., Potthast L., Chowdhary S., Smith P., Chamberlain M. (2012). A Prospective Phase II Single-Institution Trial of Sunitinib for Recurrent Malignant Glioma. J. Neurooncol..

[163] Phuphanich S., Raizer J., Chamberlain M., Canelos P., Narwal R., Hong S., Miday R., Nade M., Laubscher K. (2017). Phase II Study of MEDI-575, an Anti-Platelet-Derived Growth Factor-α Antibody, in Patients with Recurrent Glioblastoma. J. Neurooncol..

[164] Lee E.Q., Muzikansky A., Reardon D.A., Dietrich J., Nayak L., Duda D.G., Chukwueke U.N., Beroukhim R., Doherty L.M., Kane C. (2018). Phase II Trial of Ponatinib in Patients with Bevacizumab-Refractory Glioblastoma. J. Clin. Oncol..

[165] Aiken R., Axelson M., Harmenberg J., Klockare M., Larsson O., Wassberg C. (2017). Phase I Clinical Trial of AXL1717 for Treatment of Relapsed Malignant Astrocytomas: Analysis of Dose and Response. Oncotarget.

[166] Grisanti S., Ferrari V.D., Buglione M., Agazzi G.M., Liserre R., Poliani L., Buttolo L., Gipponi S., Pedersini R., Consoli F. (2016). Second Line Treatment of Recurrent Glioblastoma with Sunitinib: Results of a Phase II Study and Systematic Review of Literature. J. Neurosurg. Sci..

[167] Fondevila F., Méndez-Blanco C., Fernández-Palanca P., González-Gallego J., Mauriz J.L. (2019). Anti-Tumoral Activity of Single and Combined Regorafenib Treatments in Preclinical Models of Liver and Gastrointestinal Cancers. Exp. Mol. Med..

[168] O’Hare T., Shakespeare W.C., Zhu X., Eide C.A., Rivera V.M., Wang F., Adrian L.T., Zhou T., Huang W.-S., Xu Q. (2009). AP24534, a Pan-BCR-ABL Inhibitor for Chronic Myeloid Leukemia, Potently Inhibits the T315I Mutant and Overcomes Mutation-Based Resistance. Cancer Cell.

[169] Shah G.D., Loizos N., Youssoufian H., Schwartz J.D., Rowinsky E.K. (2010). Rationale for the Development of IMC-3G3, a Fully Human Immunoglobulin G Subclass 1 Monoclonal Antibody Targeting the Platelet-Derived Growth Factor Receptor Alpha. Cancer.

[170] Zhang M., Liu J., Li M., Zhang S., Lu Y., Liang Y., Zhao K., Li Y. (2018). Insulin-like Growth Factor 1/Insulin-like Growth Factor 1 Receptor Signaling Protects against Cell Apoptosis through the PI3K/AKT Pathway in Glioblastoma Cells. Exp. Ther. Med..

[171] Maris C., D’Haene N., Trépant A.-L., Le Mercier M., Sauvage S., Allard J., Rorive S., Demetter P., Decaestecker C., Salmon I. (2015). IGF-IR: A New Prognostic Biomarker for Human Glioblastoma. Br. J. Cancer.

[172] Jimenez-Pascual A., Siebzehnrubl F.A. (2019). Fibroblast Growth Factor Receptor Functions in Glioblastoma. Cells.

[173] Lasorella A., Sanson M., Iavarone A. (2017). FGFR-TACC Gene Fusions in Human Glioma. Neuro-Oncology.

[174] Kawauchi D., Takahashi M., Yamamuro S., Kobayashi T., Uchida E., Iwadate Y., Ichimura K., Tomiyama A. (2020). ET-05 Alectinib and Ceritinib, the Second-Generation ALK Inhibitors, Effectively Induce Glioblastoma Cell Death. Neuro-Oncol. Adv..

[175] Lassen U., Sorensen M., Gaziel T.B., Hasselbalch B., Poulsen H.S. (2013). Phase II Study of Bevacizumab and Temsirolimus Combination Therapy for Recurrent Glioblastoma Multiforme. Anticancer Res..

[176] Galanis E., Buckner J.C., Maurer M.J., Kreisberg J.I., Ballman K., Boni J., Peralba J.M., Jenkins R.B., Dakhil S.R., Morton R.F. (2005). Phase II Trial of Temsirolimus (CCI-779) in Recurrent Glioblastoma Multiforme: A North Central Cancer Treatment Group Study. J. Clin. Oncol..

[177] Schiff D., Jaeckle K.A., Anderson S.K., Galanis E., Giannini C., Buckner J.C., Stella P., Flynn P.J., Erickson B.J., Schwerkoske J.F. (2018). Phase I/II Trial of Temsirolimus and Sorafenib in Treatment of Patients with Recurrent Glioblastoma: North Central Cancer Treatment Group Study/Alliance N0572. Cancer.

[178] Wick W., Gorlia T., Bady P., Platten M., van den Bent M.J., Taphoorn M.J.B., Steuve J., Brandes A.A., Hamou M.-F., Wick A. (2016). Phase II Study of Radiotherapy and Temsirolimus versus Radiochemotherapy with Temozolomide in Patients with Newly Diagnosed Glioblastoma without MGMT Promoter Hypermethylation (EORTC 26082). Clin. Cancer Res..

[179] Chang S.M., Wen P., Cloughesy T., Greenberg H., Schiff D., Conrad C., Fink K., Robins H.I., De Angelis L., Raizer J. (2005). Phase II Study of CCI-779 in Patients with Recurrent Glioblastoma Multiforme. Investig. New Drugs.

[180] Hainsworth J.D., Shih K.C., Shepard G.C., Tillinghast G.W., Brinker B.T., Spigel D.R. (2012). Phase II Study of Concurrent Radiation Therapy, Temozolomide, and Bevacizumab Followed by Bevacizumab/Everolimus as First-Line Treatment for Patients with Glioblastoma. Clin. Adv. Hematol. Oncol..

[181] Ma D.J., Galanis E., Anderson S.K., Schiff D., Kaufmann T.J., Peller P.J., Giannini C., Brown P.D., Uhm J.H., McGraw S. (2015). A Phase II Trial of Everolimus, Temozolomide, and Radiotherapy in Patients with Newly Diagnosed Glioblastoma: NCCTG N057K. Neuro-Oncology.

[182] Chinnaiyan P., Won M., Wen P.Y., Rojiani A.M., Werner-Wasik M., Shih H.A., Ashby L.S., Michael Yu H.-H., Stieber V.W., Malone S.C. (2018). A Randomized Phase II Study of Everolimus in Combination with Chemoradiation in Newly Diagnosed Glioblastoma: Results of NRG Oncology RTOG 0913. Neuro-Oncology.

[183] Pitz M.W., Eisenhauer E.A., MacNeil M.V., Thiessen B., Easaw J.C., Macdonald D.R., Eisenstat D.D., Kakumanu A.S., Salim M., Chalchal H. (2015). Phase II Study of PX-866 in Recurrent Glioblastoma. Neuro-Oncology.

[184] Wick W., Puduvalli V.K., Chamberlain M.C., van den Bent M.J., Carpentier A.F., Cher L.M., Mason W., Weller M., Hong S., Musib L. (2010). Phase III Study of Enzastaurin Compared with Lomustine in the Treatment of Recurrent Intracranial Glioblastoma. J. Clin. Oncol..

[185] Wick W., Steinbach J.P., Platten M., Hartmann C., Wenz F., von Deimling A., Shei P., Moreau-Donnet V., Stoffregen C., Combs S.E. (2013). Enzastaurin before and Concomitant with Radiation Therapy, Followed by Enzastaurin Maintenance Therapy, in Patients with Newly Diagnosed Glioblastoma without MGMT Promoter Hypermethylation. Neuro-Oncology.

[186] Odia Y., Iwamoto F.M., Moustakas A., Fraum T.J., Salgado C.A., Li A., Kreisl T.N., Sul J., Butman J.A., Fine H.A. (2016). A Phase II Trial of Enzastaurin (LY317615) in Combination with Bevacizumab in Adults with Recurrent Malignant Gliomas. J. Neurooncol..

[187] Chandrika G., Natesh K., Ranade D., Chugh A., Shastry P. (2017). Mammalian Target of Rapamycin Inhibitors, Temsirolimus and Torin 1, Attenuate Stemness-Associated Properties and Expression of Mesenchymal Markers Promoted by Phorbol-Myristate-Acetate and Oncostatin-M in Glioblastoma Cells. Tumour Biol. J. Int. Soc. Oncodev. Biol. Med..

[188] Lee E.Q., Kuhn J., Lamborn K.R., Abrey L., DeAngelis L.M., Lieberman F., Robins H.I., Chang S.M., Yung W.K.A., Drappatz J. (2012). Phase I/II Study of Sorafenib in Combination with Temsirolimus for Recurrent Glioblastoma or Gliosarcoma: North American Brain Tumor Consortium Study 05-02. Neuro-Oncology.

[189] Arcella A., Biagioni F., Antonietta Oliva M., Bucci D., Frati A., Esposito V., Cantore G., Giangaspero F., Fornai F. (2013). Rapamycin Inhibits the Growth of Glioblastoma. Brain Res..

[190] Mendiburu-Eliçabe M., Gil-Ranedo J., Izquierdo M. (2014). Efficacy of Rapamycin against Glioblastoma Cancer Stem Cells. Clin. Transl. Oncol..

[191] Ferrucci M., Biagioni F., Lenzi P., Gambardella S., Ferese R., Calierno M.T., Falleni A., Grimaldi A., Frati A., Esposito V. (2017). Rapamycin Promotes Differentiation Increasing βIII-Tubulin, NeuN, and NeuroD While Suppressing Nestin Expression in Glioblastoma Cells. Oncotarget.

[192] Kahn J., Hayman T.J., Jamal M., Rath B.H., Kramp T., Camphausen K., Tofilon P.J. (2014). The MTORC1/MTORC2 Inhibitor AZD2014 Enhances the Radiosensitivity of Glioblastoma Stem-like Cells. Neuro-Oncology.

[193] Shi F., Guo H., Zhang R., Liu H., Wu L., Wu Q., Liu J., Liu T., Zhang Q. (2017). The PI3K Inhibitor GDC-0941 Enhances Radiosensitization and Reduces Chemoresistance to Temozolomide in GBM Cell Lines. Neuroscience.

[194] Netland I.A., Førde H.E., Sleire L., Leiss L., Rahman M.A., Skeie B.S., Miletic H., Enger P.Ø., Goplen D. (2016). Treatment with the PI3K Inhibitor Buparlisib (NVP-BKM120) Suppresses the Growth of Established Patient-Derived GBM Xenografts and Prolongs Survival in Nude Rats. J. Neurooncol..

[195] Speranza M.-C., Nowicki M.O., Behera P., Cho C.-F., Chiocca E.A., Lawler S.E. (2016). BKM-120 (Buparlisib): A Phosphatidyl-Inositol-3 Kinase Inhibitor with Anti-Invasive Properties in Glioblastoma. Sci. Rep..

[196] Wen P.Y., Touat M., Alexander B.M., Mellinghoff I.K., Ramkissoon S., McCluskey C.S., Pelton K., Haidar S., Basu S.S., Gaffey S.C. (2019). Buparlisib in Patients with Recurrent Glioblastoma Harboring Phosphatidylinositol 3-Kinase Pathway Activation: An Open-Label, Multicenter, Multi-Arm, Phase II Trial. J. Clin. Oncol..

[197] Koul D., Shen R., Kim Y.-W., Kondo Y., Lu Y., Bankson J., Ronen S.M., Kirkpatrick D.L., Powis G., Yung W.K.A. (2010). Cellular and in Vivo Activity of a Novel PI3K Inhibitor, PX-866, against Human Glioblastoma. Neuro-Oncology.

[198] Ramezani S., Vousooghi N., Ramezani Kapourchali F., Joghataei M.T. (2017). Perifosine Enhances Bevacizumab-Induced Apoptosis and Therapeutic Efficacy by Targeting PI3K/AKT Pathway in a Glioblastoma Heterotopic Model. Apoptosis Int. J. Program. Cell Death.

[199] Alonso-Basanta M., Fang P., Maity A., Hahn S.M., Lustig R.A., Dorsey J.F. (2014). A Phase I Study of Nelfinavir Concurrent with Temozolomide and Radiotherapy in Patients with Glioblastoma Multiforme. J. Neurooncol..

[200] Narayan R.S., Fedrigo C.A., Brands E., Dik R., Stalpers L.J.A., Baumert B.G., Slotman B.J., Westerman B.A., Peters G.J., Sminia P. (2017). The Allosteric AKT Inhibitor MK2206 Shows a Synergistic Interaction with Chemotherapy and Radiotherapy in Glioblastoma Spheroid Cultures. BMC Cancer.

[201] Mason W.P., Belanger K., Nicholas G., Vallières I., Mathieu D., Kavan P., Desjardins A., Omuro A., Reymond D. (2012). A Phase II Study of the Ras-MAPK Signaling Pathway Inhibitor TLN-4601 in Patients with Glioblastoma at First Progression. J. Neurooncol..

[202] Hainsworth J.D., Ervin T., Friedman E., Priego V., Murphy P.B., Clark B.L., Lamar R.E. (2010). Concurrent Radiotherapy and Temozolomide Followed by Temozolomide and Sorafenib in the First-Line Treatment of Patients with Glioblastoma Multiforme. Cancer.

[203] Reardon D.A., Vredenburgh J.J., Desjardins A., Peters K., Gururangan S., Sampson J.H., Marcello J., Herndon J.E., McLendon R.E., Janney D. (2011). Effect of CYP3A-Inducing Anti-Epileptics on Sorafenib Exposure: Results of a Phase II Study of Sorafenib plus Daily Temozolomide in Adults with Recurrent Glioblastoma. J. Neurooncol..

[204] Galanis E., Anderson S.K., Lafky J.M., Uhm J.H., Giannini C., Kumar S.K., Kimlinger T.K., Northfelt D.W., Flynn P.J., Jaeckle K.A. (2013). Phase II Study of Bevacizumab in Combination with Sorafenib in Recurrent Glioblastoma (N0776): A North Central Cancer Treatment Group Trial. Clin. Cancer Res..

[205] Altwairgi A.K., Alghareeb W., Alnajjar F., Alsaeed E., Balbaid A., Alhussain H., Aldanan S., Orz Y., Lari A., Alsharm A. (2016). Phase II Study of Atorvastatin in Combination with Radiotherapy and Temozolomide In Patients with Glioblastoma (ART): Interim Analysis Report. Ann. Oncol..

[206] Wilhelm S., Carter C., Lynch M., Lowinger T., Dumas J., Smith R.A., Schwartz B., Simantov R., Kelley S. (2006). Discovery and Development of Sorafenib: A Multikinase Inhibitor for Treating Cancer. Nat. Rev. Drug Discov..

[207] Jakubowicz-Gil J., Bądziul D., Langner E., Wertel I., Zając A., Rzeski W. (2017). Temozolomide and Sorafenib as Programmed Cell Death Inducers of Human Glioma Cells. Pharmacol. Rep. PR.

[208] Riedel M., Struve N., Müller-Goebel J., Köcher S., Petersen C., Dikomey E., Rothkamm K., Kriegs M. (2016). Sorafenib Inhibits Cell Growth but Fails to Enhance Radio- and Chemosensitivity of Glioblastoma Cell Lines. Oncotarget.

[209] Zustovich F., Landi L., Lombardi G., Porta C., Galli L., Fontana A., Amoroso D., Galli C., Andreuccetti M., Falcone A. (2013). Sorafenib plus Daily Low-Dose Temozolomide for Relapsed Glioblastoma: A Phase II Study. Anticancer Res..

[210] Peng P., Wei W., Long C., Li J. (2017). Atorvastatin Augments Temozolomide’s Efficacy in Glioblastoma via Prenylation-Dependent Inhibition of Ras Signaling. Biochem. Biophys. Res. Commun..

[211] Azaro A., Plummer E.R., Urruticoechea A., Rodon J., Haris N.R.M., Veal G., Perier A., Tur V., Escriba P.V., Busquets X. (2017). Final Report of a Phase I Study of 2-Hydroxyoleic Acid (2OHOA) a Novel Sphingomyelin Synthase Activator in Patients (Pt) with Advanced Solid Tumors (AST) Including Recurrent High Grade Gliomas (RHGG). J. Clin. Oncol..

[212] Escamilla-Ramírez A., Castillo-Rodríguez R.A., Zavala-Vega S., Jimenez-Farfan D., Anaya-Rubio I., Briseño E., Palencia G., Guevara P., Cruz-Salgado A., Sotelo J. (2020). Autophagy as a Potential Therapy for Malignant Glioma. Pharmaceuticals.

[213] Kroemer G., Galluzzi L., Vandenabeele P., Abrams J., Alnemri E.S., Baehrecke E.H., Blagosklonny M.V., El-Deiry W.S., Golstein P., Green D.R. (2009). Classification of Cell Death: Recommendations of the Nomenclature Committee on Cell Death 2009. Cell Death Differ..

[214] Peter M.E., Krammer P.H. (2003). The CD95(APO-1/Fas) DISC and Beyond. Cell Death Differ..

[215] Wick W., Fricke H., Junge K., Kobyakov G., Martens T., Heese O., Wiestler B., Schliesser M.G., von Deimling A., Pichler J. (2014). A Phase II, Randomized, Study of Weekly APG101+reirradiation versus Reirradiation in Progressive Glioblastoma. Clin. Cancer Res..

[216] Chi A.S., Tarapore R.S., Hall M.D., Shonka N., Gardner S., Umemura Y., Sumrall A., Khatib Z., Mueller S., Kline C. (2019). Pediatric and Adult H3 K27M-Mutant Diffuse Midline Glioma Treated with the Selective DRD2 Antagonist ONC201. J. Neurooncol..

[217] Lustig R., Mikkelsen T., Lesser G., Grossman S., Ye X., Desideri S., Fisher J., Wright J. (2008). Phase II Preradiation R115777 (Tipifarnib) in Newly Diagnosed GBM with Residual Enhancing Disease. Neuro-Oncology.

[218] Cloughesy T.F., Wen P.Y., Robins H.I., Chang S.M., Groves M.D., Fink K.L., Junck L., Schiff D., Abrey L., Gilbert M.R. (2006). Phase II Trial of Tipifarnib in Patients with Recurrent Malignant Glioma Either Receiving or Not Receiving Enzyme-Inducing Antiepileptic Drugs: A North American Brain Tumor Consortium Study. J. Clin. Oncol..

[219] Drachsler M., Kleber S., Mateos A., Volk K., Mohr N., Chen S., Cirovic B., Tüttenberg J., Gieffers C., Sykora J. (2016). CD95 Maintains Stem Cell-like and Non-Classical EMT Programs in Primary Human Glioblastoma Cells. Cell Death Dis..

[220] Eisele G., Roth P., Hasenbach K., Aulwurm S., Wolpert F., Tabatabai G., Wick W., Weller M. (2011). APO010, a Synthetic Hexameric CD95 Ligand, Induces Human Glioma Cell Death in Vitro and in Vivo. Neuro-Oncology.

[221] Ralff M.D., Lulla A.R., Wagner J., El-Deiry W.S. (2017). ONC201: A New Treatment Option Being Tested Clinically for Recurrent Glioblastoma. Transl. Cancer Res..

[222] Kline C.L.B., Van den Heuvel A.P.J., Allen J.E., Prabhu V.V., Dicker D.T., El-Deiry W.S. (2016). ONC201 Kills Solid Tumor Cells by Triggering an Integrated Stress Response Dependent on ATF4 Activation by Specific EIF2α Kinases. Sci. Signal..

[223] Arrillaga-Romany I., Chi A.S., Allen J.E., Oster W., Wen P.Y., Batchelor T.T. (2017). A Phase 2 Study of the First Imipridone ONC201, a Selective DRD2 Antagonist for Oncology, Administered Every Three Weeks in Recurrent Glioblastoma. Oncotarget.

[224] Werry E.L., Barron M.L., Kassiou M. (2015). TSPO as a Target for Glioblastoma Therapeutics. Biochem. Soc. Trans..

[225] Werry E.L., King V.A., Barron M.L., Banister S.D., Sokias R., Kassiou M. (2017). Derivatives of the Pyrazolo[1,5-a]Pyrimidine Acetamide DPA-713 as Translocator Protein (TSPO) Ligands and pro-Apoptotic Agents in Human Glioblastoma. Eur. J. Pharm. Sci..

[226] England B., Huang T., Karsy M. (2013). Current Understanding of the Role and Targeting of Tumor Suppressor P53 in Glioblastoma Multiforme. Tumour Biol. J. Int. Soc. Oncodev. Biol. Med..

[227] Hong B., van den Heuvel A.P.J., Prabhu V.V., Zhang S., El-Deiry W.S. (2014). Targeting Tumor Suppressor P53 for Cancer Therapy: Strategies, Challenges and Opportunities. Curr. Drug Targets.

[228] Kim S.-S., Rait A., Kim E., Pirollo K.F., Chang E.H. (2015). A Tumor-Targeting P53 Nanodelivery System Limits Chemoresistance to Temozolomide Prolonging Survival in a Mouse Model of Glioblastoma Multiforme. Nanomed. Nanotechnol. Biol. Med..

[229] Costa B., Bendinelli S., Gabelloni P., Da Pozzo E., Daniele S., Scatena F., Vanacore R., Campiglia P., Bertamino A., Gomez-Monterrey I. (2013). Human Glioblastoma Multiforme: P53 Reactivation by a Novel MDM2 Inhibitor. PLoS ONE.

[230] Villalonga-Planells R., Coll-Mulet L., Martínez-Soler F., Castaño E., Acebes J.-J., Giménez-Bonafé P., Gil J., Tortosa A. (2011). Activation of P53 by Nutlin-3a Induces Apoptosis and Cellular Senescence in Human Glioblastoma Multiforme. PLoS ONE.

[231] Renner G., Janouskova H., Noulet F., Koenig V., Guerin E., Bär S., Nuesch J., Rechenmacher F., Neubauer S., Kessler H. (2016). Integrin A5β1 and P53 Convergent Pathways in the Control of Anti-Apoptotic Proteins PEA-15 and Survivin in High-Grade Glioma. Cell Death Differ..

[232] Canon J., Osgood T., Olson S.H., Saiki A.Y., Robertson R., Yu D., Eksterowicz J., Ye Q., Jin L., Chen A. (2015). The MDM2 Inhibitor AMG 232 Demonstrates Robust Antitumor Efficacy and Potentiates the Activity of P53-Inducing Cytotoxic Agents. Mol. Cancer Ther..

[233] Lebowitz P.F., Sakamuro D., Prendergast G.C. (1997). Farnesyl Transferase Inhibitors Induce Apoptosis of Ras-Transformed Cells Denied Substratum Attachment. Cancer Res..

[234] Basso A.D., Kirschmeier P., Bishop W.R. (2006). Lipid Posttranslational Modifications. Farnesyl Transferase Inhibitors. J. Lipid Res..

[235] Sebti S.M., Hamilton A.D. Farnesyltransferase Inhibitors in Cancer Therapy. https://www.buecher.de/shop/mund/farnesyltransferase-inhibitors-in-cancer-therapy/sebti-sad-m-hamilton-andrew-d-eds-/products_products/detail/prod_id/20963971/.

[236] Glass T.L., Liu T.J., Yung W.K. (2000). Inhibition of Cell Growth in Human Glioblastoma Cell Lines by Farnesyltransferase Inhibitor SCH66336. Neuro-Oncology.

[237] Chaponis D., Barnes J.W., Dellagatta J.L., Kesari S., Fast E., Sauvageot C., Panagrahy D., Greene E.R., Ramakrishna N., Wen P.Y. (2011). Lonafarnib (SCH66336) Improves the Activity of Temozolomide and Radiation for Orthotopic Malignant Gliomas. J. Neurooncol..

[238] Daniele S., Taliani S., Da Pozzo E., Giacomelli C., Costa B., Trincavelli M.L., Rossi L., La Pietra V., Barresi E., Carotenuto A. (2014). Apoptosis Therapy in Cancer: The First Single-Molecule Co-Activating P53 and the Translocator Protein in Glioblastoma. Sci. Rep..

[239] Daniele S., Barresi E., Zappelli E., Marinelli L., Novellino E., Da Settimo F., Taliani S., Trincavelli M.L., Martini C. (2016). Long Lasting MDM2/Translocator Protein Modulator: A New Strategy for Irreversible Apoptosis of Human Glioblastoma Cells. Oncotarget.

[240] Qiu J., Levin L.R., Buck J., Reidenberg M.M. (2002). Different Pathways of Cell Killing by Gossypol Enantiomers. Exp. Biol. Med..

[241] Jarzabek M.A., Amberger-Murphy V., Callanan J.J., Gao C., Zagozdzon A.M., Shiels L., Wang J., Ligon K.L., Rich B.E., Dicker P. (2014). Interrogation of Gossypol Therapy in Glioblastoma Implementing Cell Line and Patient-Derived Tumour Models. Br. J. Cancer.

[242] Jensen S.A., Day E.S., Ko C.H., Hurley L.A., Luciano J.P., Kouri F.M., Merkel T.J., Luthi A.J., Patel P.C., Cutler J.I. (2013). Spherical Nucleic Acid Nanoparticle Conjugates as an RNAi-Based Therapy for Glioblastoma. Sci. Transl. Med..

[243] Würstle S., Schneider F., Ringel F., Gempt J., Lämmer F., Delbridge C., Wu W., Schlegel J. (2017). Temozolomide Induces Autophagy in Primary and Established Glioblastoma Cells in an EGFR Independent Manner. Oncol. Lett..

[244] Yuan G., Yan S.-F., Xue H., Zhang P., Sun J.-T., Li G. (2014). Cucurbitacin I Induces Protective Autophagy in Glioblastoma in Vitro and in Vivo. J. Biol. Chem..

[245] Liu R., Li J., Zhang T., Zou L., Chen Y., Wang K., Lei Y., Yuan K., Li Y., Lan J. (2014). Itraconazole Suppresses the Growth of Glioblastoma through Induction of Autophagy: Involvement of Abnormal Cholesterol Trafficking. Autophagy.

[246] Zhang P., Sun S., Li N., Ho A.S.W., Kiang K.M.Y., Zhang X., Cheng Y.S., Poon M.W., Lee D., Pu J.K.S. (2017). Rutin Increases the Cytotoxicity of Temozolomide in Glioblastoma via Autophagy Inhibition. J. Neurooncol..

[247] Angeletti F., Fossati G., Pattarozzi A., Würth R., Solari A., Daga A., Masiello I., Barbieri F., Florio T., Comincini S. (2016). Inhibition of the Autophagy Pathway Synergistically Potentiates the Cytotoxic Activity of Givinostat (ITF2357) on Human Glioblastoma Cancer Stem Cells. Front. Mol. Neurosci..

[248] Maycotte P., Aryal S., Cummings C.T., Thorburn J., Morgan M.J., Thorburn A. (2012). Chloroquine Sensitizes Breast Cancer Cells to Chemotherapy Independent of Autophagy. Autophagy.

[249] Liu X., Sun K., Wang H., Dai Y. (2016). Inhibition of Autophagy by Chloroquine Enhances the Antitumor Efficacy of Sorafenib in Glioblastoma. Cell. Mol. Neurobiol..

[250] Sotelo J., Briceño E., López-González M.A. (2006). Adding Chloroquine to Conventional Treatment for Glioblastoma Multiforme: A Randomized, Double-Blind, Placebo-Controlled Trial. Ann. Intern. Med..

[251] Rosenfeld M.R., Ye X., Supko J.G., Desideri S., Grossman S.A., Brem S., Mikkelson T., Wang D., Chang Y.C., Hu J. (2014). A Phase I/II Trial of Hydroxychloroquine in Conjunction with Radiation Therapy and Concurrent and Adjuvant Temozolomide in Patients with Newly Diagnosed Glioblastoma Multiforme. Autophagy.

[252] Taylor J.W., Parikh M., Phillips J.J., James C.D., Molinaro A.M., Butowski N.A., Clarke J.L., Oberheim-Bush N.A., Chang S.M., Berger M.S. (2018). Phase-2 Trial of Palbociclib in Adult Patients with Recurrent RB1-Positive Glioblastoma. J. Neurooncol..

[253] Friday B.B., Anderson S.K., Buckner J., Yu C., Giannini C., Geoffroy F., Schwerkoske J., Mazurczak M., Gross H., Pajon E. (2012). Phase II Trial of Vorinostat in Combination with Bortezomib in Recurrent Glioblastoma: A North Central Cancer Treatment Group Study. Neuro-Oncology.

[254] Galanis E., Jaeckle K.A., Maurer M.J., Reid J.M., Ames M.M., Hardwick J.S., Reilly J.F., Loboda A., Nebozhyn M., Fantin V.R. (2009). Phase II Trial of Vorinostat in Recurrent Glioblastoma Multiforme: A North Central Cancer Treatment Group Study. J. Clin. Oncol..

[255] Iwamoto F.M., Lamborn K.R., Kuhn J.G., Wen P.Y., Yung W.K.A., Gilbert M.R., Chang S.M., Lieberman F.S., Prados M.D., Fine H.A. (2011). A Phase I/II Trial of the Histone Deacetylase Inhibitor Romidepsin for Adults with Recurrent Malignant Glioma: North American Brain Tumor Consortium Study 03-03. Neuro-Oncology.

[256] Bogdahn U., Hau P., Stockhammer G., Venkataramana N.K., Mahapatra A.K., Suri A., Balasubramaniam A., Nair S., Oliushine V., Parfenov V. (2011). Targeted Therapy for High-Grade Glioma with the TGF-Β2 Inhibitor Trabedersen: Results of a Randomized and Controlled Phase IIb Study. Neuro-Oncology.

[257] Wick A., Desjardins A., Suarez C., Forsyth P., Gueorguieva I., Burkholder T., Cleverly A.L., Estrem S.T., Wang S., Lahn M.M. (2020). Phase 1b/2a Study of Galunisertib, a Small Molecule Inhibitor of Transforming Growth Factor-Beta Receptor I, in Combination with Standard Temozolomide-Based Radiochemotherapy in Patients with Newly Diagnosed Malignant Glioma. Investig. New Drugs.

[258] Blakeley J.O., Grossman S.A., Chi A.S., Mikkelsen T., Rosenfeld M.R., Ahluwalia M.S., Nabors L.B., Eichler A., Ribas I.G., Desideri S. (2019). Phase II Study of Iniparib with Concurrent Chemoradiation in Patients with Newly Diagnosed Glioblastoma. Clin. Cancer Res..

[259] Robins H.I., Zhang P., Gilbert M.R., Chakravarti A., de Groot J.F., Grimm S.A., Wang F., Lieberman F.S., Krauze A., Trotti A.M. (2016). A Randomized Phase I/II Study of ABT-888 in Combination with Temozolomide in Recurrent Temozolomide Resistant Glioblastoma: An NRG Oncology RTOG Group Study. J. Neurooncol..

[260] Cao Y., Li X., Kong S., Shang S., Qi Y. (2020). CDK4/6 Inhibition Suppresses Tumour Growth and Enhances the Effect of Temozolomide in Glioma Cells. J. Cell. Mol. Med..

[261] Sobhani N., D’Angelo A., Pittacolo M., Roviello G., Miccoli A., Corona S.P., Bernocchi O., Generali D., Otto T. (2019). Updates on the CDK4/6 Inhibitory Strategy and Combinations in Breast Cancer. Cells.

[262] Roeten M.S.F., Cloos J., Jansen G. (2018). Positioning of Proteasome Inhibitors in Therapy of Solid Malignancies. Cancer Chemother. Pharmacol..

[263] Huang W.-J., Chen W.-W., Zhang X. (2017). Proteasome Inhibitors in Glioblastoma. Oncol. Lett..

[264] Di K., Lloyd G.K., Abraham V., MacLaren A., Burrows F.J., Desjardins A., Trikha M., Bota D.A. (2016). Marizomib Activity as a Single Agent in Malignant Gliomas: Ability to Cross the Blood-Brain Barrier. Neuro-Oncology.

[265] Roth P., Mason W.P., Richardson P.G., Weller M. (2020). Proteasome Inhibition for the Treatment of Glioblastoma. Expert Opin. Investig. Drugs.

[266] Ceccacci E., Minucci S. (2016). Inhibition of Histone Deacetylases in Cancer Therapy: Lessons from Leukaemia. Br. J. Cancer.

[267] Lee P., Murphy B., Miller R., Menon V., Banik N.L., Giglio P., Lindhorst S.M., Varma A.K., Vandergrift W.A., Patel S.J. (2015). Mechanisms and Clinical Significance of Histone Deacetylase Inhibitors: Epigenetic Glioblastoma Therapy. Anticancer Res..

[268] Chen R., Zhang M., Zhou Y., Guo W., Yi M., Zhang Z., Ding Y., Wang Y. (2020). The Application of Histone Deacetylases Inhibitors in Glioblastoma. J. Exp. Clin. Cancer Res. CR.

[269] Han J., Alvarez-Breckenridge C.A., Wang Q.-E., Yu J. (2015). TGF-β Signaling and Its Targeting for Glioma Treatment. Am. J. Cancer Res..

[270] Birch J.L., Coull B.J., Spender L.C., Watt C., Willison A., Syed N., Chalmers A.J., Hossain-Ibrahim M.K., Inman G.J. (2020). Multifaceted Transforming Growth Factor-Beta (TGFβ) Signalling in Glioblastoma. Cell. Signal..

[271] Towner R.A., Zalles M., Saunders D., Smith N. (2020). Novel Approaches to Combat Chemoresistance against Glioblastomas. Cancer Drug Resist..

[272] Sawyer J.S., Anderson B.D., Beight D.W., Campbell R.M., Jones M.L., Herron D.K., Lampe J.W., McCowan J.R., McMillen W.T., Mort N. (2003). Synthesis and Activity of New Aryl- and Heteroaryl-Substituted Pyrazole Inhibitors of the Transforming Growth Factor-Beta Type I Receptor Kinase Domain. J. Med. Chem..

[273] Murnyák B., Kouhsari M.C., Hershkovitch R., Kálmán B., Marko-Varga G., Klekner Á., Hortobágyi T. (2017). PARP1 Expression and Its Correlation with Survival Is Tumour Molecular Subtype Dependent in Glioblastoma. Oncotarget.

[274] Blakeley J.O., Grossman S.A., Mikkelsen T., Rosenfeld M.R., Peereboom D., Nabors L.B., Chi A.S., Emmons G., Ribas I.G., Supko J.G. (2015). Phase I Study of Iniparib Concurrent with Monthly or Continuous Temozolomide Dosing Schedules in Patients with Newly Diagnosed Malignant Gliomas. J. Neurooncol..

[275] Onishi M., Kurozumi K., Ichikawa T., Date I. (2013). Mechanisms of Tumor Development and Anti-Angiogenic Therapy in Glioblastoma Multiforme. Neurol. Med. Chir..

[276] Friedman H.S., Prados M.D., Wen P.Y., Mikkelsen T., Schiff D., Abrey L.E., Yung W.K.A., Paleologos N., Nicholas M.K., Jensen R. (2009). Bevacizumab Alone and in Combination with Irinotecan in Recurrent Glioblastoma. J. Clin. Oncol..

[277] Vredenburgh J.J., Desjardins A., Herndon J.E., Marcello J., Reardon D.A., Quinn J.A., Rich J.N., Sathornsumetee S., Gururangan S., Sampson J. (2007). Bevacizumab plus Irinotecan in Recurrent Glioblastoma Multiforme. J. Clin. Oncol..

[278] Brandes A.A., Gil-Gil M., Saran F., Carpentier A.F., Nowak A.K., Mason W., Zagonel V., Dubois F., Finocchiaro G., Fountzilas G. (2019). A Randomized Phase II Trial (TAMIGA) Evaluating the Efficacy and Safety of Continuous Bevacizumab Through Multiple Lines of Treatment for Recurrent Glioblastoma. Oncologist.

[279] Peters K.B., Lou E., Desjardins A., Reardon D.A., Lipp E.S., Miller E., Herndon J.E., McSherry F., Friedman H.S., Vredenburgh J.J. (2015). Phase II Trial of Upfront Bevacizumab, Irinotecan, and Temozolomide for Unresectable Glioblastoma. Oncologist.

[280] Wirsching H.-G., Tabatabai G., Roelcke U., Hottinger A.F., Jörger F., Schmid A., Plasswilm L., Schrimpf D., Mancao C., Capper D. (2018). Bevacizumab plus Hypofractionated Radiotherapy versus Radiotherapy Alone in Elderly Patients with Glioblastoma: The Randomized, Open-Label, Phase II ARTE Trial. Ann. Oncol..

[281] Reyes-Botero G., Cartalat-Carel S., Chinot O.L., Barrie M., Taillandier L., Beauchesne P., Catry-Thomas I., Barrière J., Guillamo J.-S., Fabbro M. (2018). Temozolomide Plus Bevacizumab in Elderly Patients with Newly Diagnosed Glioblastoma and Poor Performance Status: An ANOCEF Phase II Trial (ATAG). Oncologist.

[282] Ghiaseddin A., Reardon D., Massey W., Mannerino A., Lipp E.S., Herndon J.E., McSherry F., Desjardins A., Randazzo D., Friedman H.S. (2018). Phase II Study of Bevacizumab and Vorinostat for Patients with Recurrent World Health Organization Grade 4 Malignant Glioma. Oncologist.

[283] Vredenburgh J.J., Desjardins A., Herndon J.E., Dowell J.M., Reardon D.A., Quinn J.A., Rich J.N., Sathornsumetee S., Gururangan S., Wagner M. (2007). Phase II Trial of Bevacizumab and Irinotecan in Recurrent Malignant Glioma. Clin. Cancer Res..

[284] Ney D.E., Carlson J.A., Damek D.M., Gaspar L.E., Kavanagh B.D., Kleinschmidt-DeMasters B.K., Waziri A.E., Lillehei K.O., Reddy K., Chen C. (2015). Phase II Trial of Hypofractionated Intensity-Modulated Radiation Therapy Combined with Temozolomide and Bevacizumab for Patients with Newly Diagnosed Glioblastoma. J. Neurooncol..

[285] Balana C., De Las Penas R., Sepúlveda J.M., Gil-Gil M.J., Luque R., Gallego O., Carrato C., Sanz C., Reynes G., Herrero A. (2016). Bevacizumab and Temozolomide versus Temozolomide Alone as Neoadjuvant Treatment in Unresected Glioblastoma: The GENOM 009 Randomized Phase II Trial. J. Neurooncol..

[286] Chauffert B., Feuvret L., Bonnetain F., Taillandier L., Frappaz D., Taillia H., Schott R., Honnorat J., Fabbro M., Tennevet I. (2014). Randomized Phase II Trial of Irinotecan and Bevacizumab as Neo-Adjuvant and Adjuvant to Temozolomide-Based Chemoradiation Compared with Temozolomide-Chemoradiation for Unresectable Glioblastoma: Final Results of the TEMAVIR Study from ANOCEF. Ann. Oncol..

[287] Chinot O.L., Wick W., Mason W., Henriksson R., Saran F., Nishikawa R., Carpentier A.F., Hoang-Xuan K., Kavan P., Cernea D. (2014). Bevacizumab plus Radiotherapy–Temozolomide for Newly Diagnosed Glioblastoma. N. Engl. J. Med..

[288] Weathers S.-P., Han X., Liu D.D., Conrad C.A., Gilbert M.R., Loghin M.E., O’Brien B.J., Penas-Prado M., Puduvalli V.K., Tremont-Lukats I. (2016). A Randomized Phase II Trial of Standard Dose Bevacizumab versus Low Dose Bevacizumab plus Lomustine (CCNU) in Adults with Recurrent Glioblastoma. J. Neurooncol..

[289] Badruddoja M.A., Pazzi M., Sanan A., Schroeder K., Kuzma K., Norton T., Scully T., Mahadevan D., Ahmadi M.M. (2017). Phase II Study of Bi-Weekly Temozolomide plus Bevacizumab for Adult Patients with Recurrent Glioblastoma. Cancer Chemother. Pharmacol..

[290] Brandes A.A., Finocchiaro G., Zagonel V., Reni M., Caserta C., Fabi A., Clavarezza M., Maiello E., Eoli M., Lombardi G. (2016). AVAREG: A Phase II, Randomized, Noncomparative Study of Fotemustine or Bevacizumab for Patients with Recurrent Glioblastoma. Neuro-Oncology.

[291] Herrlinger U., Schäfer N., Steinbach J.P., Weyerbrock A., Hau P., Goldbrunner R., Friedrich F., Rohde V., Ringel F., Schlegel U. (2016). Bevacizumab Plus Irinotecan Versus Temozolomide in Newly Diagnosed O6-Methylguanine-DNA Methyltransferase Nonmethylated Glioblastoma: The Randomized GLARIUS Trial. J. Clin. Oncol..

[292] Wick W., Gorlia T., Bendszus M., Taphoorn M., Sahm F., Harting I., Brandes A.A., Taal W., Domont J., Idbaih A. (2017). Lomustine and Bevacizumab in Progressive Glioblastoma. N. Engl. J. Med..

[293] Cloughesy T.F., Brenner A., de Groot J.F., Butowski N.A., Zach L., Campian J.L., Ellingson B.M., Freedman L.S., Cohen Y.C., Lowenton-Spier N. (2020). A Randomized Controlled Phase III Study of VB-111 Combined with Bevacizumab vs Bevacizumab Monotherapy in Patients with Recurrent Glioblastoma (GLOBE). Neuro-Oncology.

[294] Desjardins A., Reardon D.A., Coan A., Marcello J., Herndon J.E., Bailey L., Peters K.B., Friedman H.S., Vredenburgh J.J. (2012). Bevacizumab and Daily Temozolomide for Recurrent Glioblastoma. Cancer.

[295] Vredenburgh J.J., Desjardins A., Reardon D.A., Peters K.B., Herndon J.E., Marcello J., Kirkpatrick J.P., Sampson J.H., Bailey L., Threatt S. (2011). The Addition of Bevacizumab to Standard Radiation Therapy and Temozolomide Followed by Bevacizumab, Temozolomide and Irinotecan for Newly Diagnosed Glioblastoma. Clin. Cancer Res..

[296] Reardon D.A., Desjardins A., Vredenburgh J.J., Gururangan S., Sampson J.H., Sathornsumetee S., McLendon R.E., Herndon J.E., Marcello J.E., Norfleet J. (2009). Metronomic Chemotherapy with Daily, Oral Etoposide plus Bevacizumab for Recurrent Malignant Glioma: A Phase II Study. Br. J. Cancer.

[297] Gilbert M.R., Dignam J.J., Armstrong T.S., Wefel J.S., Blumenthal D.T., Vogelbaum M.A., Colman H., Chakravarti A., Pugh S., Won M. (2014). A Randomized Trial of Bevacizumab for Newly Diagnosed Glioblastoma. N. Engl. J. Med..

[298] Iwamoto F.M., Lamborn K.R., Robins H.I., Mehta M.P., Chang S.M., Butowski N.A., Deangelis L.M., Abrey L.E., Zhang W.-T., Prados M.D. (2010). Phase II Trial of Pazopanib (GW786034), an Oral Multi-Targeted Angiogenesis Inhibitor, for Adults with Recurrent Glioblastoma (North American Brain Tumor Consortium Study 06-02). Neuro-Oncology.

[299] Brown N., McBain C., Nash S., Hopkins K., Sanghera P., Saran F., Phillips M., Dungey F., Clifton-Hadley L., Wanek K. (2016). Multi-Center Randomized Phase II Study Comparing Cediranib plus Gefitinib with Cediranib plus Placebo in Subjects with Recurrent/Progressive Glioblastoma. PLoS ONE.

[300] Batchelor T.T., Mulholland P., Neyns B., Nabors L.B., Campone M., Wick A., Mason W., Mikkelsen T., Phuphanich S., Ashby L.S. (2013). Phase III Randomized Trial Comparing the Efficacy of Cediranib as Monotherapy, and in Combination with Lomustine, versus Lomustine Alone in Patients with Recurrent Glioblastoma. J. Clin. Oncol..

[301] Batchelor T.T., Duda D.G., di Tomaso E., Ancukiewicz M., Plotkin S.R., Gerstner E., Eichler A.F., Drappatz J., Hochberg F.H., Benner T. (2010). Phase II Study of Cediranib, an Oral Pan-Vascular Endothelial Growth Factor Receptor Tyrosine Kinase Inhibitor, in Patients with Recurrent Glioblastoma. J. Clin. Oncol..

[302] Muhic A., Poulsen H.S., Sorensen M., Grunnet K., Lassen U. (2013). Phase II Open-Label Study of Nintedanib in Patients with Recurrent Glioblastoma Multiforme. J. Neurooncol..

[303] Norden A.D., Schiff D., Ahluwalia M.S., Lesser G.J., Nayak L., Lee E.Q., Rinne M.L., Muzikansky A., Dietrich J., Purow B. (2015). Phase II Trial of Triple Tyrosine Kinase Receptor Inhibitor Nintedanib in Recurrent High-Grade Gliomas. J. Neurooncol..

[304] Gerstner E.R., Eichler A.F., Plotkin S.R., Drappatz J., Doyle C.L., Xu L., Duda D.G., Wen P.Y., Jain R.K., Batchelor T.T. (2011). Phase I Trial with Biomarker Studies of Vatalanib (PTK787) in Patients with Newly Diagnosed Glioblastoma Treated with Enzyme Inducing Anti-Epileptic Drugs and Standard Radiation and Temozolomide. J. Neurooncol..

[305] Kalpathy-Cramer J., Chandra V., Da X., Ou Y., Emblem K.E., Muzikansky A., Cai X., Douw L., Evans J.G., Dietrich J. (2017). Phase II Study of Tivozanib, an Oral VEGFR Inhibitor, in Patients with Recurrent Glioblastoma. J. Neurooncol..

[306] Reardon D.A., Desjardins A., Peters K.B., Gururangan S., Sampson J.H., McLendon R.E., Herndon J.E., Bulusu A., Threatt S., Friedman A.H. (2012). Phase II Study of Carboplatin, Irinotecan, and Bevacizumab for Bevacizumab Naïve, Recurrent Glioblastoma. J. Neurooncol..

[307] Dirven L., van den Bent M.J., Bottomley A., van der Meer N., van der Holt B., Vos M.J., Walenkamp A.M.E., Beerepoot L.V., Hanse M.C.J., Reijneveld J.C. (2015). The Impact of Bevacizumab on Health-Related Quality of Life in Patients Treated for Recurrent Glioblastoma: Results of the Randomised Controlled Phase 2 BELOB Trial. Eur. J. Cancer.

[308] Taal W., Oosterkamp H.M., Walenkamp A.M.E., Dubbink H.J., Beerepoot L.V., Hanse M.C.J., Buter J., Honkoop A.H., Boerman D., de Vos F.Y.F. (2014). Single-Agent Bevacizumab or Lomustine versus a Combination of Bevacizumab plus Lomustine in Patients with Recurrent Glioblastoma (BELOB Trial): A Randomised Controlled Phase 2 Trial. Lancet Oncol..

[309] Schnell O., Thorsteinsdottir J., Fleischmann D.F., Lenski M., Abenhardt W., Giese A., Tonn J.-C., Belka C., Kreth F.W., Niyazi M. (2016). Re-Irradiation Strategies in Combination with Bevacizumab for Recurrent Malignant Glioma. J. Neurooncol..

[310] Niyazi M., Harter P.N., Hattingen E., Rottler M., von Baumgarten L., Proescholdt M., Belka C., Lauber K., Mittelbronn M. (2016). Bevacizumab and Radiotherapy for the Treatment of Glioblastoma: Brothers in Arms or Unholy Alliance?. Oncotarget.

[311] Chinot O.L., de La Motte Rouge T., Moore N., Zeaiter A., Das A., Phillips H., Modrusan Z., Cloughesy T. (2011). AVAglio: Phase 3 Trial of Bevacizumab plus Temozolomide and Radiotherapy in Newly Diagnosed Glioblastoma Multiforme. Adv. Ther..

[312] van Linde M.E., Verhoeff J.J.C., Richel D.J., van Furth W.R., Reijneveld J.C., Verheul H.M.W., Stalpers L.J.A. (2015). Bevacizumab in Combination with Radiotherapy and Temozolomide for Patients with Newly Diagnosed Glioblastoma Multiforme. Oncologist.

[313] Sandmann T., Bourgon R., Garcia J., Li C., Cloughesy T., Chinot O.L., Wick W., Nishikawa R., Mason W., Henriksson R. (2015). Patients with Proneural Glioblastoma May Derive Overall Survival Benefit From the Addition of Bevacizumab to First-Line Radiotherapy and Temozolomide: Retrospective Analysis of the AVAglio Trial. J. Clin. Oncol..

[314] Brave S.R., Ratcliffe K., Wilson Z., James N.H., Ashton S., Wainwright A., Kendrew J., Dudley P., Broadbent N., Sproat G. (2011). Assessing the Activity of Cediranib, a VEGFR-2/3 Tyrosine Kinase Inhibitor, against VEGFR-1 and Members of the Structurally Related PDGFR Family. Mol. Cancer Ther..

[315] Schäfer N., Gielen G.H., Kebir S., Wieland A., Till A., Mack F., Schaub C., Tzaridis T., Reinartz R., Niessen M. (2016). Phase I Trial of Dovitinib (TKI258) in Recurrent Glioblastoma. J. Cancer Res. Clin. Oncol..

[316] Thanasupawat T., Natarajan S., Rommel A., Glogowska A., Bergen H., Krcek J., Pitz M., Beiko J., Krawitz S., Verma I.M. (2017). Dovitinib Enhances Temozolomide Efficacy in Glioblastoma Cells. Mol. Oncol..

[317] Reardon D.A., Egorin M.J., Desjardins A., Vredenburgh J.J., Beumer J.H., Lagattuta T.F., Gururangan S., Herndon J.E., Salvado A.J., Friedman H.S. (2009). Phase I Pharmacokinetic Study of the Vascular Endothelial Growth Factor Receptor Tyrosine Kinase Inhibitor Vatalanib (PTK787) plus Imatinib and Hydroxyurea for Malignant Glioma. Cancer.

[318] Cloughesy T., Finocchiaro G., Belda-Iniesta C., Recht L., Brandes A.A., Pineda E., Mikkelsen T., Chinot O.L., Balana C., Macdonald D.R. (2017). Randomized, Double-Blind, Placebo-Controlled, Multicenter Phase II Study of Onartuzumab Plus Bevacizumab Versus Placebo Plus Bevacizumab in Patients with Recurrent Glioblastoma: Efficacy, Safety, and Hepatocyte Growth Factor and O6-Methylguanine-DNA Methyltransferase Biomarker Analyses. J. Clin. Oncol..

[319] Wen P.Y., Drappatz J., de Groot J., Prados M.D., Reardon D.A., Schiff D., Chamberlain M., Mikkelsen T., Desjardins A., Holland J. (2018). Phase II Study of Cabozantinib in Patients with Progressive Glioblastoma: Subset Analysis of Patients Naive to Antiangiogenic Therapy. Neuro-Oncology.

[320] Wen P.Y., Schiff D., Cloughesy T.F., Raizer J.J., Laterra J., Smitt M., Wolf M., Oliner K.S., Anderson A., Zhu M. (2011). A Phase II Study Evaluating the Efficacy and Safety of AMG 102 (Rilotumumab) in Patients with Recurrent Glioblastoma. Neuro-Oncology.

[321] de Groot J.F., Lamborn K.R., Chang S.M., Gilbert M.R., Cloughesy T.F., Aldape K., Yao J., Jackson E.F., Lieberman F., Robins H.I. (2011). Phase II Study of Aflibercept in Recurrent Malignant Glioma: A North American Brain Tumor Consortium Study. J. Clin. Oncol..

[322] Blumenschein G.R., Mills G.B., Gonzalez-Angulo A.M. (2012). Targeting the Hepatocyte Growth Factor-CMET Axis in Cancer Therapy. J. Clin. Oncol..

[323] Garnett J., Chumbalkar V., Vaillant B., Gururaj A.E., Hill K.S., Latha K., Yao J., Priebe W., Colman H., Elferink L.A. (2013). Regulation of HGF Expression by ΔEGFR-Mediated c-Met Activation in Glioblastoma Cells. Neoplasia.

[324] Huang M., Liu T., Ma P., Mitteer R.A., Zhang Z., Kim H.J., Yeo E., Zhang D., Cai P., Li C. (2016). C-Met-Mediated Endothelial Plasticity Drives Aberrant Vascularization and Chemoresistance in Glioblastoma. J. Clin. Investig..

[325] Jahangiri A., De Lay M., Miller L.M., Carbonell W.S., Hu Y.-L., Lu K., Tom M.W., Paquette J., Tokuyasu T.A., Tsao S. (2013). Gene Expression Profile Identifies Tyrosine Kinase C-Met as a Targetable Mediator of Antiangiogenic Therapy Resistance. Clin. Cancer Res..

[326] Lu K.V., Bergers G. (2013). Mechanisms of Evasive Resistance to Anti-VEGF Therapy in Glioblastoma. CNS Oncol..

[327] Das A., Cheng R.R., Hilbert M.L.T., Dixon-Moh Y.N., Decandio M., Vandergrift W.A., Banik N.L., Lindhorst S.M., Cachia D., Varma A.K. (2015). Synergistic Effects of Crizotinib and Temozolomide in Experimental FIG-ROS1 Fusion-Positive Glioblastoma. Cancer Growth Metastasis.

[328] Schiff D., Desjardins A., Cloughesy T., Mikkelsen T., Glantz M., Chamberlain M.C., Reardon D.A., Wen P.Y. (2016). Phase 1 Dose Escalation Trial of the Safety and Pharmacokinetics of Cabozantinib Concurrent with Temozolomide and Radiotherapy or Temozolomide after Radiotherapy in Newly Diagnosed Patients with High-Grade Gliomas. Cancer.

[329] Schneider K., Weyerbrock A., Doostkam S., Plate K., Machein M.R. (2015). Lack of Evidence for PlGF Mediating the Tumor Resistance after Anti-Angiogenic Therapy in Malignant Gliomas. J. Neurooncol..

[330] Lassen U., Chinot O.L., McBain C., Mau-Sørensen M., Larsen V.A., Barrie M., Roth P., Krieter O., Wang K., Habben K. (2015). Phase 1 Dose-Escalation Study of the Antiplacental Growth Factor Monoclonal Antibody RO5323441 Combined with Bevacizumab in Patients with Recurrent Glioblastoma. Neuro-Oncology.

[331] Tabouret E., Denicolai E., Delfino C., Graillon T., Boucard C., Nanni I., Padovani L., Figarella-Branger D., Chinot O. (2016). Changes in PlGF and MET-HGF Expressions in Paired Initial and Recurrent Glioblastoma. J. Neurooncol..

[332] Rosen L.S., Gordon M.S., Robert F., Matei D.E. (2014). Endoglin for Targeted Cancer Treatment. Curr. Oncol. Rep..

[333] Ahluwalia M.S., Rogers L.R., Chaudhary R.T., Newton H.B., Seon B.K., Jivani M.A., Adams B.J., Shazer R.L., Theuer C.P. (2016). A Phase 2 Trial of TRC105 with Bevacizumab for Bevacizumab Refractory Glioblastoma. J. Clin. Oncol..

[334] Galanis E., Anderson S.K., Butowski N.A., Hormigo A., Schiff D., Tran D.D., Omuro A.M.P., Jaeckle K.A., Kumar S., Kaufmann T.J. (2017). NCCTG N1174: Phase I/Comparative Randomized Phase (Ph) II Trial of TRC105 plus Bevacizumab versus Bevacizumab in Recurrent Glioblastoma (GBM) (Alliance). J. Clin. Oncol..

[335] Afshar Moghaddam N., Mahsuni P., Taheri D. (2015). Evaluation of Endoglin as an Angiogenesis Marker in Glioblastoma. Iran. J. Pathol..

[336] Puduvalli V.K., Giglio P., Groves M.D., Hess K.R., Gilbert M.R., Mahankali S., Jackson E.F., Levin V.A., Conrad C.A., Hsu S.H. (2008). Phase II Trial of Irinotecan and Thalidomide in Adults with Recurrent Glioblastoma Multiforme. Neuro-Oncology.

[337] Fadul C.E., Kingman L.S., Meyer L.P., Cole B.F., Eskey C.J., Rhodes C.H., Roberts D.W., Newton H.B., Pipas J.M. (2008). A Phase II Study of Thalidomide and Irinotecan for Treatment of Glioblastoma Multiforme. J. Neurooncol..

[338] Kesari S., Schiff D., Henson J.W., Muzikansky A., Gigas D.C., Doherty L., Batchelor T.T., Longtine J.A., Ligon K.L., Weaver S. (2008). Phase II Study of Temozolomide, Thalidomide, and Celecoxib for Newly Diagnosed Glioblastoma in Adults. Neuro-Oncology.

[339] Groves M.D., Puduvalli V.K., Chang S.M., Conrad C.A., Gilbert M.R., Tremont-Lukats I.W., Liu T.-J., Peterson P., Schiff D., Cloughesy T.F. (2007). A North American Brain Tumor Consortium (NABTC 99-04) Phase II Trial of Temozolomide plus Thalidomide for Recurrent Glioblastoma Multiforme. J. Neurooncol..

[340] Stupp R., Hegi M.E., Gorlia T., Erridge S.C., Perry J., Hong Y.-K., Aldape K.D., Lhermitte B., Pietsch T., Grujicic D. (2014). Cilengitide Combined with Standard Treatment for Patients with Newly Diagnosed Glioblastoma with Methylated MGMT Promoter (CENTRIC EORTC 26071-22072 Study): A Multicentre, Randomised, Open-Label, Phase 3 Trial. Lancet Oncol..

[341] Nabors L.B., Fink K.L., Mikkelsen T., Grujicic D., Tarnawski R., Nam D.H., Mazurkiewicz M., Salacz M., Ashby L., Zagonel V. (2015). Two Cilengitide Regimens in Combination with Standard Treatment for Patients with Newly Diagnosed Glioblastoma and Unmethylated MGMT Gene Promoter: Results of the Open-Label, Controlled, Randomized Phase II CORE Study. Neuro-Oncology.

[342] Nabors L.B., Mikkelsen T., Hegi M.E., Ye X., Batchelor T., Lesser G., Peereboom D., Rosenfeld M.R., Olsen J., Brem S. (2012). A Safety Run-in and Randomized Phase 2 Study of Cilengitide Combined with Chemoradiation for Newly Diagnosed Glioblastoma (NABTT 0306). Cancer.

[343] Reardon D.A., Fink K.L., Mikkelsen T., Cloughesy T.F., O’Neill A., Plotkin S., Glantz M., Ravin P., Raizer J.J., Rich K.M. (2008). Randomized Phase II Study of Cilengitide, an Integrin-Targeting Arginine-Glycine-Aspartic Acid Peptide, in Recurrent Glioblastoma Multiforme. J. Clin. Oncol..

[344] Elinzano H., Hebda N., Luppe D., Turchetti W., Rosati K., Sikov W.M., Safran H. (2016). PSMA ADC for Progressive Glioblastoma: Phase II Brown University Oncology Research Group Study. J. Clin. Oncol..

[345] Riva M., Imbesi F., Beghi E., Galli C., Citterio A., Trapani P., Sterzi R., Collice M. (2007). Temozolomide and Thalidomide in the Treatment of Glioblastoma Multiforme. Anticancer Res..

[346] Alexander B.M., Wang M., Yung W.K.A., Fine H.A., Donahue B.A., Tremont I.W., Richards R.S., Kerlin K.J., Hartford A.C., Curran W.J. (2013). A Phase II Study of Conventional Radiation Therapy and Thalidomide for Supratentorial, Newly-Diagnosed Glioblastoma (RTOG 9806). J. Neurooncol..

[347] Stupp R., Hegi M.E., Neyns B., Goldbrunner R., Schlegel U., Clement P.M.J., Grabenbauer G.G., Ochsenbein A.F., Simon M., Dietrich P.-Y. (2010). Phase I/IIa Study of Cilengitide and Temozolomide with Concomitant Radiotherapy Followed by Cilengitide and Temozolomide Maintenance Therapy in Patients with Newly Diagnosed Glioblastoma. J. Clin. Oncol..

[348] Cianfrocca M.E., Kimmel K.A., Gallo J., Cardoso T., Brown M.M., Hudes G., Lewis N., Weiner L., Lam G.N., Brown S.C. (2006). Phase 1 Trial of the Antiangiogenic Peptide ATN-161 (Ac-PHSCN-NH(2)), a Beta Integrin Antagonist, in Patients with Solid Tumours. Br. J. Cancer.

[349] Neal J., Wakelee H. (2010). AMG-386, a Selective Angiopoietin-1/-2-Neutralizing Peptibody for the Potential Treatment of Cancer. Curr. Opin. Mol. Ther..

[350] Walia A., Yang J.F., Huang Y.-H., Rosenblatt M.I., Chang J.-H., Azar D.T. (2015). Endostatin’s Emerging Roles in Angiogenesis, Lymphangiogenesis, Disease, and Clinical Applications. Biochim. Biophys. Acta.

[351] Chen J., Yao Q., Li D., Zhang J., Wang T., Yu M., Zhou X., Huan Y., Wang J., Wang L. (2013). Neoadjuvant Rh-Endostatin, Docetaxel and Epirubicin for Breast Cancer: Efficacy and Safety in a Prospective, Randomized, Phase II Study. BMC Cancer.

[352] Cui C., Mao L., Chi Z., Si L., Sheng X., Kong Y., Li S., Lian B., Gu K., Tao M. (2013). A Phase II, Randomized, Double-Blind, Placebo-Controlled Multicenter Trial of Endostar in Patients with Metastatic Melanoma. Mol. Ther..

[353] Zhao X., Su Y., You J., Gong L., Zhang Z., Wang M., Zhao Z., Zhang Z., Li X., Wang C. (2016). Combining Antiangiogenic Therapy with Neoadjuvant Chemotherapy Increases Treatment Efficacy in Stage IIIA (N2) Non-Small Cell Lung Cancer without Increasing Adverse Effects. Oncotarget.

[354] Saffar H., Noohi M., Tavangar S.M., Saffar H., Azimi S. (2018). Expression of Prostate-Specific Membrane Antigen (PSMA) in Brain Glioma and Its Correlation with Tumor Grade. Iran. J. Pathol..

[355] Anilkumar G., Barwe S.P., Christiansen J.J., Rajasekaran S.A., Kohn D.B., Rajasekaran A.K. (2006). Association of Prostate-Specific Membrane Antigen with Caveolin-1 and Its Caveolae-Dependent Internalization in Microvascular Endothelial Cells: Implications for Targeting to Tumor Vasculature. Microvasc. Res..

[356] Hatoum A., Mohammed R., Zakieh O. The Unique Invasiveness of Glioblastoma and Possible Drug Targets on Extracellular Matrix. https://www.dovepress.com/the-unique-invasiveness-of-glioblastoma-and-possible-drug-targets-on-e-peer-reviewed-fulltext-article-CMAR.

[357] Rempe R.G., Hartz A.M., Bauer B. (2016). Matrix Metalloproteinases in the Brain and Blood–Brain Barrier: Versatile Breakers and Makers. J. Cereb. Blood Flow Metab..

[358] Mandel J.J., Yust-Katz S., Patel A.J., Cachia D., Liu D., Park M., Yuan Y., Kent T.A., de Groot J.F. (2018). Inability of Positive Phase II Clinical Trials of Investigational Treatments to Subsequently Predict Positive Phase III Clinical Trials in Glioblastoma. Neuro-Oncology.

[359] Ventz S., Lai A., Cloughesy T.F., Wen P.Y., Trippa L., Alexander B.M. (2019). Design and Evaluation of an External Control Arm Using Prior Clinical Trials and Real-World Data. Clin. Cancer Res..

[360] Lee E.Q., Chukwueke U.N., Hervey-Jumper S.L., de Groot J.F., Leone J.P., Armstrong T.S., Chang S.M., Arons D., Oliver K., Verble K. (2019). Barriers to Accrual and Enrollment in Brain Tumor Trials. Neuro-Oncology.

[361] Parker J.L., Kuzulugil S.S., Pereverzev K., Mac S., Lopes G., Shah Z., Weerasinghe A., Rubinger D., Falconi A., Bener A. (2021). Does Biomarker Use in Oncology Improve Clinical Trial Failure Risk? A Large-Scale Analysis. Cancer Med..

[362] Weller M., van den Bent M., Preusser M., Le Rhun E., Tonn J.C., Minniti G., Bendszus M., Balana C., Chinot O., Dirven L. (2021). EANO Guidelines on the Diagnosis and Treatment of Diffuse Gliomas of Adulthood. Nat. Rev. Clin. Oncol..

[363] Sestito S., Runfola M., Tonelli M., Chiellini G., Rapposelli S. (2018). New Multitarget Approaches in the War Against Glioblastoma: A Mini-Perspective. Front. Pharmacol..

[364] Wolbers J.G. (2014). Novel Strategies in Glioblastoma Surgery Aim at Safe, Supra-Maximum Resection in Conjunction with Local Therapies. Chin. J. Cancer.

[365] Sanai N., Polley M.-Y., McDermott M.W., Parsa A.T., Berger M.S. (2011). An Extent of Resection Threshold for Newly Diagnosed Glioblastomas: Clinical Article. J. Neurosurg..

[366] Trifiletti D.M., Alonso C., Grover S., Fadul C.E., Sheehan J.P., Showalter T.N. (2017). Prognostic Implications of Extent of Resection in Glioblastoma: Analysis from a Large Database. World Neurosurg..

[367] Jena L., McErlean E., McCarthy H. (2020). Delivery across the Blood-Brain Barrier: Nanomedicine for Glioblastoma Multiforme. Drug Deliv. Transl. Res..

[368] Shi M., Sanche L. (2019). Convection-Enhanced Delivery in Malignant Gliomas: A Review of Toxicity and Efficacy. J. Oncol..

[369] van den Bent M.J., Gao Y., Kerkhof M., Kros J.M., Gorlia T., van Zwieten K., Prince J., van Duinen S., Sillevis Smitt P.A., Taphoorn M. (2015). Changes in the EGFR Amplification and EGFRvIII Expression between Paired Primary and Recurrent Glioblastomas. Neuro-Oncology.

[370] Wang J., Cazzato E., Ladewig E., Frattini V., Rosenbloom D.I.S., Zairis S., Abate F., Liu Z., Elliott O., Shin Y.-J. (2016). Clonal Evolution of Glioblastoma under Therapy. Nat. Genet..

[371] Nathanson D.A., Gini B., Mottahedeh J., Visnyei K., Koga T., Gomez G., Eskin A., Hwang K., Wang J., Masui K. (2014). Targeted Therapy Resistance Mediated by Dynamic Regulation of Extrachromosomal Mutant EGFR DNA. Science.

[372] Huang L., Fu L. (2015). Mechanisms of Resistance to EGFR Tyrosine Kinase Inhibitors. Acta Pharm. Sin. B.

[373] An Z., Aksoy O., Zheng T., Fan Q.-W., Weiss W.A. (2018). Epidermal Growth Factor Receptor and EGFRvIII in Glioblastoma: Signaling Pathways and Targeted Therapies. Oncogene.

[374] Goodwin C.R., Rath P., Oyinlade O., Lopez H., Mughal S., Xia S., Li Y., Kaur H., Zhou X., Ahmed A.K. (2018). Crizotinib and Erlotinib Inhibits Growth of C-Met+/EGFRvIII+ Primary Human Glioblastoma Xenografts. Clin. Neurol. Neurosurg..

[375] Akhavan D., Pourzia A.L., Nourian A.A., Williams K.J., Nathanson D., Babic I., Villa G.R., Tanaka K., Nael A., Yang H. (2013). De-Repression of PDGFRβ Transcription Promotes Acquired Resistance to EGFR Tyrosine Kinase Inhibitors in Glioblastoma Patients. Cancer Discov..

[376] Mellinghoff I.K., Wang M.Y., Vivanco I., Haas-Kogan D.A., Zhu S., Dia E.Q., Lu K.V., Yoshimoto K., Huang J.H.Y., Chute D.J. (2005). Molecular Determinants of the Response of Glioblastomas to EGFR Kinase Inhibitors. N. Engl. J. Med..

[377] Bhat K.P.L., Balasubramaniyan V., Vaillant B., Ezhilarasan R., Hummelink K., Hollingsworth F., Wani K., Heathcock L., James J.D., Goodman L.D. (2013). Mesenchymal Differentiation Mediated by NF-ΚB Promotes Radiation Resistance in Glioblastoma. Cancer Cell.

[378] Halliday J., Helmy K., Pattwell S.S., Pitter K.L., LaPlant Q., Ozawa T., Holland E.C. (2014). In Vivo Radiation Response of Proneural Glioma Characterized by Protective P53 Transcriptional Program and Proneural-Mesenchymal Shift. Proc. Natl. Acad. Sci. USA.

[379] Ghosh D., Nandi S., Bhattacharjee S. (2018). Combination Therapy to Checkmate Glioblastoma: Clinical Challenges and Advances. Clin. Transl. Med..

[380] Eskilsson E., Røsland G.V., Solecki G., Wang Q., Harter P.N., Graziani G., Verhaak R.G.W., Winkler F., Bjerkvig R., Miletic H. (2018). EGFR Heterogeneity and Implications for Therapeutic Intervention in Glioblastoma. Neuro-Oncology.

[381] Burkhardt J.-K., Riina H., Shin B.J., Christos P., Kesavabhotla K., Hofstetter C.P., Tsiouris A.J., Boockvar J.A. (2012). Intra-Arterial Delivery of Bevacizumab after Blood-Brain Barrier Disruption for the Treatment of Recurrent Glioblastoma: Progression-Free Survival and Overall Survival. World Neurosurg..

[382] Boockvar J.A., Tsiouris A.J., Hofstetter C.P., Kovanlikaya I., Fralin S., Kesavabhotla K., Seedial S.M., Pannullo S.C., Schwartz T.H., Stieg P. (2011). Safety and Maximum Tolerated Dose of Superselective Intraarterial Cerebral Infusion of Bevacizumab after Osmotic Blood-Brain Barrier Disruption for Recurrent Malignant Glioma. Clinical Article. J. Neurosurg..

[383] Alter R.A., White T.G., Fanous A.A., Chakraborty S., Filippi C.G., Pisapia D.J., Tsiouris A.J., Boockvar J.A. (2017). Long-Term Benefit of Intra-Arterial Bevacizumab for Recurrent Glioblastoma. J. Exp. Ther. Oncol..

[384] Chakraborty S., Filippi C.G., Burkhardt J.-K., Fralin S., Ray A., Wong T., Ortiz R., Langer D.J., Boockvar J.A. (2016). Durability of Single Dose Intra-Arterial Bevacizumab after Blood/Brain Barrier Disruption for Recurrent Glioblastoma. J. Exp. Ther. Oncol..

[385] Heiland D.H., Masalha W., Franco P., Machein M.R., Weyerbrock A. (2016). Progression-Free and Overall Survival in Patients with Recurrent Glioblastoma Multiforme Treated with Last-Line Bevacizumab versus Bevacizumab/Lomustine. J. Neurooncol..

[386] Chinot O.L. (2014). Cilengitide in Glioblastoma: When Did It Fail?. Lancet Oncol..

[387] Stupp R., Picard M., Weller M. (2014). Does Cilengitide Deserve Another Chance?-Authors’ Reply. Lancet Oncol..

[388] Tucci M., Stucci S., Silvestris F. (2014). Does Cilengitide Deserve Another Chance?. Lancet Oncol..

[389] Reynolds A.R., Hart I.R., Watson A.R., Welti J.C., Silva R.G., Robinson S.D., Da Violante G., Gourlaouen M., Salih M., Jones M.C. (2009). Stimulation of Tumor Growth and Angiogenesis by Low Concentrations of RGD-Mimetic Integrin Inhibitors. Nat. Med..

[390] Eisele G., Wick A., Eisele A.-C., Clément P.M., Tonn J., Tabatabai G., Ochsenbein A., Schlegel U., Neyns B., Krex D. (2014). Cilengitide Treatment of Newly Diagnosed Glioblastoma Patients Does Not Alter Patterns of Progression. J. Neurooncol..

[391] Weller M., Nabors L.B., Gorlia T., Leske H., Rushing E., Bady P., Hicking C., Perry J., Hong Y.-K., Roth P. (2016). Cilengitide in Newly Diagnosed Glioblastoma: Biomarker Expression and Outcome. Oncotarget.

[392] Cosset É., Weis S.M., Cheresh D.A. (2018). Re-Thinking the Preclinical Development of GBM Therapeutics. Oncoscience.

[393] Cosset É., Ilmjärv S., Dutoit V., Elliott K., von Schalscha T., Camargo M.F., Reiss A., Moroishi T., Seguin L., Gomez G. (2017). Glut3 Addiction Is a Druggable Vulnerability for a Molecularly Defined Subpopulation of Glioblastoma. Cancer Cell.

[394] Uneda A., Kurozumi K., Fujimura A., Fujii K., Ishida J., Shimazu Y., Otani Y., Tomita Y., Hattori Y., Matsumoto Y. (2021). Differentiated Glioblastoma Cells Accelerate Tumor Progression by Shaping the Tumor Microenvironment via CCN1-Mediated Macrophage Infiltration. Acta Neuropathol. Commun..

[395] Wang X., Prager B.C., Wu Q., Kim L.J.Y., Gimple R.C., Shi Y., Yang K., Morton A.R., Zhou W., Zhu Z. (2018). Reciprocal Signaling between Glioblastoma Stem Cells and Differentiated Tumor Cells Promotes Malignant Progression. Cell Stem Cell.

[396] Petroni G., Buqué A., Zitvogel L., Kroemer G., Galluzzi L. (2021). Immunomodulation by Targeted Anticancer Agents. Cancer Cell.

[397] Martins T.A., Schmassmann P., Shekarian T., Boulay J.-L., Ritz M.-F., Zanganeh S., Vom Berg J., Hutter G. (2020). Microglia-Centered Combinatorial Strategies Against Glioblastoma. Front. Immunol..

[398] Fukumura D., Kloepper J., Amoozgar Z., Duda D.G., Jain R.K. (2018). Enhancing Cancer Immunotherapy Using Antiangiogenics: Opportunities and Challenges. Nat. Rev. Clin. Oncol..

[399] Song Y., Fu Y., Xie Q., Zhu B., Wang J., Zhang B. (2020). Anti-Angiogenic Agents in Combination with Immune Checkpoint Inhibitors: A Promising Strategy for Cancer Treatment. Front. Immunol..

